# Novel Indoline Spiropyrans Based on Human Hormones β-Estradiol and Estrone: Synthesis, Structure, Chromogenic and Cytotoxic Properties

**DOI:** 10.3390/molecules28093866

**Published:** 2023-05-04

**Authors:** Ilya V. Ozhogin, Artem D. Pugachev, Nadezhda I. Makarova, Anna A. Belanova, Anastasia S. Kozlenko, Irina A. Rostovtseva, Peter V. Zolotukhin, Oleg P. Demidov, Islam M. El-Sewify, Gennady S. Borodkin, Anatoly V. Metelitsa, Boris S. Lukyanov

**Affiliations:** 1 Institute of Physical and Organic Chemistry, Southern Federal University, 194/2 Stachka Ave., 344090 Rostov-on-Don, Russiaavmetelitsa@sfedu.ru (A.V.M.); bslukyanov@sfedu.ru (B.S.L.); 2 Academy of Biology and Biotechnology, Southern Federal University, 194/1 Stachka Ave., 344090 Rostov-on-Don, Russiap.zolotukhin@gmail.com (P.V.Z.); 3 Faculty of Chemistry and Pharmacy, North-Caucasus Federal University, 1 Pushkina Str., 355000 Stavropol, Russia; 4 Department of Chemistry, Faculty of Science, Ain Shams University, Cairo 11566, Egypt

**Keywords:** spiropyran, β-estradiol, molecular switch, acidochromism, cytotoxicity, single-crystal X-ray analysis

## Abstract

The introduction of a switchable function into the structure of a bioactive compound can endow it with unique capabilities for regulating biological activity under the influence of various types of external stimuli, which makes such hybrid compounds promising objects for photopharmacology, targeted drug delivery and bio-imaging. This work is devoted to the synthesis and study of new spirocyclic derivatives of important human hormones—β-estradiol and estrone—possessing a wide range of biological activities. The obtained hybrid compounds represent an indoline spiropyrans family, a widely known class of organic photochromic compounds. The structure of the compounds was confirmed by ^1^H and ^13^C NMR, IR, HRMS and single-crystal X-ray analysis. The intermolecular interactions in the crystals of spiropyran (**3**) were defined by Hirshfeld surfaces and 2D fingerprint plots, which were successfully acquired from CrystalExplorer (v21.5). All target hybrids demonstrated pronounced activity in the visible region of the spectrum. The mechanisms of thermal isomerization processes of spiropyrans and their protonated merocyanine forms were studied by DFT methods, which revealed the energetic advantage of the protonation process with the formation of a β-cisoid CCCH conformer at the first stage and its further isomerization to more stable β-transoid forms. The proposed mechanism of acidochromic transformation was confirmed by the additional NMR study data that allowed for the detecting of the intermediate CCCH isomer. The study of the short-term cytotoxicity of new spirocyclic derivatives of estrogens and their 2-formyl-precursors was performed on the HeLa cell model. The precursors and spiropyrans differed in toxicity, suggesting their variable applicability in novel anti-cancer technologies.

## 1. Introduction

β-Estradiol and estrone are the two main representatives of estrogens, a subclass of female sex hormones produced in the human body. In addition to the hormonal effects, β-estradiol and estrone have a wide range of biological activities; for example, they demonstrate anti-atherosclerotic and cardioprotective effects, can lower blood cholesterol levels, possess anti-inflammatory and antioxidant properties, and, in general, take an active part in the control of energy balance and homeostasis of glucose in the body [1,2,3,4,5,6]. Recent studies highlighted even the potential protective effect of estrogens against COVID-19 due to their anti-inflammatory and immunomodulatory properties [7,8].

Many estrogen derivatives possess anticancer activity against various types of cancer cells [9,10,11,12,13]. Estradiol and its metabolites demonstrate an antiproliferative effect comparable to tamoxifen against both estrogen-dependent and estrogen-independent cancer cell lines, including breast tumor cells MCF-7 and MDA-MB 231 [14].

Particular attention is drawn to some estrogenic metabolites and their synthetic analogues that do not have hormonal activity. Thus, 2-methoxyestradiol (2ME_2_) and its derivatives have been attracting the attention of scientists for the past two decades as anti-cancer chemotherapeutic agents [15,16,17,18,19]. Their activity is usually attributed to antitubulin, antiangiogenic, proapoptotic and ROS-inducing properties. E. Nolte et al. showed that exposure of breast and lung cancer cells to a novel analog of 2ME_2_ prior to gamma-radiation enhances Bcl-2-mediated cell death [20]. At the same time, 2-hydroxy and 2-methoxy-estradiol derivatives exhibit positive effects on the cardiovascular and nervous systems [3,5].

**Scheme 1:**
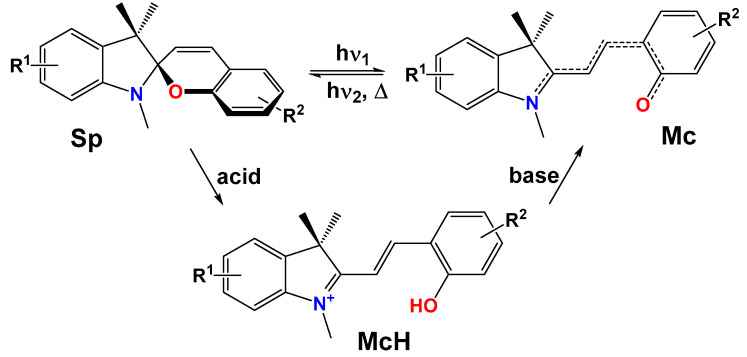
General scheme of photo- and acidochromic transformations of indoline spiropyrans.

The aim of this work was to obtain and study novel spirocyclic derivatives of β-estradiol and estrone containing a spiro-condensed indoline fragment and representing a class of indoline spiropyrans. Spiropyrans (SPPs) are one of the most exciting and promising classes of organic photochromic compounds capable of switching between different forms under the action of light irradiation and the other types of external stimuli (pH, temperature, mechanical stress, presence of metal cations and other chemical species, etc.) In this case, a usually colorless cyclic form (Sp) turns into a brightly colored merocyanine (Mc, Scheme 1). Due to this multisensitivity and a drastic change of a number of physicochemical properties (polarity, fluorescence, acidity, and affinity to metal ions) upon transformation from one isomer to another, it became possible to utilize SPPs in molecular electronics, chemosensing, bioimaging, photopharmacology, development of smart materials, and many other cutting-edge areas of science and technology [21,22,23,24,25,26,27,28].

The use of SPPs in various biomedical applications has attracted increasing attention in recent years. It is known that the practical biomedical application of many biologically active substances, including 2ME2 [16], is limited by their low solubility and bioavailability and the difficulty of delivery to a specific target. SPPs can offer an excellent solution to most of these problems due to the significant difference in the dipole moment of their Sp and Mc forms. This effect made it possible to create systems based on SPPs and their conjugates with bioactive molecules for targeted delivery and controlled release of drugs [29,30,31,32,33].

Moreover, SPPs stand out against many other organic photochromes by their ability to undergo isomerization under the influence of a number of external stimuli. One of the most promising except light is the action of acids leading to the formation of colored protonated McH isomers (Scheme 1) [21,22,34,35]. Such acidochromic properties allowed the development of different pH-sensitive materials and sensors based on SPPs [36,37,38,39], including fluorescent markers and probes capable of functioning in biological media [40,41,42,43,44]. In the last case, the pH required for switching can be reached near lysosomes and mitochondria, which have already been visualized and tracked using SPP molecules [40,41,42].

Thus, the introduction of a switchable function in the form of a SPP molecule into the structure of a bioactive compound can endow it with unique capabilities for regulating biological activity under the influence of various types of exposure. Recent studies have shown that both SPPs themselves and their conjugates with biologically active substances can exhibit photoswitchable anticancer and antimicrobial activity [45,46,47], while the introduction of SPP fragments into alpha-lipoic acid molecules leads to the manifestation of new biochemical and signaling antioxidant properties and unprecedented biocompatibility of the hybrid compounds [48,49].

## 2. Results and Discussion

### 2.1. Synthesis

**Scheme 2:**
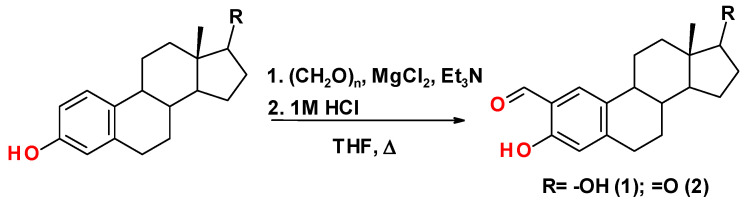
Synthesis of 2-formyl-β-estradiol (**1**) and 2-formylestrone (**2**).

**Scheme 3:**
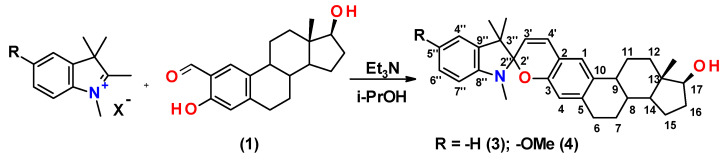
Synthesis of SPPs (**3**–**4**) based on β-estradiol scaffold.

**Scheme 4:**
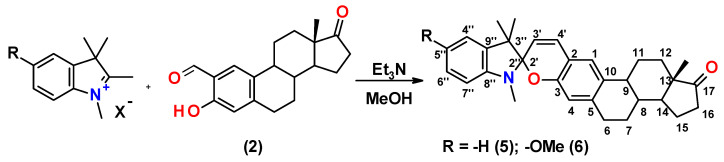
Synthesis of SPPs (**5**–**6**) based on estrone scaffold.

SPPs with estrogen fragments incorporated into the 2*H*-chromene part of the molecule were synthesized by a two-stage procedure. At the first stage, 2-formyl-β-estradiol (**1**) and 2-formylestrone (**2**) were obtained by formylation of the corresponding estrogens under the action of paraformaldehyde in THF in the presence of MgCl_2_ and Et_3_N (Scheme 2) [50,51]. Further, by the cyclocondensation reaction of the obtained aldehydes with the corresponding salts of 1,2,3,3-tetramethyl-3*H*-indolium in the presence of equimolar amounts of Et_3_N upon heating in MeOH or *i*-PrOH, target SPPs (**3**–**6**) were synthesized (Scheme 3 and Scheme 4).

### 2.2. Structure Determination of Estrogen Derivatives

#### 2.2.1. IR and NMR Spectroscopy

The structure of the obtained compounds was confirmed by ^1^H and ^13^C NMR, IR, and HRMS. In the IR spectra of compounds (**3**–**6**), characteristic absorption bands of the main structural fragments are observed. Thus, in the spectra of compounds (**5**–**6**), there is a strong band corresponding to the absorption of the carbonyl group of the estrone fragment in the range of 1738–1739 cm^−1^. The characteristic absorption bands of the C_spiro_–O bond can be found between 940 and 964 cm^−1^, and the absorption bands of the C–N group are located in the ranges of 1241–1245 and 1268–1276 cm^−1^. In addition, in the spectra of estradiol derivatives (**3**–**4**), absorption bands of OH groups are observed in the region of 3314–3435 cm^−1^.

The ^1^H NMR spectra of the obtained compounds demonstrate signals characteristic of all proton-containing groups, which completely correspond to the proposed structures by their chemical shifts, integral intensities and coupling constants.

**Figure 1:**
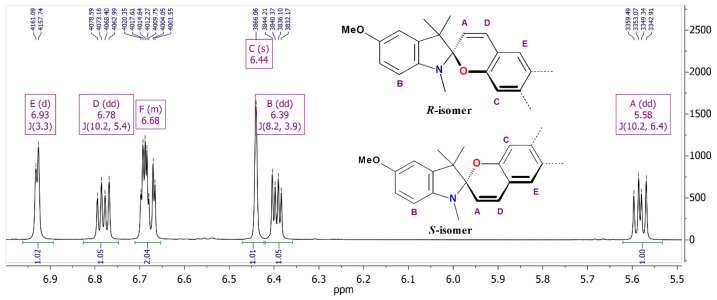
NMR ^1^H spectrum fragment of SPP (**4**) and its stereoisomers with respect to spiro-atom.

It should be noted that most of the proton-containing groups adjacent to the spiro-center of molecules (**3**–**6**) appear in the ^1^H NMR spectra as signals with a “doubled” multiplicity, which is caused by the presence of several stereocenters in molecules of the obtained spirocyclic compounds leading to the appearance of two diastereomers. Figure 1 demonstrates a fragment of the ^1^H NMR spectrum of spiropyran (**4**), which shows the signals of each of the vinyl protons 3′ and 4′ appearing in the form of two couples of doublet signals (A and D, respectively) with equal integral intensity belonging to different diastereomers (*R* and *S* with respect to the spiro-center configuration). The coupling constant *J* = 10.2 Hz corresponds to the *cis*-configuration of this vinyl fragment, which confirms the spirocyclic structure of the obtained compounds. The signals of all 3″-CH_3_ and N-CH_3_ groups in the spectra of SPPs (**3**–**6**) also appear as two singlets with equal intensity in the ranges of 1.13–1.15 and 1.27–1.29 ppm for 3″-CH_3_, and in the region of 2.65–2.79 ppm for the N-CH_3_ signals. The same pattern is observed in the ^13^C NMR spectra, where spiroatoms appear as two low-intensity singlet signals at 103.9–104.5 ppm. A similar phenomenon of diastereomeric signal “splitting” was observed by us earlier for some representatives of the SPP family, for example, unsymmetrical bis-spiropyrans containing two stereocenters in their molecules [52,53].

Proton signals of the aliphatic fragments of β-estradiol and estrone in the spectra of SPPs (**3**–**6**) appear in a wide range of 0.75–3.71 ppm. The chemical shifts of most signals are close to the same signals of aliphatic protons in the corresponding estrogen molecule but are shifted by 0.1–0.2 ppm towards the weak field. The signals of the 13-CH_3_ group protons are located in the strongest field of the spectrum at about 0.75–0.89 ppm. In the weakest region of this spectral range for compounds (**3**–**4**), at 3.71 ppm, the triplet or triplet of doublets signals of the 17-H proton adjacent to the hydroxyl group appear. They are followed by the proton signals of the methylene fragment in position 6 attached to the benzene ring of 2*H*-chromene in the region of 2.69–2.98 ppm. In the spectra of estrone spiropyrans (**5**–**6**), the signals of these protons can be found in the range of 2.75–2.80 ppm. Next to them, at about 2.48 and 2.12 ppm, there is a doublet of doublets signals of methylene protons 16 adjacent to the carbonyl group. In the range of 2.42–2.33 ppm, the signals of one of the methylene group 11 protons, which fall under the deshielding effect of the aromatic core of 2*H*-chromene, are detected.

SPPs (**3**–**5**) were also investigated using two-dimensional NMR techniques, namely COSY ^1^H-^1^H, HSQC ^1^H-^13^C, HMBC ^1^H-^13^C and HMBC ^1^H-^15^N, which made it possible to assign most of the signals in their ^1^H and ^13^C spectra (Figures S1–S28, Supplementary Materials.

#### 2.2.2. Single-Crystal X-ray Analysis

**Figure 2:**
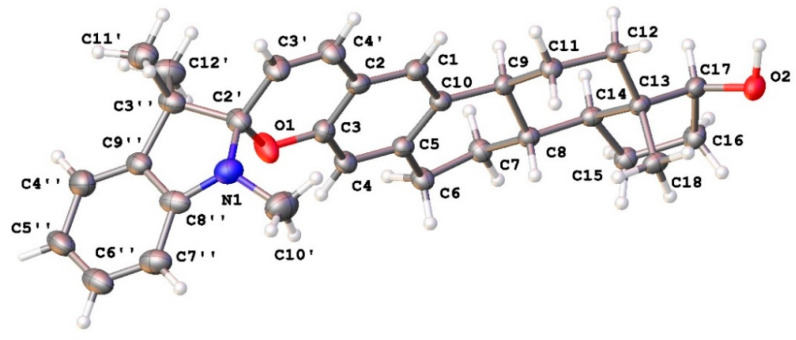
The molecular structure of SPP (**3**) with the atom-numbering scheme. The atoms are represented by thermal vibration ellipsoids of 50% probability. Solvent molecules were omitted for clarity.

Single crystals of (**3**) were grown by slow evaporation of methanol from its solution. The molecular structure of SPP (**3**) was refined by the single-crystal X-ray analysis and presented in Figure 2. In the crystalline state, the benzopyran fragment is almost flat, while only the spiro-C2′-atom deviates from the plane of the indoline fragment by 0.49 Å. The value of the [N1–C2′–O1] angle equal to 107.6(3)° is close to the value expected for the carbon atom in the sp^3^ hybrid state. At the same time, the angle formed by the carbon chain [C3″–C2′–C3′] as a result of steric hindrance from the methyl groups is slightly higher than the idealized value and is 114.0(4)°.

**Figure 3:**
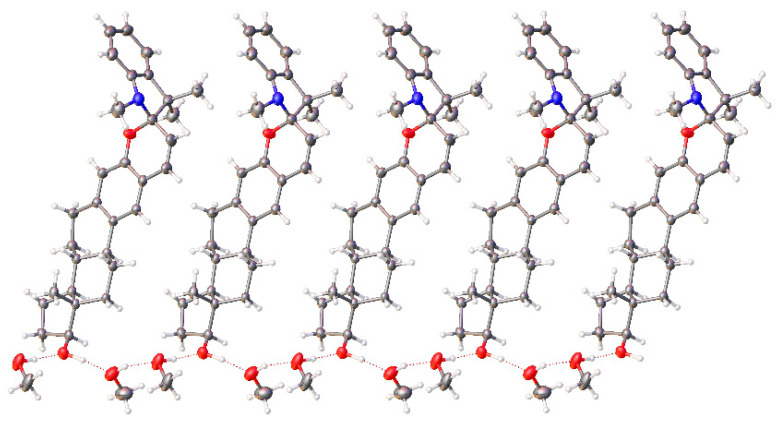
Fragment of the molecular packing in a crystal of SPP (**3**).

The molecular packing of SPP (**3**) is formed by pseudopolymeric ribbons stabilized by intermolecular hydrogen bonds with methanol molecules, which was used as a solvent for growing single crystals (Figure 3).

Using the Mercury software package, 17 short contacts were found in the crystal of compound **3** (Table 1). Obviously, most of them belong to the interactions of spiropyran atoms with atoms of methanol molecules.

#### 2.2.3. Hirshfeld Surface and 2D Fingerprint Plot Analysis

**Figure 4:**
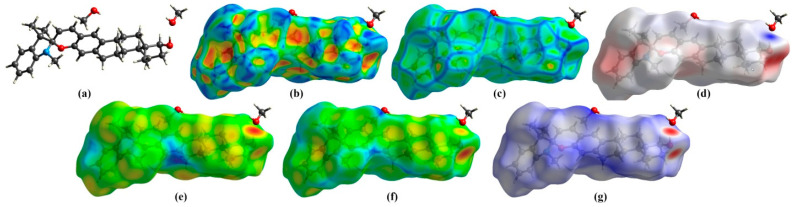
Structure (**a**) and Hirshfeld surfaces of compound **3** mapped with: (**b**) shape index (min. −1 a.u., max. 1 a.u.), (**c**) curvedness (min. −4 a.u., max. 0.4 a.u.), (**d**) electrostatic potential (min. −0.0920 a.u., max. 0.2197 a.u.), (**e**) *d_i_* (min. 0.6718 a.u., max. 2.8826 a.u.), (**f**) *d_e_* (min. 0.6696 a.u., max. 2.5870 a.u.), (**g**) *d_norm_* (min. −0.7096 a.u., max. 1.9416 a.u.).

Figure 4 shows Hirshfeld surfaces (HS) mapped with different characteristics obtained for compound **3**. The HS plotted by the shape index is utilized to check whether *π…π* stacking interaction is present in crystal packing or not [54,55]. Consecutive red and blue triangular regions on the HS indicate the presence of *π…π* stacking interaction. There are no consecutive regions of triangular shape on the HS, indicating that a pronounced π…π stacking interaction is absent in the crystal packing of compound **3** (Figure 4b). The curvedness of HS shows that there are no large flat areas (Figure 4c) outlined in blue, also indicating the absence of *π…π* stacking interaction between molecules in the compound **3** crystal [54,56,57]. These results are consistent with an absence of short contacts C…C in the obtained 2D fingerprint plots (Figure S36 in Supplementary Materials).

The enrichment ratio *E_XY_* of a pair of elements (X,Y) is defined as the ratio between the proportion of actual contacts *C_XY_* in the crystal and the theoretical proportion of random contacts [58]. The enrichment ratio is expected to be generally larger than unity for pairs of elements that have a high propensity to form contacts in crystals, while pairs that tend to avoid contact with each other should yield an *E_XY_* value lower than unity. Analysis of the 2D fingerprint plots showed that H…H (80.7%), O…H (5.8%) and C…H (12.7%) types of interactions dominate in the crystals (Table 2). This fact indicates the predominance of van der Waals interactions in the crystal of compound **3**, as well as the presence of classical and non-classical hydrogen bonds [59]. In Table 2, the values of the enrichment ratio *E_XY_* are also given. It is important to note, that C…H, O…H and N…H interactions are energetically more favorable (*E_XY_* = 1.11, than H…H interactions (*E_XY_* = 0.99).

Potential donors and acceptors of hydrogen in the crystal packing can be classified by the HS plotted by the electrostatic potential (Figure 4d). Hydrogen bond donors and acceptors are represented by the blue and red areas of the HS, respectively. It can be seen from Figure 3d that the pronounced acceptors in the crystal of compound **3** are the oxygen atom and carbon atoms of aromatic systems. Almost all hydrogen atoms can act as hydrogen bond donors. The parameter *d_norm_* is the normalized contact distance; *d_i_* is normalized by the van der Waals radius of the involved atom (Figure 4e); *d_e_* is similarly normalized by the van der Waals radius of the external atom (Figure 4f), and the sum of these two normalized quantities is a property of *d_norm_*. Whether the atoms make intermolecular contacts closer than the sum of their van der Waals radii, these contacts will be highlighted in red on the *d_norm_* surface (Figure 4g). The longer contacts are colored blue, and the contacts around the sum of the van der Waals radii are white [54]. It was established that in the crystal of compound **3**, classical H–O…H hydrogen bonds are formed between the hydroxyl groups of the β-estradiol fragment of SPP and methanol molecules (Figure S37 in Supplementary Materials). As for non-classical hydrogen bonds, only two of them were found in crystals: C(4′)…H(6B) (*l*(A…H) = 2.780 Å, *l*(D…A) = 3.717 Å, angle (D–H…A) is 144.7°) and H(12D)…C(8″) (*l*(A…H) = 2.740 Å, *l*(D…A) = 3.599 Å, angle (D–H…A) is 136.1°).

The crystal cavities were found to take 220.48 Å^3^ (15.01% of the total unit cell volume). It is important to note that this value is relatively high in comparison with the salt (11.43 and 11.97%) [60] and formyl-substituted spiropyrans (10.75 and 12.54%) [61], which we have previously studied. Probably, such a loose packing of molecules in this crystal is associated with a small number of intermolecular interactions due to the small number of functional groups that contribute to the formation of intermolecular hydrogen bonds. The largest part of the molecule of compound **3** is an aliphatic steroid fragment, which does not allow one to observe various types of *π…π* stacking interaction in the crystal. This fact is quite important, as a large number of cavities in the crystal, especially near the photoreaction center, can contribute to the manifestation of photochromism in the crystalline state [62].

### 2.3. UV-Vis Spectral Studies

**Figure 5:**
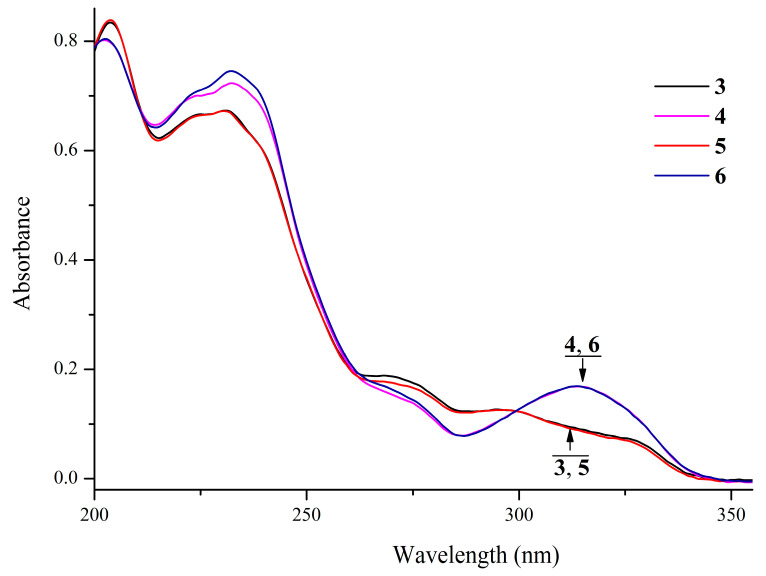
UV–Vis spectra of compounds (**3**–**6**) in acetonitrile (*C* = 2·10^−5^ M, *l* = 1 cm, *T* = 293 K).

The spectral, photo- and acidochromic properties of SPPs (**3**–**6**) were studied in acetonitrile solutions at *T* = 293 K. Under these conditions, all compounds exist in the cyclic form Sp, which is confirmed by the presence of absorption bands exclusively in the UV region. The most intense absorption bands are observed at 202–204 and 231–232 nm with the molar extinction coefficients of 40,100–41,800 and 33,600–37,300 M^−1^·cm^−1^, respectively (Table 3, Figure 5). In this case, the introduction of a methoxy group into the indoline moiety of the molecule leads to the appearance of a pronounced absorption maximum at 314 nm (*ε* = 8400–8500 M^−1^·cm^−1^).

The irradiation of SPP solutions with UV light did not induce the appearance of new absorption bands in the visible region of the spectrum characteristic for the merocyanine form Mc (Scheme 1), which indicates the lack of photochromic properties for studied SPPs at room temperature. This fact can be explained by the absence of electron-withdrawing substituents in the 2*H*-chromene fragment of the studied compounds, which would stabilize the open form of SPP [21]. In addition, no appearance of new absorption bands upon irradiation may be due to the short lifetimes of the Mc forms of SPPs at room temperature.

**Figure 6:**
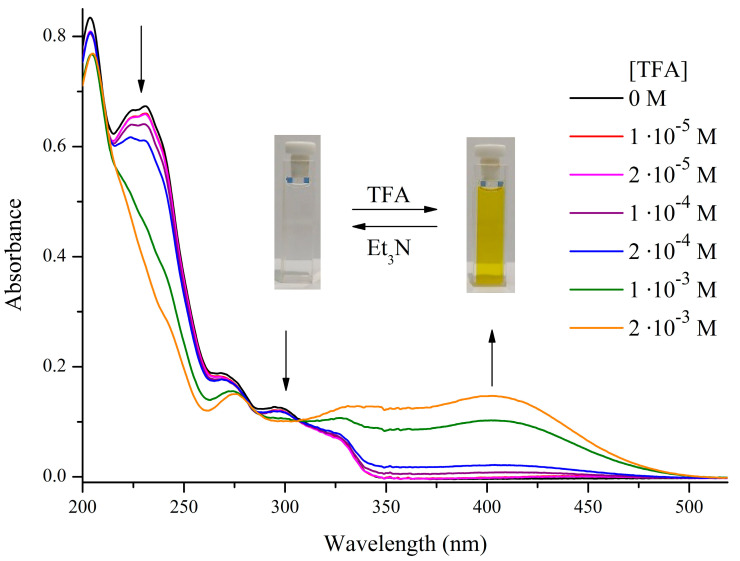
Changes in the UV–Vis spectra of SPP (**3**) in acetonitrile (*C* = 2·10^−5^ M, *l* = 1 cm, *T* = 293 K) under increasing TFA concentration.

**Figure 7:**
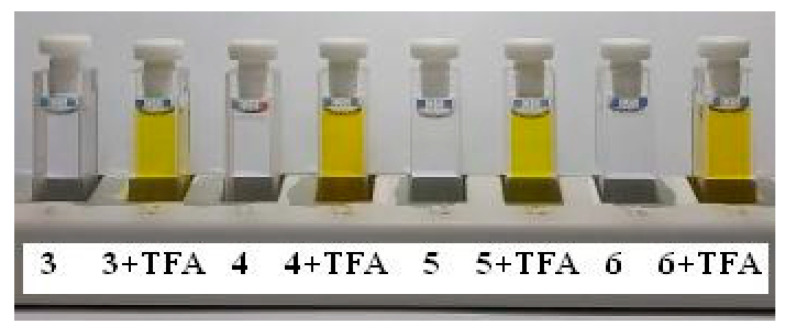
Color changes of acetonitrile solutions of compounds (**3**–**6**) in the presence of TFA at room temperature.

However, all SPPs were found to demonstrate pronounced acidochromic properties. Upon addition of trifluoroacetic acid (TFA), colorless solutions of compounds (**3**–**6**) acquired an intense yellow color due to the transformation of the cyclic Sp form into the protonated merocyanine forms McH (Scheme 1, Figure 6, Figure 7 and Figures S38–S40). In the absorption spectra of the compounds, a broad absorption band characteristic of these isomers appeared in the visible region with a maximum of 397–402 nm. At the same time, the addition of triethylamine to the colored solutions of SPPs (**3**–**6**) led again to discoloration, which indicates the reversibility of this process.

### 2.4. Quantum Chemical Investigations

#### 2.4.1. Isomerization of SPPs in the Ground State

**Scheme 5:**
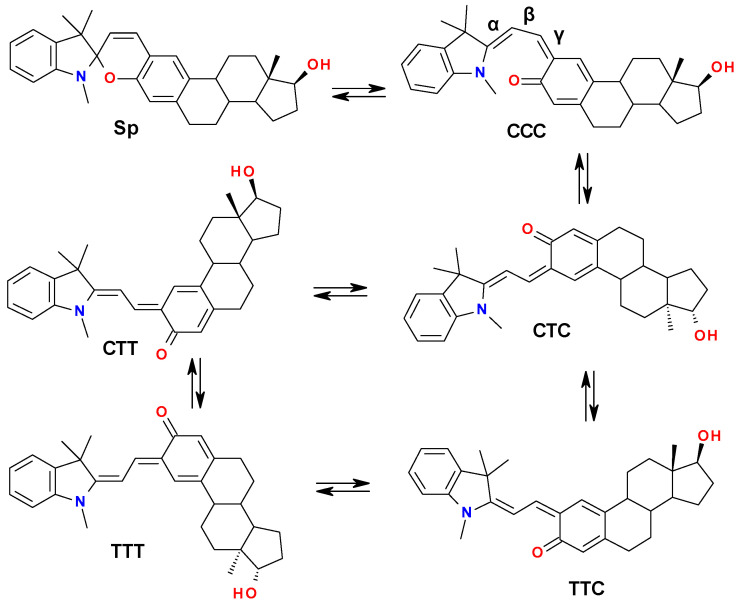
Proposed isomerization mechanism of SPP (**3**).

In order to explain the observed chromogenic properties of the studied spiropyrans, we performed theoretical calculations of the isomerization reactions’ energy profile in the ground state (GS) using compound (**3**) as a representative example. The considered mechanism included closed Sp and the most important conformers of the open Mc form, namely, the completely cisoid CCC form, which forms immediately after the C_spiro_–O bond cleavage, and all possible transoid with respect to the central β-bond conformers connected by the corresponding transition states (TS) (Scheme 5). Previously in a large number of experimental and computational works [63,64,65,66,67,68,69,70], it was shown that the most energetically favorable Mc conformers formed in the course of both thermal and photochemical reactions are TTC and TTT ones.

**Figure 8:**
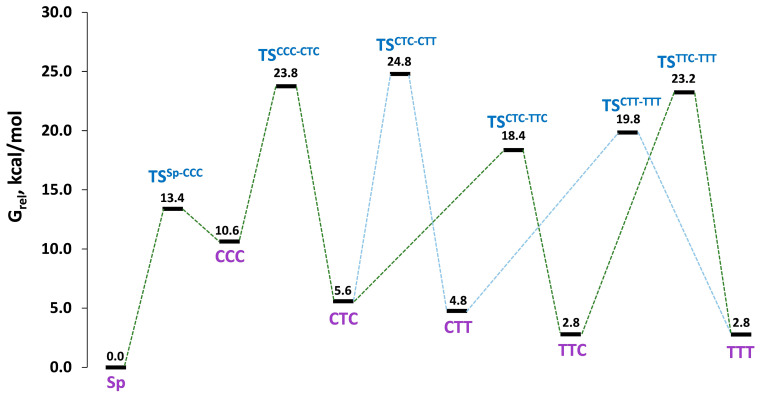
Relative Gibbs free energy (G_rel_, kcal/mol) of spiropyran (**3**) with respect to the closed Sp form during isomerization process in GS according to the data of PBE0-D3/def2-TZVP calculations in acetonitrile solutions.

The results of DFT calculations demonstrated that all Mc forms of compound (**3**) are less thermodynamically stable than the Sp isomer in GS (Table 4, Figure 8). As expected, TTC and TTT isomers were found to be the most energetically favorable among them, with almost equal values of Gibbs free energy (G). The difference in their free energies compared to the closed Sp form (G_rel_) amounted to 2.8 kcal/mol.

The energy barrier of the first stage of isomerization, accompanied by the C_spiro_–O bond breaking, amounted to 13.4 kcal/mol, which is in good agreement with the previously calculated values for unsubstituted indoline SPP [65,66]. However, the energy difference between TS^Sp-CCC^ and CCC was found to be only 2.8 kcal/mol, *i.e.*, the reverse process of ring closure is much more thermodynamically favorable than the ring opening in the GS.

The next considered isomerization stage included rotation around the central β-bond, leading to the formation of the transoid CTC isomer, and was found to be energetically profitable (ΔG = −5.0 kcal/mol). The calculated barrier for this process was equal to 13.2 kcal/mol.

Further rotation around α- and γ-bonds, corresponding to CTC→TTC and TTC→TTT isomerizations, is accompanied by overcoming the energy barriers of 12.8 and 20.4 kcal/mol, respectively. Altering the order of rotations leads to another way from CTC to TTT through the CTT form. In this case, the barriers of rotation around α- and γ-bonds amounted to 15.0 and 19.2 kcal/mol. The intermediate CTT form turned out to be more stable than CTC by 0.8 kcal/mol but destabilized with respect to TTT and TTC conformers by 2.0 kcal/mol. Moreover, despite the equality in energy with the TTT form, TTC can be considered the most favorable of the Mc isomers due to the rather high TTC→TTT isomerization barrier (20.4 kcal/mol). As can be seen, the highest found energy barriers (19.2 and 20.4 kcal/mol) corresponded to the rotations around γ-bond with the formation of CTT and TTT forms exactly. This fact may indirectly evidence an increased γ-bond multiplicity, which indicates a greater contribution of the quinoid Mc forms over the zwitterionic ones.

It can be concluded that the process of thermic isomerization of studied SPP into Mc forms is not observed at ambient conditions even in polar MeCN solution due to a quite high total barrier of 26.8 kcal/mol of the first two stages together with the significant energetic disadvantage of the CCC and CTC isomers compared to the closed Sp form.

#### 2.4.2. Mechanism of Acidochromic Transformations

**Scheme 6:**
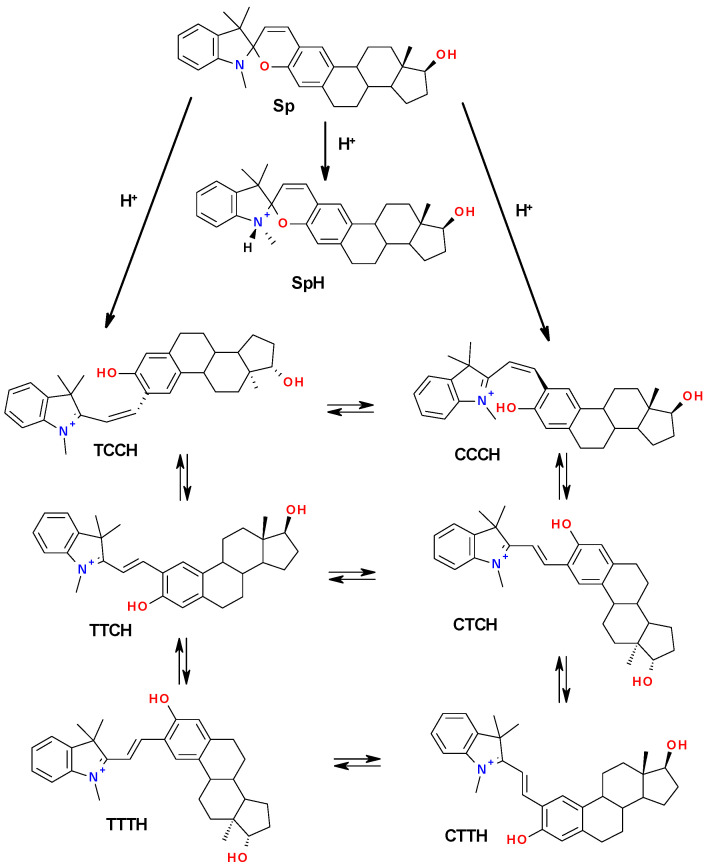
Considered acidochromic transformations of SPP (**3**).

To continue this research, we simulated the process of acidochromic isomerization between the cationic protonated merocyanine forms McH of compound (**3**) (Scheme 6). It should be noted that there are two main proton acceptors in a molecule of SPP—the nitrogen of the indoline and the oxygen of the 2*H*-chromene fragment. In the first case, the protonation leads to the formation of N-protonated closed isomer SpH, while in the second case, it leads to the open cisoid isomers TCCH or CCCH. It should be noted that the TCC-configured conformer was detected only for the protonated McH but not for the Mc form. The other important β-transoid conformers can be obtained consequently by the rotations around β- and α- or γ-bonds.

**Figure 9:**
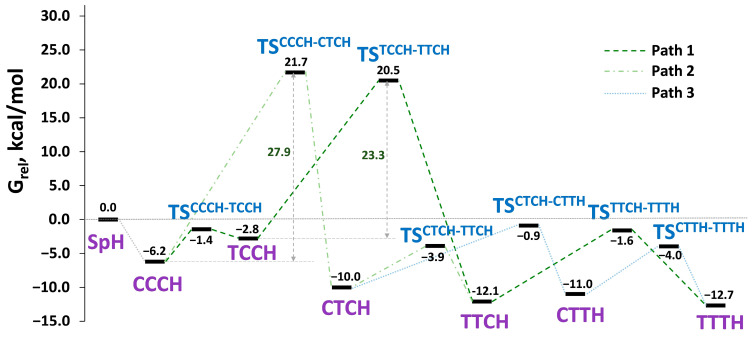
Relative Gibbs free energies (G_rel_, kcal/mol) of the protonated cationic forms of spiropyran (**3**) with respect to SpH isomer during isomerization according to the PBE0-D3/def2-TZVP calculations in MeCN solutions.

The results of DFT calculations (Figure 9, Table 5) have shown that all the open protonated McH forms are lower in energy than the N-protonated closed SpH. The most energetically favorable conformers, as before, were TTCH and TTTH with G_rel_ = −12.1 and −12.7 kcal/mol.

The open cisoid isomers CCCH and TCCH forming after O-protonation of SPP are more stable than N-protonated form by 6.2 and 2.8 kcal/mol. This fact, together with the previously shown barrierless proton transfer from the indoline nitrogen to the 2*H*-chromene oxygen atom [66], indicates the higher probability of forming a CCCH isomer at the first stage of protonation. Even if TCCH forms at first, it can be easily converted to the more stable CCCH due to the very small energy barrier of 1.4 kcal/mol for this process. Further, there are three possible paths on the way to the TTTH form.

The first pathway from CCCH to TTCH (Path 1, Figure 9) involves consequent rotation around α- and β-bonds with the formation of the intermediate TCCH conformer. The second one, with an altered order of rotations, goes through the CTCH form. In both cases, the time-limiting process is the rotation around the β-bond, which needs to overcome a barrier of 23.3 and 27.9 kcal/mol, correspondingly, making Path 1 more probable. The total energy barriers of the first two steps are 28.1 and 34.0 kcal/mol, which also speaks in favor of the reaction proceeding along Path 1. The last step is the same for both Paths 1 and 2. It involves rotation around γ-bond and the formation of TTTH form. The energy barrier of this step is 10.5 kcal/mol.

The third possible Path 3 branches off from Path 2 and CTCH conformer and passes through the CTTH form. In this case, the energy barriers of rotations around γ- and α-bonds were found to be 9.1 and 7.0 kcal/mol, respectively. However, both CTCH and CTTH isomers are destabilized with respect to their α-transoid analogs by 1.7–2.1 kcal/mol. These facts, along with the higher energy barrier on the first steps, make this pathway less probable.

It should be noted that all the processes associated with the change from *cis*- to *trans*-configuration of α-, β- and γ-bonds (except CCCH-TCCH transformation) are energetically favorable. At the same time, the energy barriers of rotation around single α- and γ-bonds in the case of the protonated McH form decrease by about a factor of two compared to similar processes for uncharged Mc forms ranging from 4.8 to 10.5 kcal/mol. As for the barrier of rotation around the double β-bond corresponding to the CCCH→CTCH and TCCH→TTCH transitions, it increases up to 27.9 and 23.3 kcal/mol due to the increase in the bond multiplicity.

To estimate the real energy profit of the SPP (**3**) protonation with trifluoroacetic acid (TFA) and to understand its mechanism, we simulated the process of proton transfer from TFA to the oxygen atom of the 2*H*-chromene part of the molecule. In the first stage, it results in the formation of the β-cisoid structures, which present the ionic pairs of the protonated cations (CCCH or TCCH) and the TFA anions with a hydrogen bond between the carbonyl oxygen of TFA and the hydrogen of the protonated merocyanine OH group (Figure S39). ΔG of the protonation reaction was calculated by Equation (1):

(1) $${∆G}_{prot}^{298}=G\left(McH*TFA\right)-G\left(SP\right)-G\left(TFA\right)$$

and amounted to −2.0 kcal/mol, in the case of CCCH ∗ TFA structure and 2.0 kcal/mol for the TCCH ∗ TFA formation (Table S23). These values indicate the possibility of a spontaneous reaction only with the formation of CCCH ∗ TFA conformer under considered conditions, while the generation of TCCH ∗ TFA form is energetically unprofitable. The further isomerization of the cations to the most stable conformations TTC and TTT gave the additional benefit in Gibbs energy of 4.2 and 4.7 kcal/mol making ΔG = −6.2 and −6.7 kcal/mol, respectively.

Thus, we have comprehensively studied the mechanism of possible acidochromic transformations of SPP (**3**). The data obtained confirms the energetic profit of the formation of O-protonated McH forms starting with CCCH conformer, which can further transform into the more stable TTCH or TTTH forms by the Path 1 depicted in Figure 9 through the intermediate destabilized TCCH isomer.

### 2.5. Determination of the Protonated McH Forms’ Structure

To experimentally establish the structure of the protonated McH forms, we performed additional studies using ^1^H, COSY ^1^H-^1^H and NOESY ^1^H-^1^H NMR spectroscopy for compound (**3**). The corresponding spectra were recorded after the addition of an excess of deuterated TFA to a solution of the compound in a deuteriosolvent. The results of the studies confirm that the mechanism of acidochromic transformation includes two main stages: protonation of the oxygen atom of the 2*H*-chromene moiety with the formation of the cisoid CCCH isomer and *cis-trans* isomerization around the β-bond with the obtaining of a more stable transoid isomer, which is in good agreement with the results of the DFT calculations. In the ^1^H NMR spectrum of a freshly prepared solution of SPP (**3**) with an excess of TFA-*d*, the characteristic signals of the charged 3*H*-indolium fragment are observed, namely, two three-proton singlets from 3″-Me groups with very close chemical shifts at 1.62 ppm and a three-proton singlet signal of the N^+^-Me group at 3.35 ppm (Figure S29).

**Figure 10:**
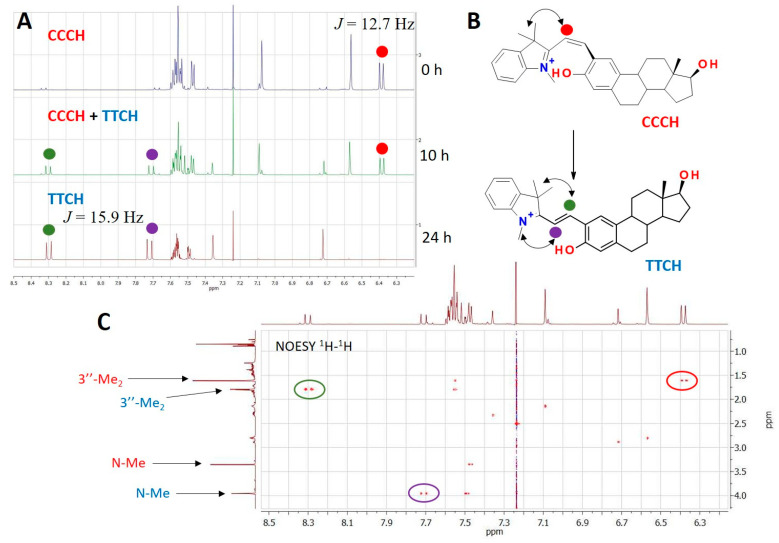
Stack of the ^1^H NMR spectra fragments of protonated SPP (**3**) solution recorded at different times (**A**); isomerization scheme (**B**); fragment of the NOESY ^1^H-^1^H spectrum for a mixture of CCCH and TTCH isomers (**C**). The correlations of the most important signals are highlighted with circles of corresponding color.

The signals of the 3′ and 4′ protons of vinyl fragments are shifted downfield with respect to the analogous signals of the Sp form and appear as two doublets at 6.39 and 7.53 ppm with *J* = 12.7 Hz. This value is intermediate for the *J*-constants of the same protons belonging to the cyclic Sp and the *trans*-McH forms. It is characteristic of the protonated cisoid isomer CCCH [66,71]. During the recording of 2D spectra, requiring a prolonged time of signal accumulation, it was found that the second set of the downfield-shifted signals, characteristic of *trans*-McH isomers, appeared and became dominant in the spectra with time (Figure 10A). This fact indicates the occurrence of a *cis-trans* isomerization reaction in solution (Figure 10B). In particular, the signals of 3″-Me groups are shifted to 1.80, and N^+^-Me-group to 3.96 ppm. The 3′ and 4′ signals of the vinyl fragment protons appear as two doublets at 7.72 and 8.30 ppm with *J* = 15.9 Hz, characteristic of the *trans*-configuration. The corresponding correlations for vinyl protons of both forms presented in the solution were observed in the COSY ^1^H-^1^H spectrum (Figure S30).

NOESY ^1^H-^1^H spectrum (Figure 10C), registered for a mixture of isomers, provided additional evidence in favor of different configurations of the central bonds system for the observed conformers. Thus, the presence of a cross peak between the signals of the 3′-H and 3″-Me proton groups, together with the absence of correlations in the aliphatic region for the 4′ proton of the initially present in the solution form, confirms its CCCH configuration. As for the transoid McH isomer, which is accumulated over time, its NOESY spectrum contains cross-peaks of its 3′ and 4′ protons signal with the signals of the N^+^-Me and 3″-Me groups, respectively. These facts, along with the absence of the NOE effect between the protons 3′ and 1, confirm its TTCH configuration. It should be noted that after 24 h, TTCH became the only form observed in the solution according to the ^1^H NMR data (Figure S31), which indicates that the *cis-trans* isomerization reaction proceeded completely.

These experimental data confirm the results of our DFT calculations and an assumption about the realization of Path 1 during the acidochromic isomerization of studied SPP with the formation of the CCCH isomer at the first stage. The mechanism proposed by us can be expanded on other spiropyrans and can alleviate further studies of their acidochromic properties.

### 2.6. Cytotoxicity Study of the Spirocyclic and 2-Formyl Derivatives of β-Estradiol and Estrone

One of the main aims of this study was to investigate the short-term cytotoxicity of the new spirocyclic derivatives of β-estradiol and estrone, as well as their precursors—2-formyl-β-estradiol (**1**) and 2-formylestrone (**2**). HeLa cells and two dosing regimens (50 and 200 μM) were used in a 24-h cytotoxicity test corresponding to one cell cycle. The results of the test are provided in Table 6 and Table 7.

The results showed that the original 2-formyl derivatives of estradiol (**1**) and estrone (**2**) did not possess any pronounced toxicity to HeLa cells at the final concentration of 50 μM after 24 h of incubation. However, the higher concentration of 200 μM of both substances caused acute toxicity. IC_50_ was nearly achieved for (**1**), and 42% of cell death was caused by the aldehyde (**2**) (*p* = 0.001).

The spirocyclic derivatives of these two compounds exhibited different and non-additive properties with respect to their components. SPPs of the estradiol series (**3**–**4**) with the hetarene moieties of the molecules identical to those of estrone derivatives (**5**–**6**) were distinctly toxic already at 50 μM. SPP (**3**) with the unsubstituted hetarene fragment caused the death of 27% of the cells, and methoxy-substituted SPP (**4**)—of 15%. At the higher concentration, their toxicity almost tripled. Additionally, the toxicity of SPP (**3**) exceeded that of 2-formyl-β-estradiol (**1**), causing the death of about 68% of cells, while the toxicity of SPP (**4**) was approximately at the same level with it (39% dead cells for the SPP (**4**) vs. 41% for the compound (**1**)).

On the contrary, SPPs of the estrone series (**5**–**6**), as well as their precursor (**2**), did not manifest pronounced toxicity to HeLa cells at the concentration of 50 μM and exposure for 24 h. However, their effects were somewhat different at the increased concentration of 200 μM. The unmodified spiropyran (**5**) caused the death of approximately 25% of the cells under these conditions, while its 5-methoxy derivative (**6**) remained non-toxic, in contrast to 2-formylestrone, which caused the death of approximately 50% of the cells.

Therefore, although the hetarene moieties of SPPs of estradiol (**3**–**4**) and estrone (**5**–**6**) series coincide in pairs, the conjugation of the indoline heterocycle through the spiro-carbon atom with the estrogen fragment leads to the formation of compounds with non-additive characteristics. One of the most interesting features identified was the elimination of toxicity for the estrone-based SPP (**6**) at the final concentration of 200 μM (although the same concentration for the original 2-formylestrone (**2**) almost coincided with the IC_50_), and pronounced toxicity increase of the estradiol-based SPP (**3**) in comparison with its analogs.

## 3. Materials and Methods

All reagents were purchased from Alfa Aesar and Merck and were used as received. Organic solvents used were purified and dried according to standard methods.

NMR ^1^H and ^13^C spectra were recorded on a Bruker AVANCE-600 (600 MHz) spectrometer at the Center for Collective Use “Molecular Spectroscopy” of Southern Federal University. The signals were assigned relative to the signals of residual protons of the deuteriosolvent CDCl_3_ (δ = 7.26 ppm). It should be noted that the signals of two observed diastereomers of target SPPs were hardly distinguishable, so they were combined and represented as signals with a “doubled” multiplicity (e.g., “d” and “dd” instead of expected “s” and “d”) to simplify the ^1^H NMR spectra descriptions.

IR spectra of the compounds were recorded on a Jasco FT/IR-6800 IR Fourier-transform spectrometer (Jasco Inc., Easton, MD, USA) with an ATR PRO ONE attenuated total reflectance attachment.

High-resolution mass spectra were registered with a Bruker Maxis spectrometer (electrospray ionization in MeCN solution, using HCO_2_Na−HCO_2_H for calibration).

Electronic absorption spectra of the investigated compounds were recorded on an Agilent–8453 spectrophotometer equipped with a thermostatic cell. The irradiation of solutions with the filtered light of a high-pressure Hg lamp was performed on Newport 66902 equipment. UV/Vis spectra were recorded using standard 1 cm quartz cells. Acetonitrile of the spectroscopic grade (Aldrich) was used to prepare solutions.

Melting points were determined on a Fisher-Johns apparatus (Thermo Fisher Scientific Inc., Waltham, MA, USA).

### 3.1. Synthetic Procedures and Spectral Data

*2-Formyl-β-estradiol* (**1**)**.** This compound was obtained by the method based on the previously described one [50,51]. Briefly, in a 100 m round-bottom flask, 1.122 g (4 mmol) of β-estradiol and 0.48 g (16 mmol) of paraformaldehyde were dissolved in 25 mL of anhydrous THF. Then, 1.142 g (12 mmol) of MgCl_2_ and 1.7 mL of triethylamine were added to the solution. The mixture was refluxed by stirring under argon for 4 h. Next, 30 mL of 1M HCl was added, and the reaction mixture was stirred for 30 min. After that, it was poured into 100 mL of water and extracted with ethyl acetate. The extract was kept over anhydrous Na_2_SO_4_ overnight. Then, the solvent was distilled off, and the residue was purified by column chromatography on silica gel using dichloromethane (DCM) as an eluent. The product was crystallized from MeOH to form a white powder. **Yield** 0.716 g (69.2%). **m.p.** 220 °C. The NMR data obtained are consistent with what was described in the literature [51].

*2-Formylestrone* (**2**)**.** This compound was obtained from estrone according to the method described for compound (**1**). **Yield** 0.334 g (56%). **m.p.** 163–164 °C. The NMR data obtained are consistent with the described in the literature [51].

*17β-Hydroxy-1″,3″,3″-trimethylspiro[estra-1,3,5(10)-triene-[3,2-e]-2H-pyran-2′,2″-indoline]* (**3**)**.** 0.274 g (0.001 mol) of 1,2,3,3-tetramethyl-3*H*-indolium perchlorate was added to a boiling solution of 0.300 g (0.001 mol) of 2-formyl-β-estradiol (**1**) in 15 mL of isopropyl alcohol. After then, 0.1 mL of piperidine was added dropwise with stirring. The reaction mixture was refluxed for 2 h; then, the solvent was evaporated to half the volume. The residue was poured into 30 mL of water and extracted with DCM. The extract was dried over anhydrous Na_2_SO_4_, the solvent was evaporated, and the residue was purified by column chromatography on silica gel using DCM as an eluent. The product was recrystallized from a mixture of acetonitrile and hexane (1:1). **Yield** 0.121 g (26.6%). **m.p.** 145 °C. **IR**, ν, cm^−1^: 3314 (O–H); 1607, 1639 (C=C); 1241, 1276 (C–N); 940, 959 (C_spiro_–O). **NMR ^1^H** (CDCl_3_), *δ*, ppm (*J,* Hz): 7.17 (1H, td, *J* = 7.7, 1.2, 6″-H), 7.07 (1H, ddd, *J* = 7.3, 3.6, 1.1, 4″-H), 6.96 (1H, d, *J* = 3.3, 4-H), 6.82 (2H, m, 4′-H + 5″-H), 6.52 (1H, dd, *J* = 7.7, 4.3, 7″-H), 6.46 (1H, s, 1-H), 5.61 (1H, dd, *J* = 10.2, 6.2, 3′-H), 3.73 (1H, t, *J* = 8.5, 17-H), 2.77–2.66 (m, 5H, N-CH_3_ + 2 × 6-H), 2.32 (1H, m, 11-H), 2.12 (2H, m, 9-H + 16-H), 1.97 (1H, dt, *J* = 12.7, 3.4 Hz, 12-H), 1.84 (1H, ddt, *J* = 12.7, 5.8, 2.8, 7-H), 1.69 (2H, m, 15-H), 1.50 (2H, m, 11-H + 16-H), 1.41 (2H, m, 8-H + 17-OH), 1.36 (1H, dd, *J* = 12.2, 5.8, 15-H), 1.32 (3H, d, *J* = 6.4, 3″-CH_3_), 1.28 (2H, m, 12-H + 7-H), 1.20 (1H, m, 14-H), 1.17 (3H, d, *J* = 7.1, 3″-CH_3_), 0.79 (3H, s, 13-CH_3_). **NMR ^13^C** (CDCl_3_), *δ*, ppm: 152.46 (C-3), 148.49 (C-8″), 138.88 (C-5), 137.09 (C-9″), 132.17 (C-10), 129.69 (C-4′), 127.63 (C-6″), 123.59 (C-1), 121.58 (C-4″), 119.10 (C-5″), 118.53 (C-3′), 116.61 (C-2), 114.75 (C-4), 106.86 (C-7″), 104.23 (C-2′), 82.06 (C-17), 51.72 (C-3″), 50.27 (C-14), 44.01 (C-9), 43.41 (C-13), 38.94 (C-8), 36.89 (C-12), 30.79 (C-16), 29.80 (C-6), 29.08 (N-CH_3_), 27.34 (C-7), 26.49 (C-11), 26.02 (3″-CH_3_), 23.27 (C-15), 20.39 (3″-CH_3_), 11.20 (13-CH_3_). **HRMS** (**ESI**)**:** *m*/*z* [M + H]^+^ calculated for C_31_H_38_NO_2_: 456.2897; found: 456.2889.

*17β-Hydroxy-5″-methoxy-1″,3″,3″-trimethylspiro[estra-1,3,5(10)-triene-[3,2-e]-2H-pyran-2′,2″-indoline]* (**4**)**.** 0.331 g (0.001 mol) of 1,2,3,3-tetramethyl-5-methoxy-3*H*-indolium iodide was added to a boiling solution of 0.300 g (0.001 mol) of 2-formyl-β-estradiol (**1**) in 15 mL of isopropyl alcohol. Afterward, 0.1 mL of piperidine was added dropwise with stirring. The reaction mixture was refluxed for 4 h; then, the solvent was evaporated to half the volume. The residue was poured into 30 mL of water and extracted with DCM. The extract was dried over anhydrous Na_2_SO_4_, the solvent was evaporated, and the residue was purified by column chromatography on silica gel using DCM as an eluent. The product was recrystallized from a mixture of acetonitrile and hexane (1:1). **Yield** 0.137 g (28.2%). **m.p.** 189 °C. **IR**, ν, cm^−1^: 3435 (O–H); 1602, 1616, 1643 (C=C); 1243, 1271 (C–N); 958 (C_spiro_–O). **NMR ^1^H** (CDCl_3_), *δ*, ppm (*J,* Hz): 6.93 (1H, d, *J* = 3.3, 4-H), 6.78 (1H, dd, *J* = 10.2, 5.4, 4′-H), 6.71–6.65 (2H, m, 4″-H + 6″-H), 6.44 (1H, s, 1-H), 6.39 (1H, dd, *J* = 8.2, 3.9, 7″-H), 5.58 (1H, dd, *J* = 10.2, 6.4, 3′-H), 3.77 (3H, s, O-CH_3_), 3.71 (1H, td, *J* = 8.0, 3.3, 17-H), 2.79–2.69 (2H, m, 6-H), 2.66 (3H, d, *J* = 14.5, N-CH_3_), 2.33–2.25 (1H, m, 11-H_a_), 2.17–2.04 (2H, m, 9-H + 16-H), 1.94 (1H, dt, *J* = 12.6, 2.9, 12-H), 1.85–1.77 (m, 1H, 7-H), 1.67 (1H, ddd, *J* = 12.3, 11.2, 3.2, 15-H), 1.50 (1H, td, *J* = 12.1, 3.0, 11-H), 1.44–1.47 (1H, d, *J* = 8.4, 16-H), 1.41–1.36 (2H, m, 8-H + 17-OH), 1.33 (1H, dd, *J* = 12.2, 5.8, 15-H), 1.30–1.24 (6H, m, 3″-CH_3_ + 7-H + 12-H), 1.20–1.11 (4H, m, 3″-CH_3_ + 14-H), 0.76 (3H, s, 13-CH_3_). **NMR ^13^C** (CDCl_3_), *δ*, ppm: 153.69 (C-5″), 152.35 (C-3), 142.70 (C-8″), 138.69 (C-5), 138.57 (C-9″), 131.95 (C-10), 129.46 (C-4′), 123.43 (C-4), 118.35 (C-3′), 116.47 (C-2), 114.57 (C-1), 111.21 (C-6″), 109.58 (C-4″), 106.81 (C-7″), 104.46 (C-2′), 81.90 (C-17), 55.95 (OCH_3_), 51.74 (C-3″), 50.10 (C-14), 43.84 (C-9), 43.24 (C-13), 38.78 (C-8), 36.71 (C-12), 30.63 (C-16), 29.65 (C-6), 29.34 (N-CH_3_), 27.18 (C-7), 26.32 (C-11), 25.75 (3″-CH_3_), 23.11 (C-15), 20.14 (3″-CH_3_), 11.04 (13-CH_3_). **HRMS** (**ESI**)**:** *m*/*z* [M + H]^+^ calculated for C_32_H_40_NO_3_: 486.3003; found: 486.2987.

*1″,3″,3″-Trimethylspiro[estra-1,3,5(10)-triene-[3,2-e]-2H-pyran-2′,2″-indoline]-17-one* (**5**)**.** 0.274 g (0.001 mol) of 1,2,3,3-tetramethyl-3*H*-indolium perchlorate was added to a boiling solution of 0.298 g (0.001 mol) of 2-formylestrone (**2**) in 20 mL of MeOH. The reaction mixture was refluxed with stirring for 2 h. After cooling to r.t., the precipitate was filtered, washed with MeOH and purified by column chromatography on silica gel using chloroform as an eluent. **Yield** 0.105 g (23%). **m.p.** 147–149 °C. **IR**, ν, cm^−1^: 1738 (C=O), 1608, 1646 (C=C), 1245, 1272 (C-N), 963, 949 (C_spiro_-O). **NMR ^1^H** (CDCl_3_), *δ*, ppm (*J*, Hz): 7.15 (1H, td, *J* = 7.6, 0.7, 6″-H), 7.04 (1H, д, *J* = 7.3, 4″-H), 6.94 (1H, д, *J* = 3.0, 4-H), 6.85–6.77 (2H, m, 5″-H, 4′-H), 6.50 (1H, dd, *J* = 7.7, 3.2, 7″-H), 6.45 (1H, s, 1-H), 5.61 (1H, dd, *J* = 10.2, 6.3, 3′-H), 2.78 (2H, dd, *J* = 12.2, 7.2, 6-H), 2.71 (3H, d, *J* = 13.8, N-CH_3_), 2.48 (1H, dd, *J* = 19.1, 8.6, 16-H), 2.42–2.35 (1H, m, 11-H), 2.20 (1H, dd, *J* = 16.1, 6.2, 9-H), 2.16–2.07 (1H, m, 16-H), 2.05–1.99 (1H, m, 15-H), 1.97–1.91 (2H, m, 7-H, 12-H), 1.64–1.46 (5H, m, 14-H, 8-H, 15-H, 11-H, 12-H), 1.40–1.35 (1H, m, 7-H), 1.29 (3H, d, *J* = 5.7, 3″-CH_3_), 1.15 (3H, d, *J* = 7.0, 3″-CH_3_), 0.89 (3H, d, *J* = 1.0, 13-CH_3_). **NMR ^13^C** (CDCl_3_), *δ*, ppm: 220.75, 152.45, 148.30, 138.43, 136.89, 131.39, 129.44, 127.49, 123.44, 121.43, 118.97, 118.56, 116.60, 114.64, 106.72, 104.12, 51.59, 50.47, 47.97, 43.89, 38.31, 35.84, 31.60, 29.50, 28.92, 26.48, 25.94, 25.77, 21.56, 20.21, 13.85. **HRMS** (**ESI**)**:** *m*/*z* [M + H]^+^ calculated for C_31_H_36_NO_2_: 454.2741; found: 454.2747.

*5″-Methoxy-1″,3″,3″-trimethylspiro[estra-1,3,5(10)-triene-[3,2-e]-2H-pyran-2′,2″-indoline]-17-one* (**6**)**.** 0.331 g (0.001 mol) of 1,2,3,3-tetramethyl-5-methoxy-3*H*-indolium iodide was added to a boiling solution of 0.298 g (0.001 mol) of 2-formylestrone (**2**) in 20 mL of MeOH. The reaction mixture was refluxed with stirring for 2 h. After cooling to r.t., the precipitate was filtered, washed with MeOH and purified by column chromatography on silica gel using chloroform as an eluent. **Yield** 0.121 g (25%). **m.p.** 145–147 °C. **IR**, ν, cm^−1^: 1738 (C=O), 1605, 1644 (C=C), 1246, 1272 (C-N), 964, 948 (C_spiro_-O). **NMR ^1^H** (CDCl_3_), *δ*, ppm (*J,* Hz): 6.92 (1H, s, 4-H), 6.78 (1H, dd, *J* = 10.2, 2.9, 4′-H), 6.71–6.64 (2H, m, 4″-H, 6″-H), 6.45 (1H, s, 1-H), 6.39 (1H, d, *J* = 7.7, 7″-H), 5.59 (1H, dd, *J* = 10.2, 3.4, 3′-H), 3.76 (3H, s, O-CH_3_), 2.82–2.70 (2H, m, 6-H), 2.65 (3H, d, *J* = 7.3, N-CH_3_), 2.48 (1H, dd, *J* = 18.6, 8.4, 16-H), 2.35 (1H, s, 11-H), 2.20–1.89 (5H, m, 16-H, 15-H, 7-H, 9-H, 12-H), 1.50 (6H, m, 14-H, 8-H, 15-H, 7-H, 11-H, 12-H), 1.26 (3H, d, *J* = 3.0, 3″-CH_3_), 1.13 (3H, d, *J* = 3.5, 3″-CH_3_), 0.88 (3H, s, 13-CH_3_). **NMR ^13^C** (CDCl_3_), *δ*, ppm: 220.78, 153.81, 152.60, 142.75, 138.61, 138.49, 131.42, 129.45, 123.50, 118.63, 116.72, 114.69, 111.32, 109.82 − 109.55, 106.88, 104.60, 56.03, 51.85, 50.57, 48.04, 43.97, 38.40, 35.91, 31.69, 29.56, 29.39, 26.56, 26.01, 25.82, 21.64, 20.20, 13.93. **HRMS** (**ESI**)**:** *m*/*z* [M + H]^+^ calculated for C_32_H_38_NO_3_: 484.2846; found: 484.2844.

### 3.2. Single-Crystal X-ray Analysis Methods

Single crystals of SPP (**3**) were grown from saturated MeOH solution. The X-ray diffraction data sets were recorded on an Agilent SuperNova diffractometer (Agilent Technologies, Santa Clara, CA, USA) using a microfocus X-ray radiation source with copper anode and Atlas S2 two-dimensional CCD detector. The reflections were recorded, and unit cell parameters were determined and refined using the dedicated CrysAlisPro software suite [72]. Using Olex2 [73], the structure was solved with the ShelXT program [74] and refined with the ShelXL program [75]. Crystal Data for C_33_H_45_NO_4_ (*M* =519.70 g/mol): monoclinic, space group P2_1_ (no. 4), *a* = 6.60840(10) Å, *b* = 11.1387(2) Å, *c* = 19.9797(4) Å, *β* = 92.557(2)°, *V* = 1469.22(5) Å^3^, *Z* = 2, *T* = 100.00(10) K, μ(Cu Kα) = 0.597 mm^−1^, *Dcalc* = 1.175 g/cm^3^, 39,389 reflections measured (4.428° ≤ 2Θ ≤ 152.858°), 6141 unique (*R*_int_ = 0.0493, R_sigma_ = 0.0254) which were used in all calculations. The final *R*_1_ was 0.0623 (I > 2σ(I)), and *wR*_2_ was 0.1736 (all data). Short contacts were found using the Mercury software package [76].

Full information about the structure and the CIF file was deposited with the Cambridge Crystallographic Data Center (CCDC 2163146) and is available free of charge upon request at www.ccdc.cam.ac.uk/data_request/cif (accessed on 1 May 2022).

### 3.3. Hirshfeld Surface Analysis

Intermolecular interactions of SPP (**3**) were defined by Hirshfeld surfaces (HSs) [77] and 2D fingerprint plots [78], which were successfully acquired from CrystalExplorer (v21.5) [79] using the CIF-files of SCXRD data. HSs and 2D fingerprint plots were calculated by the MP2 method using the 6-311G(d,p) basis.

### 3.4. Computational Methods

All theoretical calculations were carried out by using Orca 5.0.3 quantum chemistry program package [80,81]. The optimized geometries of the compounds were obtained at the DFT level with the PBE0 [82,83] hybrid functional and the def2-TZVP basis set [84] considering the atom-pairwise dispersion correction with the Becke–Johnson damping scheme (D3BJ) [85,86]. The conductor-like polarizable continuum model (CPCM) was used to account for the effects of acetonitrile solution [87]. For each of the stationary points and transition states, the matrix of constants has been calculated analytically in order to confirm its nature. Appropriate guesses for the TS optimizations were taken from relaxed surface geometry scans along the corresponding coordinate (bond distance or dihedral angle). To save computational time, the RIJCOSX approximation [88,89] and def2/J as the auxiliary basis set [90] were used in all geometry optimizations and vibrational frequency calculations.

### 3.5. Cytotoxicity Assay

We used HeLa cells (kindly provided by the Southern Scientific Centre of the Russian Academy of Science) as the model for in vitro cytotoxicity testing.

The cells were seeded in 24-well plates (SPL Life Sciences, Pocheon-si, South Korea) in GlutaMax DMEM (Thermo Fisher Scientific Inc., Waltham, MA, USA) supplemented with 10% fetal bovine serum (GE Healthcare, Chalfont St Giles, UK) and 50 U/mL penicillin, 50 µg/mL streptomycin (PanEco, Moscow, Russia). The cells were kept at 37°C and 5% CO_2_ under passive humidification in a Sanyo 180-MC incubator.

For the assays, the spiropyrans (**3**–**6**) and the respective 2-formylestrogens (**1**–**2**) were dissolved in DMSO. Two final concentrations were used, 50 and 200 µM. The first concentration was selected as one of the most effectively used in other studies on different models. The second one was four-fold higher to test for acute toxicity. In the lower concentration group, the final DMSO concentration was 0.2% (*v*/*v*). In the second group, it reached 0.8% (*v*/*v*). Due to different DMSO content in the lower and the higher concentrations testing, there were two independent control groups. The cells were treated for 24 h, and the data were collected from three independent experiments performed on different days.

Cell viability was analyzed using the trypan blue exclusion assay over three different days: three biological replicates per day, with two technical replicates per plate well. Briefly, the cells treated with the substances and vehicle were detached with 0.25% trypsin–sodium ethylenediamine tetraacetate solution (PanEco, Moscow, Russia) and mixed with an equal volume of 0.4% trypan blue (Thermo Fisher Scientific Inc., Waltham, MA, USA) and, after 2 min incubation, analyzed with the Countess II FL cell viability analyzer (Thermo Fisher Scientific Inc., Waltham, MA, USA).

Statistical calculations were performed using JASP. To diminish false-positive testing results, statistical differences were assessed using the nonparametric Mann–Whitney criterion.

## 4. Conclusions

A series of novel indoline SPPs with conjugated fragments of human hormones β-estradiol and estrone were synthesized. The structure of the compounds obtained was confirmed by ^1^H and ^13^C NMR, IR and HRMS. The molecular structure of SPP (**3**) was refined by single-crystal X-ray analysis. The study of its crystal packing features using the CrystalExplorer software showed that the resulting single crystal of compound (**3**) is characterized by a large number of voids (15.01%), which can potentially promote the possibility of solid-state photochromism manifestation.

The obtained SPPs lacked photochromic activity at room temperature but possessed pronounced acidochromic properties. The spectra of their protonated McH forms obtained after the addition of TFA to the acetonitrile solutions demonstrated the absorption bands in the visible region with maxima at 397–402 nm, which disappeared after the addition of Et_3_N. The mechanisms of thermal isomerization processes for neutral Sp/Mc and their protonated McH forms were comprehensively studied by DFT methods. The most probable full path of acid-induced isomerization of indoline SPP was proposed for the first time. The calculations revealed an energetic advantage of the O-protonation process with the formation of the CCCH conformer at the first stage and its further transformation to the β-*transoid* isomers. Using 2D NMR methods, such as COSY and NOESY ^1^H-^1^H, the structure of the McH formed in solution was investigated, which enabled the detection of the intermediate CCCH isomer and its conversion into the more stable TTCH. These results are in good agreement with DFT calculations confirming that acidochromic transformation involves two key stages.

The short-term cytotoxicity of the new spirocyclic derivatives of estrogens and their precursors—2-formyl-β-estradiol and 2-formylestrone—was studied on HeLa cells. It was shown that the conjugation of the indoline heterocycle through the spiro-carbon atom with the estrogen fragment leads to the formation of compounds with non-additive characteristics. The most interesting features revealed were the elimination of toxicity of the estrone-based SPP (**6**) at the final concentration of 200 μM (although the same concentration of the original 2-formylestrone (**2**) almost coincided with its IC_50_) and strong toxicity induction for the estradiol-based SPP (**3**) in comparison with its analogs.

Based on the revealed cytotoxicity pattern and stimuli-responsive properties, the synthesized compounds may be used in various cancer-fighting strategies. Thus, acutely toxic SPPs of the estradiol series (3–4) can be used for highly efficient cell killing in situ or in the conditions of targeted delivery. SPP (6), on the contrary, may turn out to be a systemically non-toxic signal-active compound with prolonged effects, which also requires further cellular physiological studies involving molecular genetics and flow cytometry. Such agents may find use in strategies for normalizing the signaling profiles of malignant cells. Currently, we are conducting experiments to evaluate the possibility of increasing or reducing the toxicity of these compounds under different external stimuli.

## Figures and Tables

**Table 1:** Short contacts for compound **3** in the crystalline state.

Short Contacts	l, Å	l − VdW, Å
O(2)…O(1S)	2.678	−0.362
O(2)…O(2S)	2.675	−0.365
O(1S)…O(2S)	2.664	−0.376
C(4′)…H(6B)	2.857	−0.043
H(12D)…C(8″)	2.814	−0.086
H(2)…O(1S)	1.663	1.057
H(2)…H(1S)	2.307	−0.093
H(2)…C(1S)	2.506	−0.394
H(2)…H(1SB)	2.386	−0.014
H(7B)…O(2S)	2.647	−0.073
O(2)…H(2S)	1.707	−1.013
C(17)…H(2S)	2.879	−0.021
H(2)…H(2S)	2.356	−0.044
H(1S)…O(2S)	1.852	−0.868
H(1S)…C(3S)	2.865	−0.035
H(1S)…H(2S)	2.347	−0.053
C(1S)…O(2S)	3.195	−0.025

**Table 2:** Short reciprocal contacts ratio *C_xy_* of actual short contacts between chemical elements in the crystal of SPP **3** and its enrichment ratio *E_XY_*.

Contact	*C_xy_ (E_xy_)*
O…H	0.058 (1.11)
N…H	0.008 (1.11)
C…H	0.127 (1.11)
H…H	0.807 (0.99)

**Table 3:** Spectral properties of the compounds (**3**–**6**) in acetonitrile, *T* = 293 K.

No.	Structure	Form	Absorption *λ*_max_, nm (*ε ·* 10^−3^, M^−1^·cm^−1^)
(**3**)	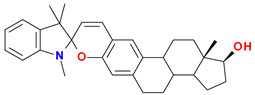	Sp	204 (41.8), 231 (33.7), 268 (9.5), 296 (6.4), 323 ^sh^ (3.8)
		McH	402
(**4**)	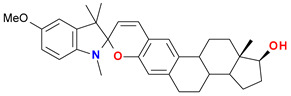	Sp	202 (40.1), 232 (36.2), 269 ^sh^ (7.9), 314 (8.5)
		McH	402
(**5**)	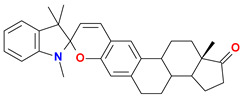	Sp	204 (42.0); 231 (33.6); 268 (8.9); 296 (6.3); 323 ^sh^ (3.7)
		McH	400
(**6**)	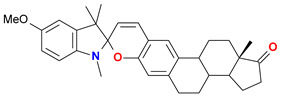	Sp	202 (40.2); 232 (37.3); 269 ^sh^ (8.4); 314 (8.4)
		McH	397

^sh^—shoulder.

**Table 4:** Gibbs free energies (G, a.u.) of the most important isomeric forms and corresponding transition states of compound (**3**), relative free energies (G_rel_, kcal/mol) with respect to the closed Sp form and energy barriers (kcal/mol) of corresponding transformations according to the PBE0-D3/def2-TZVP calculations in acetonitrile solution.

Isomer	G, a.u.	G_rel_, kcal/mol	Energy Barrier, kcal/mol
Sp	−1407.19203162	0	-
TS^Sp-CCC^	−1407.17068088	13.4	13.4
CCC	−1407.17508024	10.6	-
TS^CCC-CTC^	−1407.15417442	23.8	13.2
CTC	−1407.18312876	5.6	-
TS^CTC-CTT^	−1407.15251755	24.8	19.2
CTT	−1407.18443424	4.8	-
TS^CTC-TTC^	−1407.16277247	18.4	12.8
TTC	−1407.18757602	2.8	-
TS^CTT-TTT^	−1407.16040986	19.8	15.0
TS^TTC-TTT^	−1407.15498830	23.2	20.4
TTT	−1407.18760296	2.8	-

**Table 5:** Gibbs free energies (G, a.u.) of the most important protonated forms of compound (**3**) and corresponding transition states, relative free energies (G_rel_, kcal/mol) with respect to SpH isomer and energy barriers (kcal/mol) of corresponding transformations according to the PBE0-D3/def2-TZVP calculations in MeCN solutions.

Isomer	G, a.u.	G_rel_, kcal/mol	Energy Barrier, kcal/mol
SpH	−1407.61996533	0	-
CCCH	−1407.62985427	−6.2	-
TS^CCCH-TCCH^	−1407.62225265	−1.4	4.8
TCCH	−1407.62441867	−2.8	-
TS^CCCH-CTCH^	−1407.58540033	21.7	27.9
CTCH	−1407.63590909	−10.0	-
TS^TCCH-TTCH^	−1407,58729335	20.5	23.3
TS^CTCH-TTCH^	−1407.62617824	−3.9	6.1
TTCH	−1407.63924105	−12.1	-
TS^CTCH-CTTH^	−1407.62138572	−0.9	9.1
CTTH	−1407.63745967	−11.0	-
TS^TTCH-TTTH^	−1407.62253176	−1.6	10.5
TS^CTTH-TTTH^	−1407.62627058	−4.0	7.0
TTTH	−1407.64015436	−12.7	-

**Table 6:** Trypan blue exclusion assay test results for the test compounds at 50 μM.

Group	Viability, %		pMW *	Percent Change *
	Med [25…75] *	M ± SD *		
DMSO (control)	99.5 [96.75…100]	97.88 ± 3.24	-	-
(**1**)	97 [96…98.5]	97.41 ± 1.78	0.296	Non-significant
(**2**)	97 [97…98.25]	97.5 ± 1.679	0.272	Non-significant
(**3**)	72.5 [67.75…99]	72 ± 7.568	0.001	27.1% decrease
(**4**)	85 [83…87.25]	85.08 ± 4.23	0.001	14.6% decrease
(**5**)	96.5 [94.5…99]	96.17 ± 3.51	0.139	Non-significant
(**6**)	97 [93.75…99]	96.5 ± 3.06	0.193	Non-significant

*—Med [25…75]—median value and 25th and 75th percentiles; M ± SD—mean value and standard deviation; pMW—p-level based on Mann–Whitney criterion; Percent change (of viability)—relative to the control group (0.2% DMSO).

**Table 7:** Trypan blue exclusion assay test results for the test compounds at 200 μM.

Group	Viability, %		pMW	Percent Change *
	Med [25…75]	M ± SD		
DMSO (control)	99.5 [96.75…100]	97.88 ± 3.24	-	-
(**1**)	58 [50.5…70.75]	59.75 ± 16.88	0.001	41.7% decrease
(**2**)	50.5 [45.5…56]	50.92 ± 9.99	0.001	49.3% decrease
(**3**)	32 [29…39]	32.33 ± 10.75	0.001	67.8% decrease
(**4**)	60.5 [47…65]	57.42 ± 10.99	0.001	39.2% decrease
(**5**)	73.5 [43.75…98]	69.83 ± 30.36	0.007	26.1% decrease
(**6**)	98 [96.75…99]	97.5 ± 2.15	0.215	Non-significant

*—relative to the control group (0.8% DMSO).

## Data Availability

The data presented in this study are available in the article and Supplementary Material.

## Supplementary Materials

The following supporting information can be downloaded at: https://www.mdpi.com/article/10.3390/molecules28093866/s1, NMR ^1^H, ^13^C, COSY ^1^H-^1^H, HSQC ^1^H-^13^C, HMBC ^1^H-^13^C and HMBC ^1^H-^15^N spectra (Figures S1–S33); HRMS spectra (Figures S34–S37); SC-XRD, Hirshfeld surface and 2D fingerprint plot analysis data (Tables S1–S4, Figures S38–S39); UV–Vis spectra (Figures S40–S42) and DFT calculations data (Tables S5–S31).
