# Supplementary material for: Novel Indoline Spiropyrans Based on Human Hormones β-Estradiol and Estrone: Synthesis, Structure, Chromogenic and Cytotoxic Properties
Source: Molecules. 2023 May 4;28(9):3866. doi: 10.3390/molecules28093866 (PMC10179760; doi:10.3390/molecules28093866)
Supplement: Supplementary file 1 [file molecules-28-03866-s001.zip › molecules-2358291-supplementary.pdf]

Supplementary Materials for the article:

Novel Indoline Spiropyrans Based on Human Hormones β-Estradiol and Estrone: Synthesis, Structure, Chromogenic and Cytotoxic Properties

Ilya V. Ozhogin [1,*], Artem D. Pugachev [1], Nadezhda I. Makarova [1], Anna A. Belanova [2], Anastasia S. Kozlenko [1], Irina A. Rostovtseva [1], Peter V. Zolotukhin [2], Oleg P. Demidov [3], Islam M. El-Sewify [4], Gennady S. Borodkin [1], Anatoly V. Metelitsa [1] and Boris S. Lukyanov [1]

[1] Institute of Physical and Organic Chemistry, Southern Federal University, 194/2 Stachka Ave., 344090 Rostov-on-Don, Russia

[2] Academy of Biology and Biotechnology, Southern Federal University, 194/1 Stachka Ave., 344090 Rostov-on-Don, Russia

[3] Faculty of Chemistry and Pharmacy, North-Caucasus Federal University, 1 Pushkina Str., 355000 Stavropol, Russia

[4] Department of Chemistry, Faculty of Science, Ain Shams University, Cairo 11566, Egypt

[*] Correspondence: iozhogin@sfedu.ru

Table of contents

1. NMR spectra 2 – 18

2. HRMS spectra 19

3. Data of SC-XRD, Hirshfeld surface and 2D fingerprint plot analysis 20 – 24

4. Spectral characteristics and acidochromic properties 24 – 25

5. DFT calculations data 26 – 52

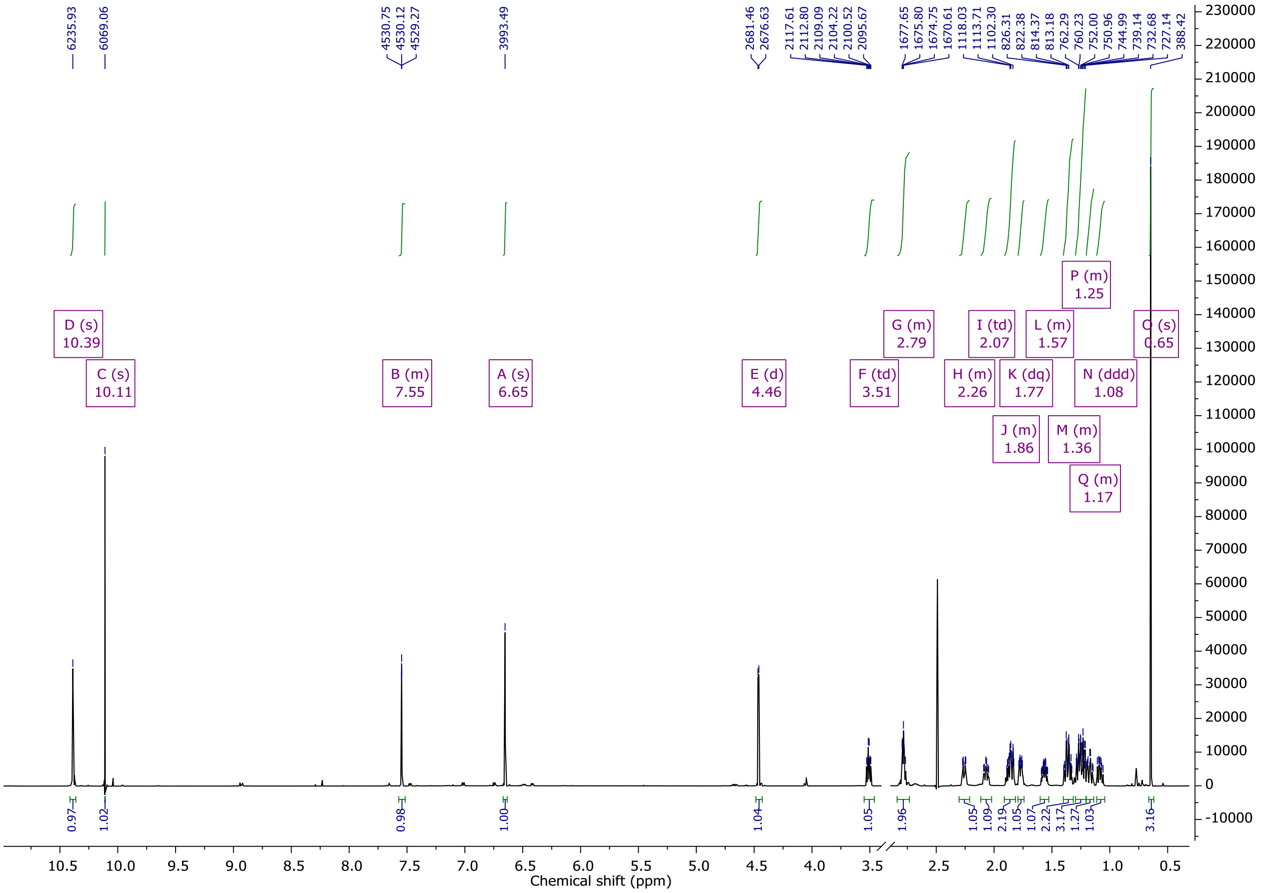

Figure S1. NMR ^{1}H spectrum of 2-formyl-β-estradiol (**1**).

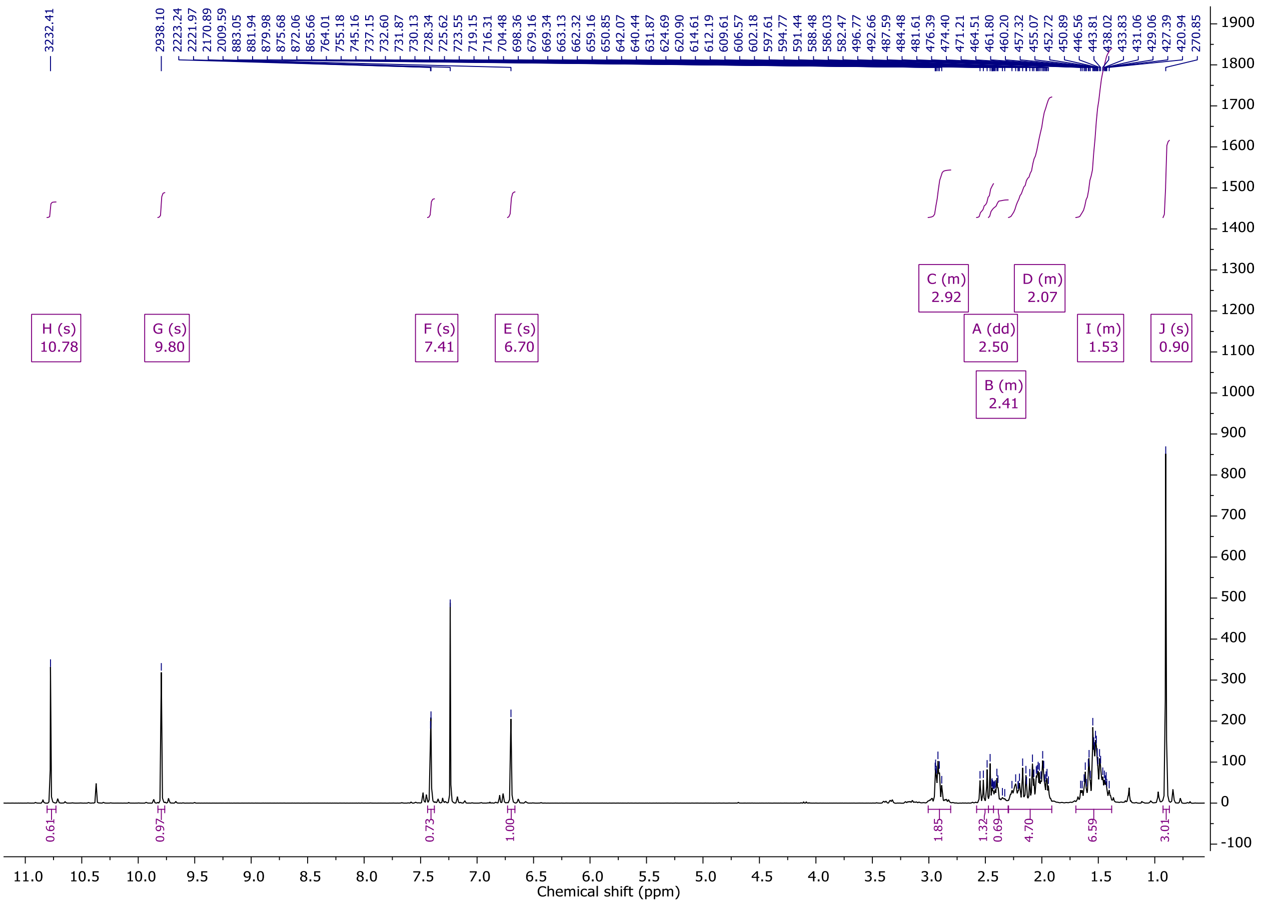

Figure S2. NMR ^{1}H spectrum of 2-formylestrone (**2**).

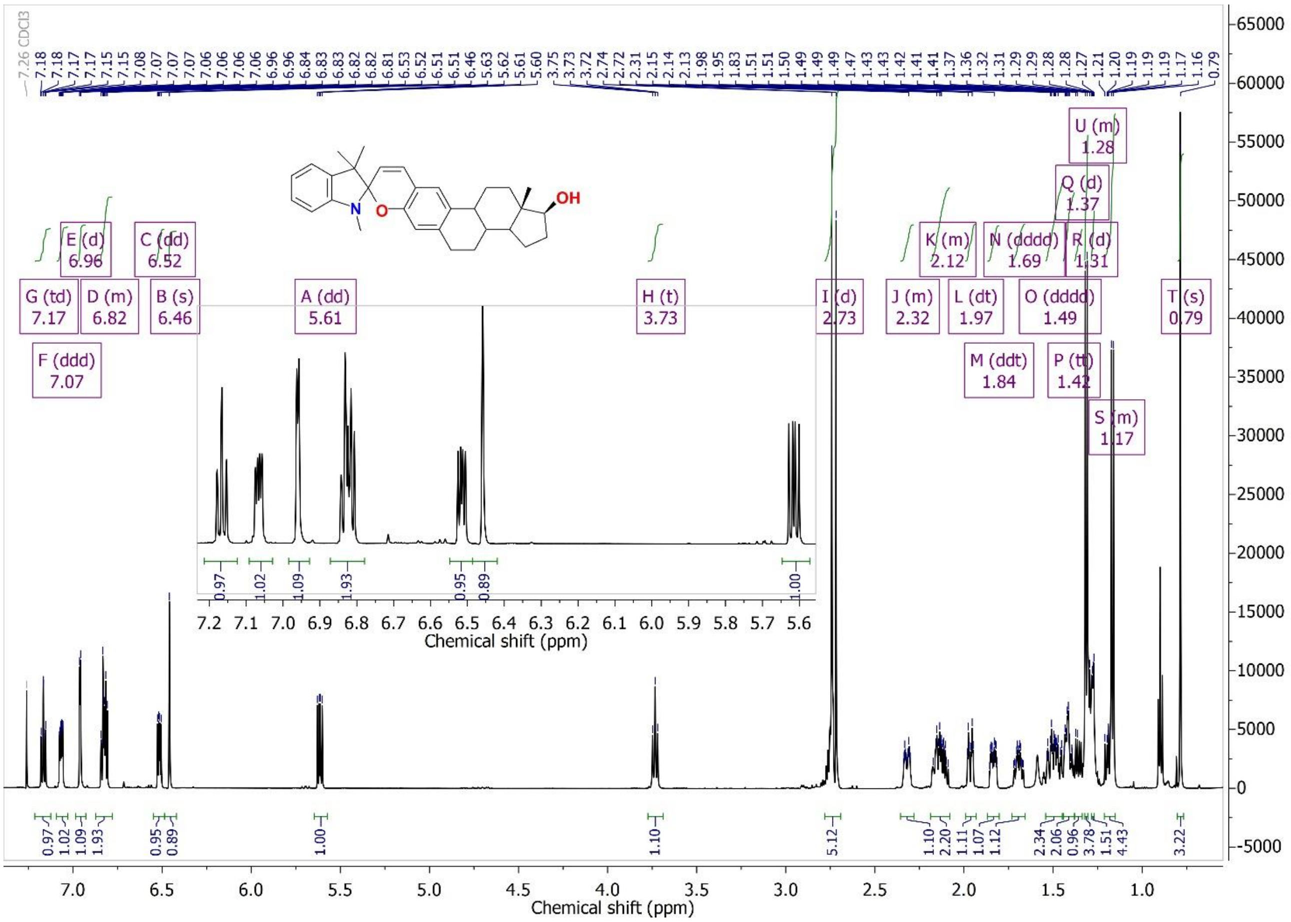

Figure S3. NMR ^{1}H spectrum of SPP (**3**)

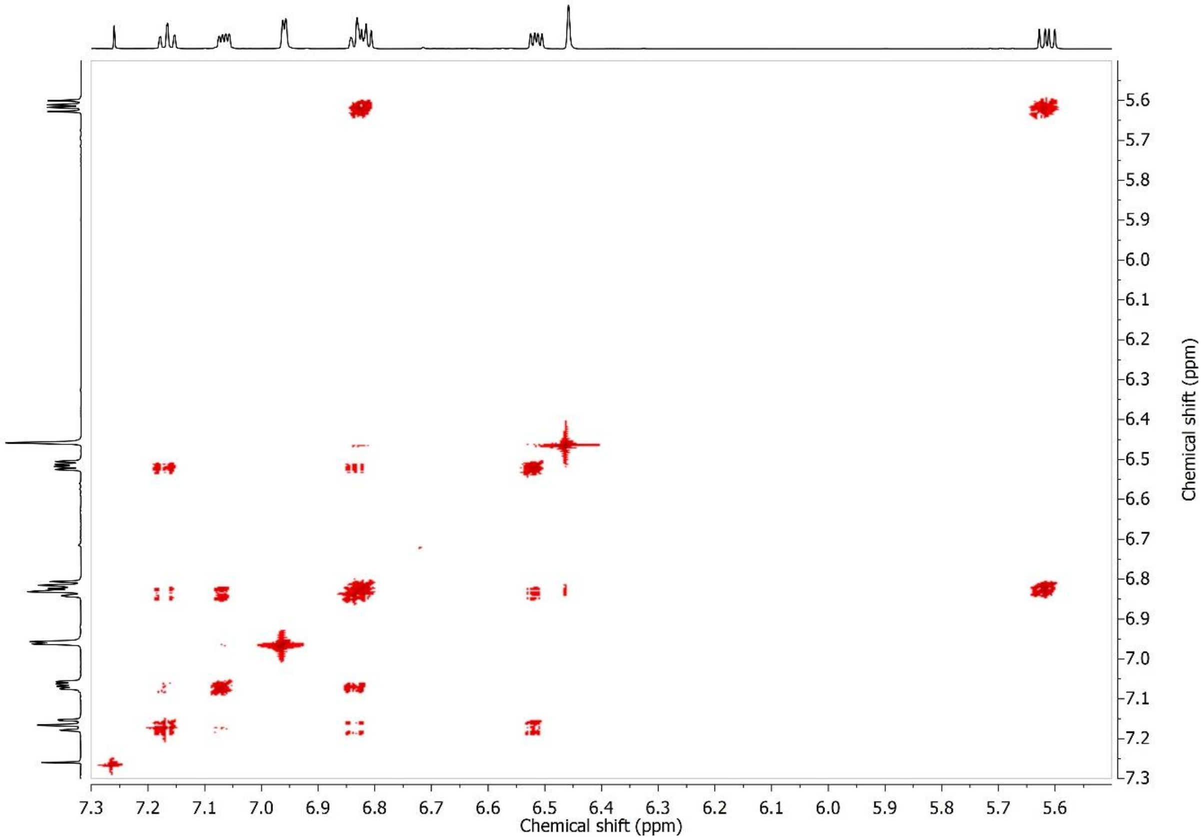

Figure S4. Fragment of COSY ^{1}H-^{1}H NMR spectrum of SPP (**3**), aromatic protons region.

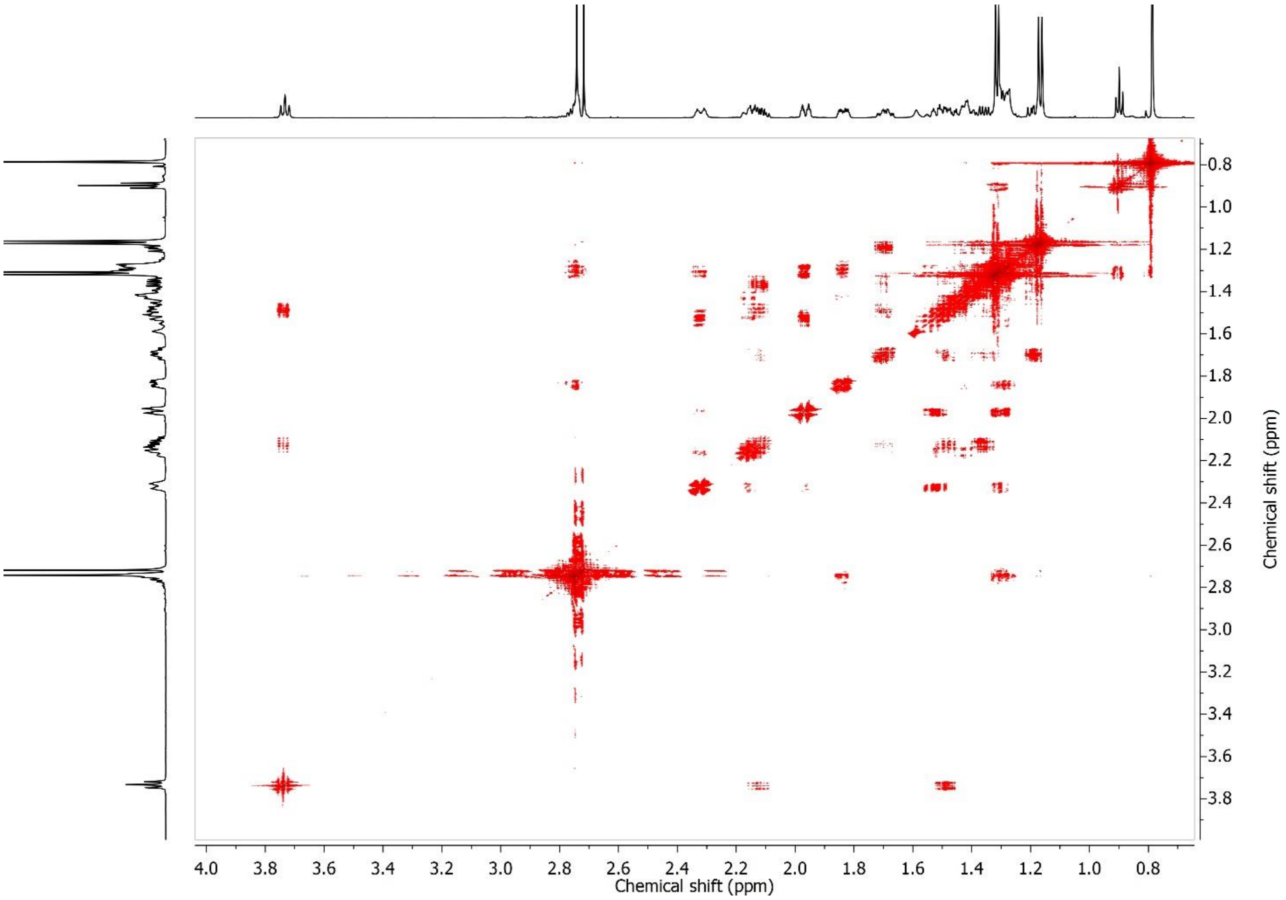

Figure S5. Fragment of COSY ^{1}H-^{1}H NMR spectrum of SPP (**3**), aliphatic protons region.

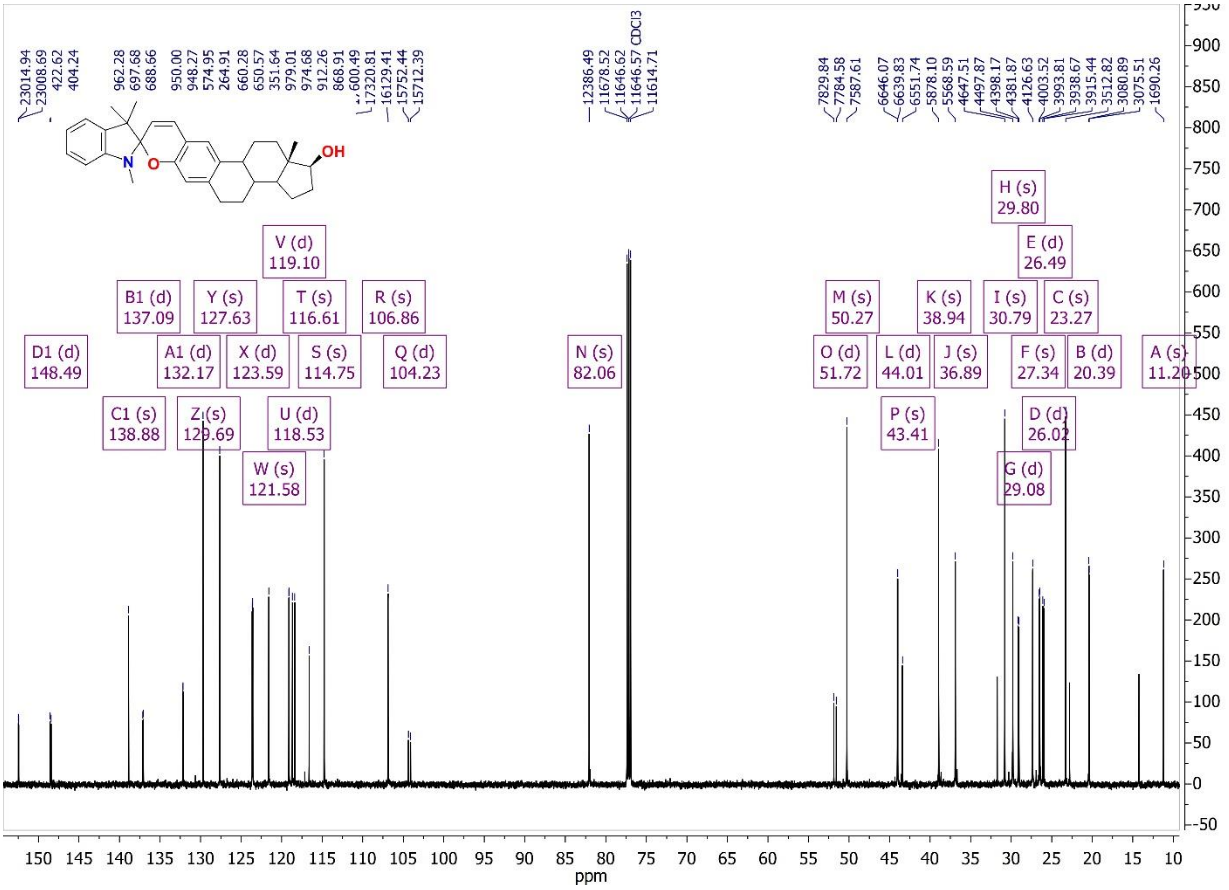

Figure S6. NMR ^{13}C spectrum of SPP (**3**)

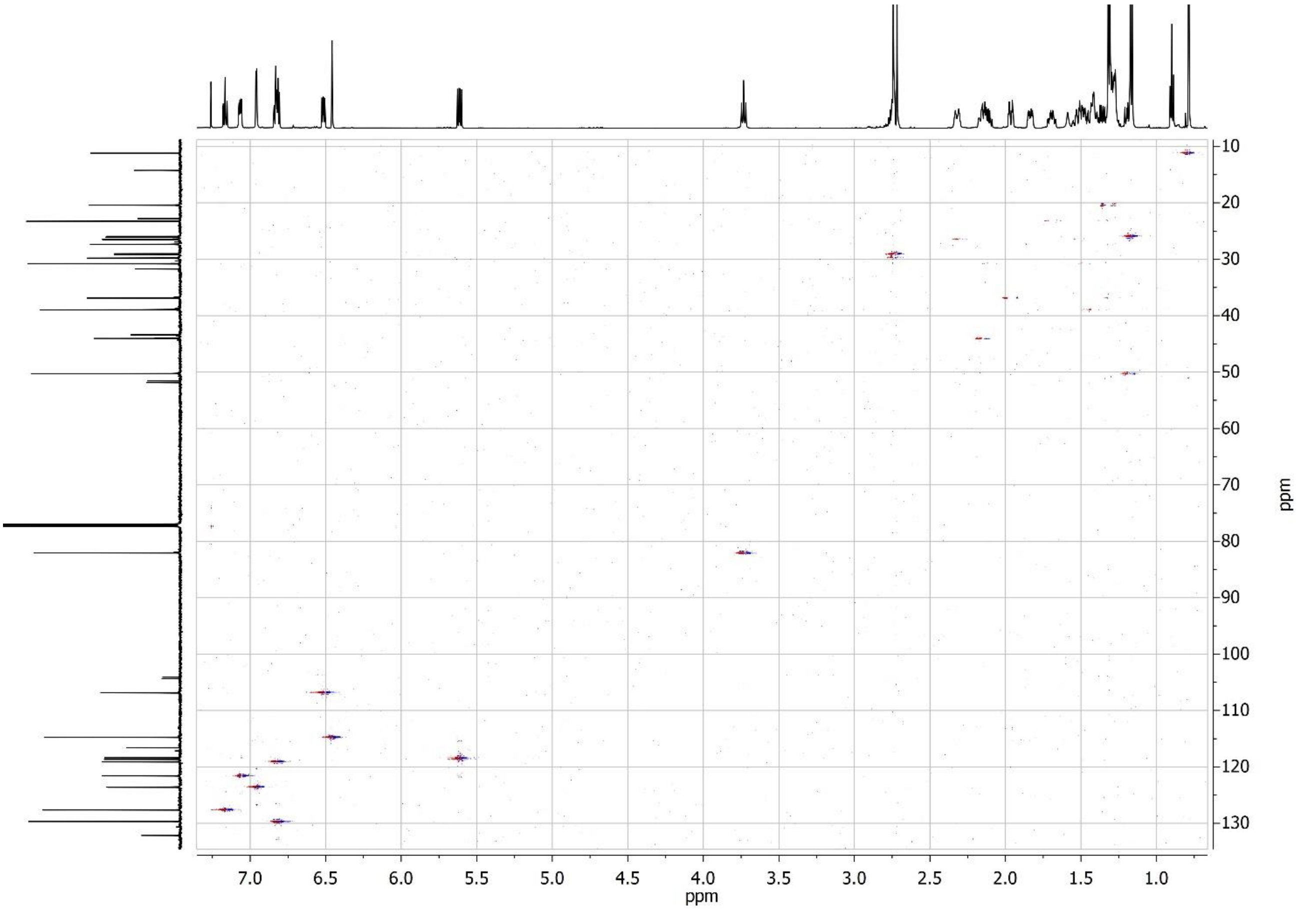

Figure S7. HSQC ^{1}H-^{13}C NMR spectrum of SPP (**3**).

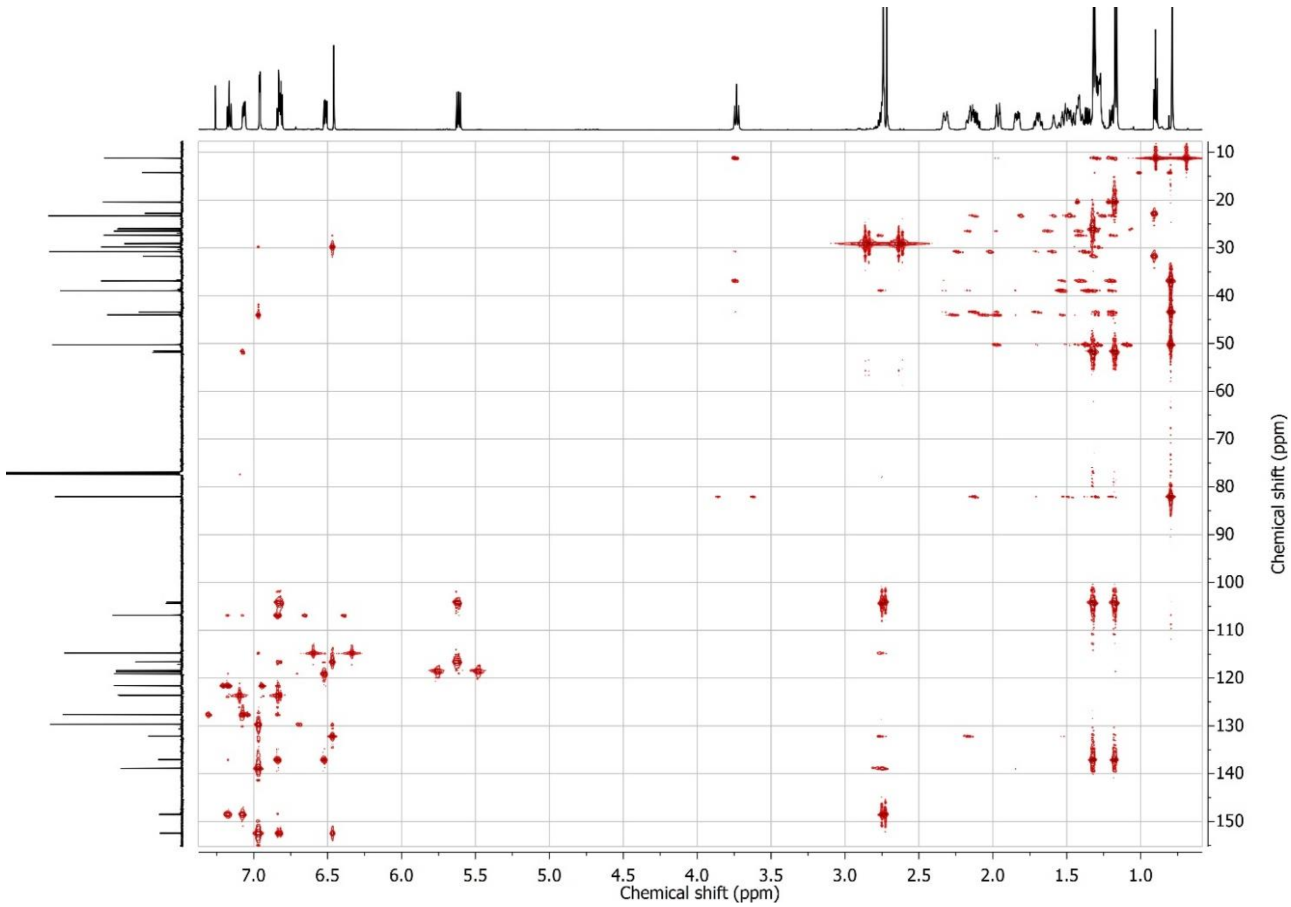

Figure S8. HMBC ^{1}H-^{13}C NMR spectrum of SPP

(**3**).

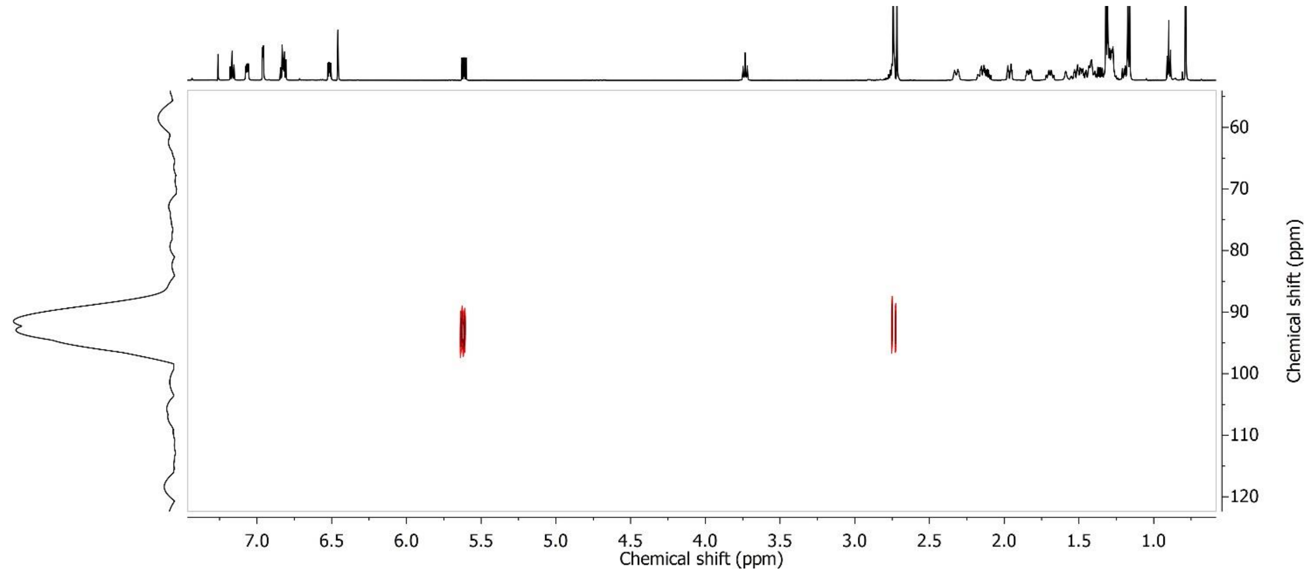

Figure S9. HMBC ^{1}H-^{15}N NMR spectrum of SPP (**3**).

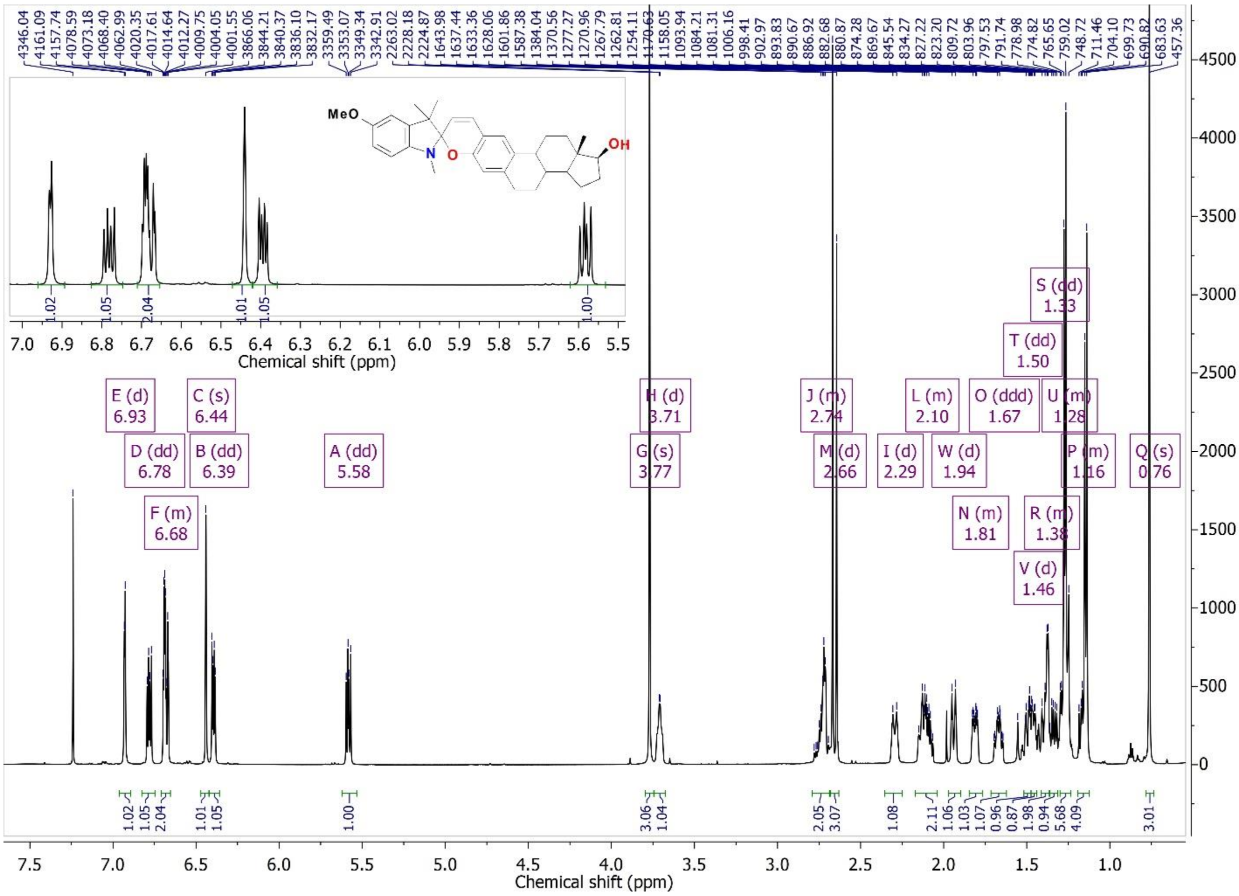

Figure S10. NMR ^{1}H spectrum of SPP (**4**)

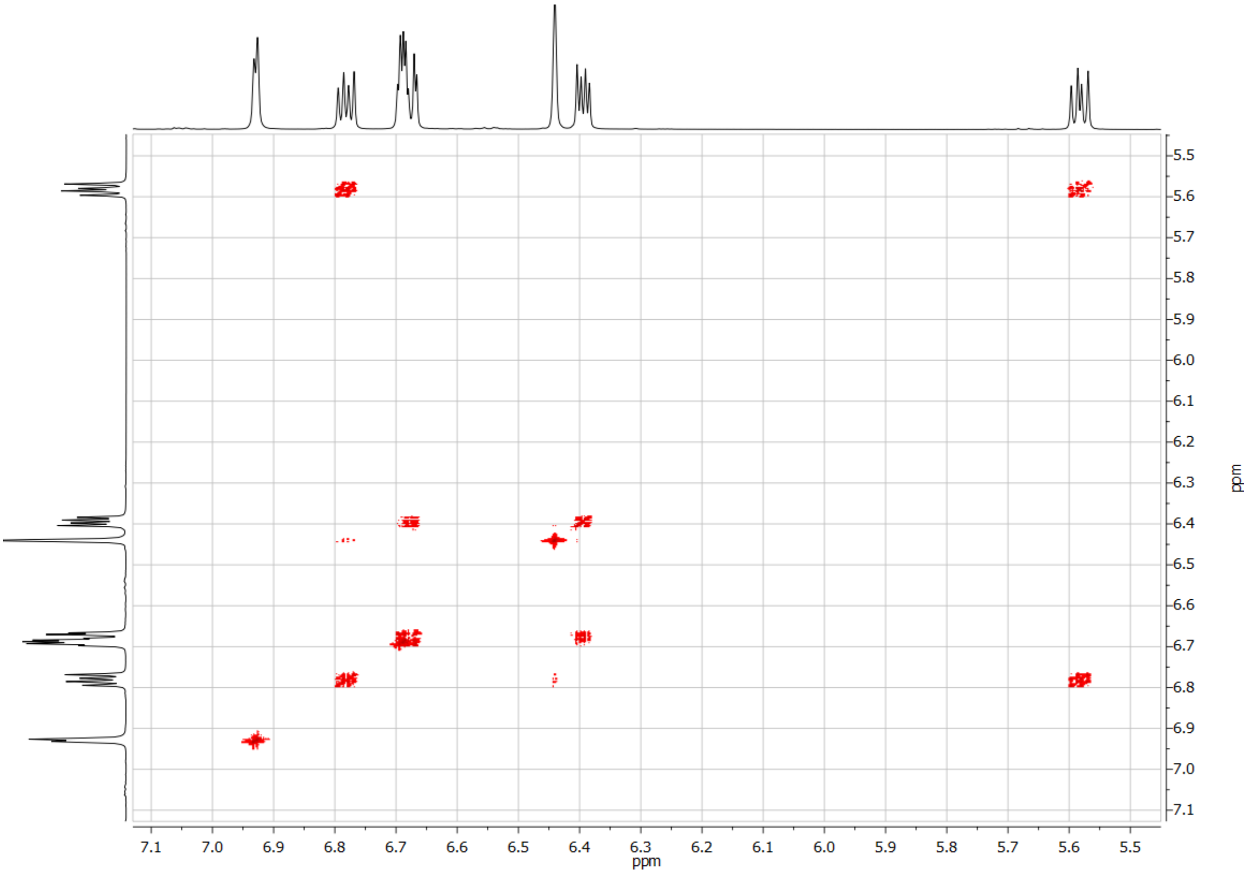

Figure S11. Fragment of COSY ^{1}H-^{1}H NMR spectrum of SPP (**4**), aromatic protons region.

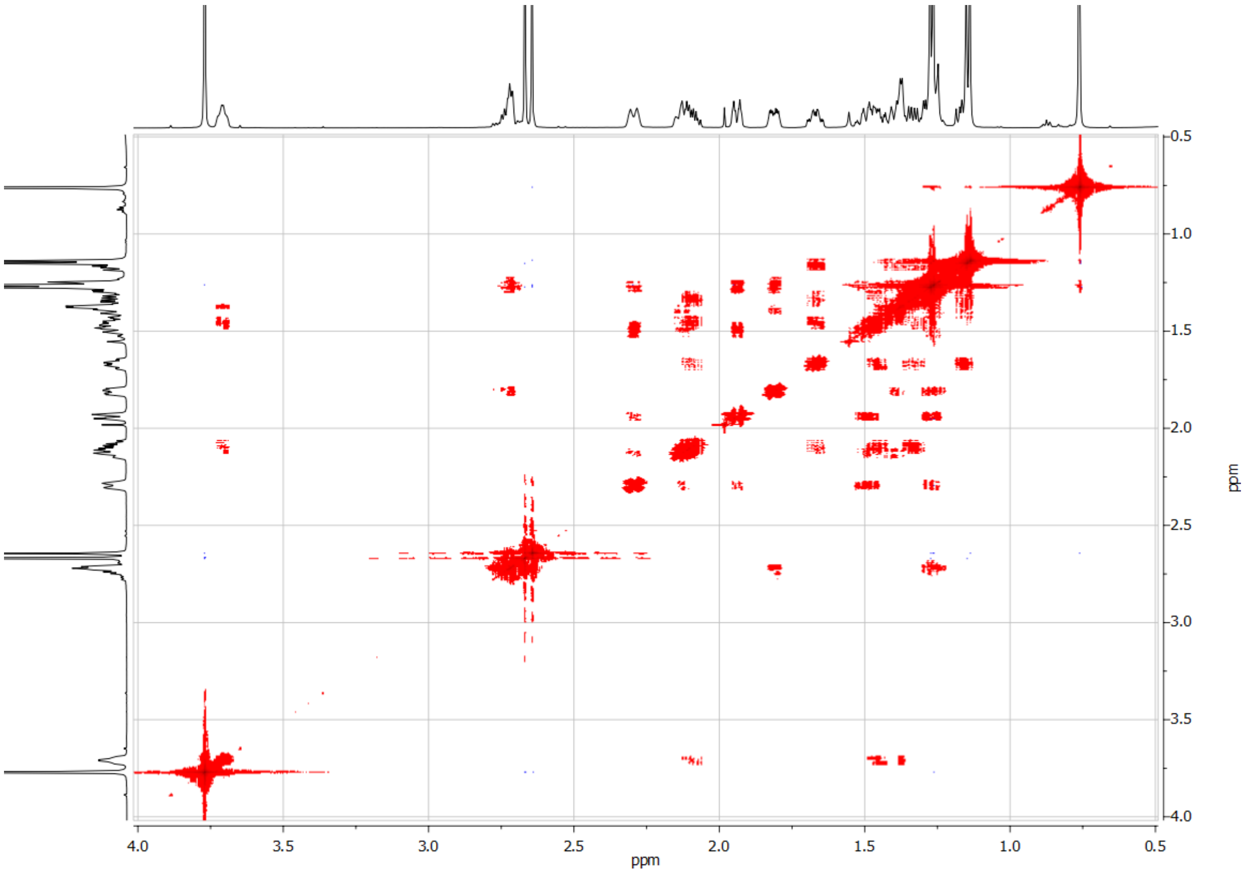

Figure S12. Fragment of COSY ^{1}H-^{1}H NMR spectrum of SPP (**4**), aliphatic protons region.

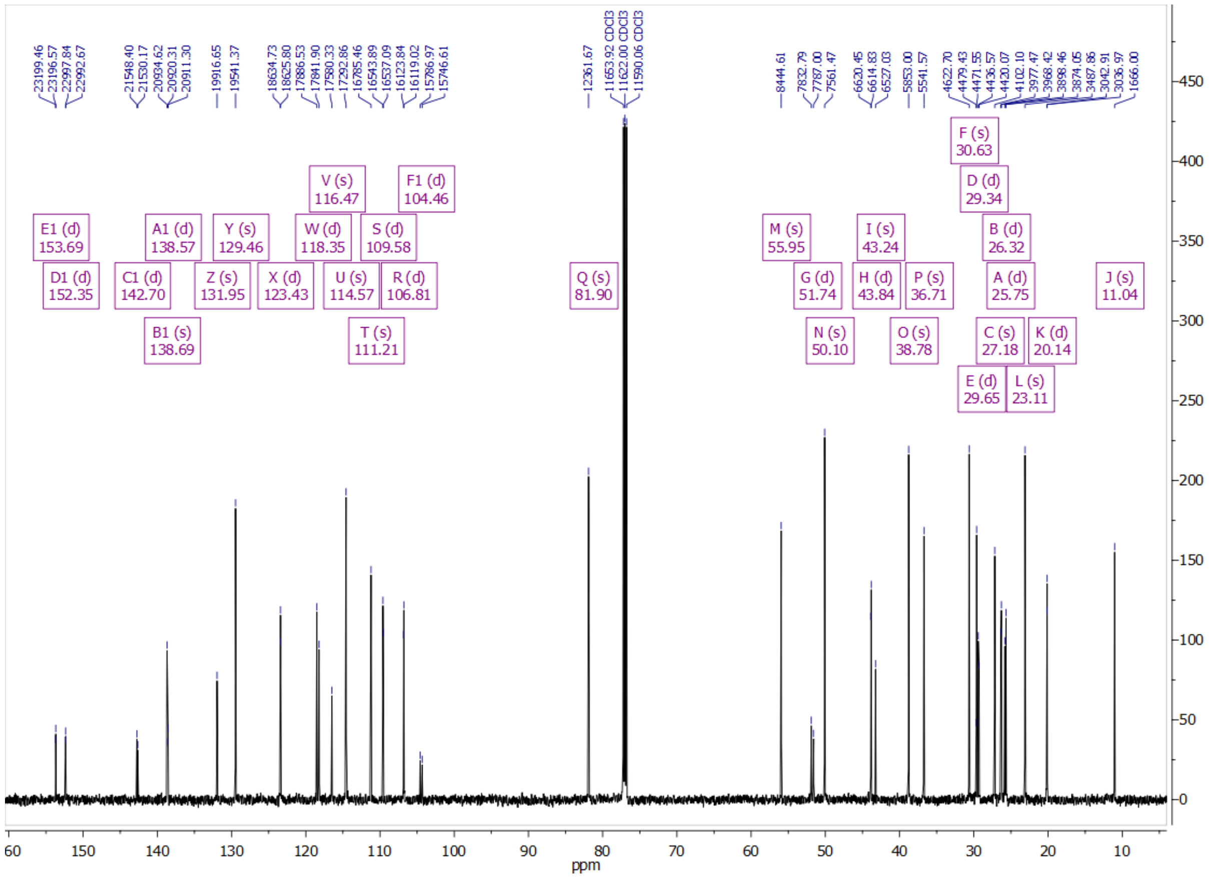

Figure S13. NMR ^{13}C spectrum of SPP (**4**).

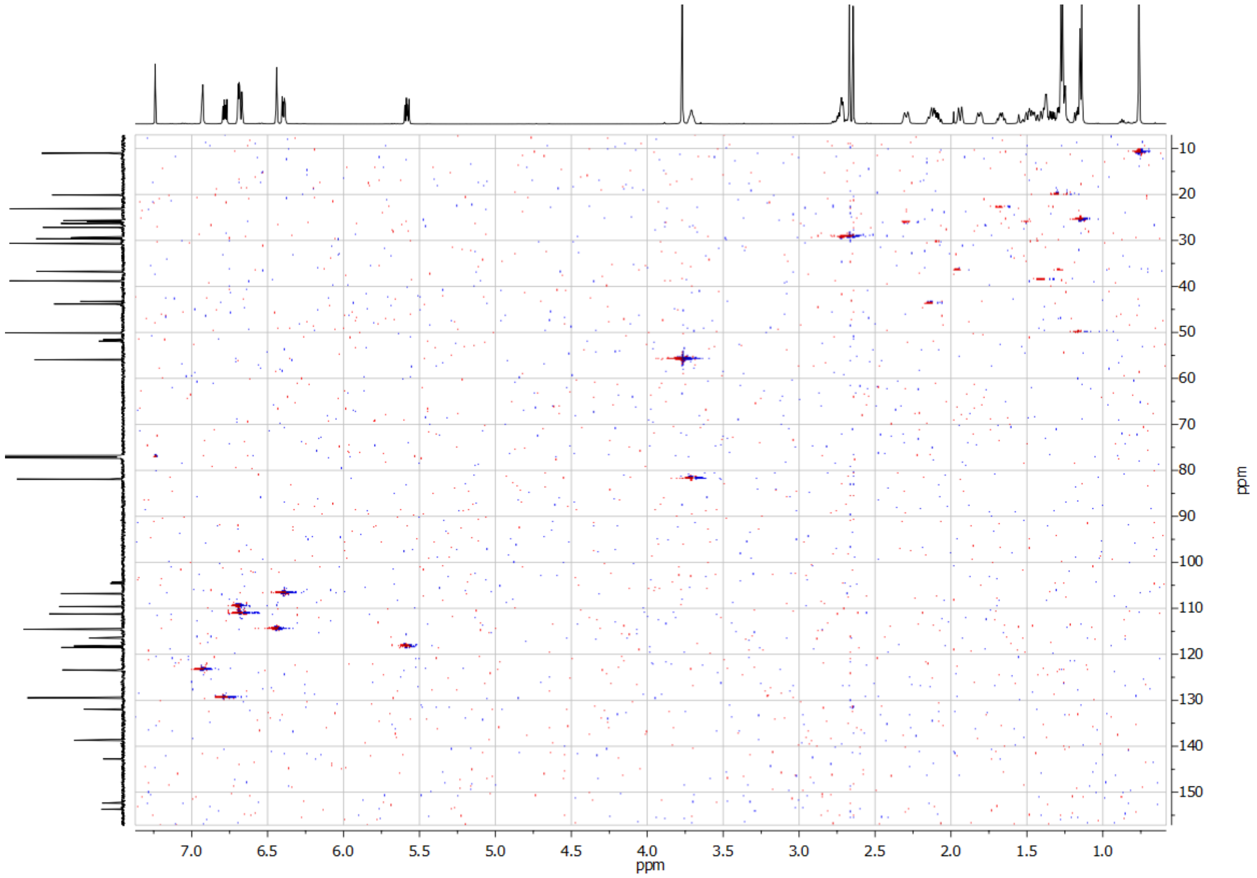

Figure S14. HSQC ^{1}H-^{13}C NMR spectrum of SPP (**4**).

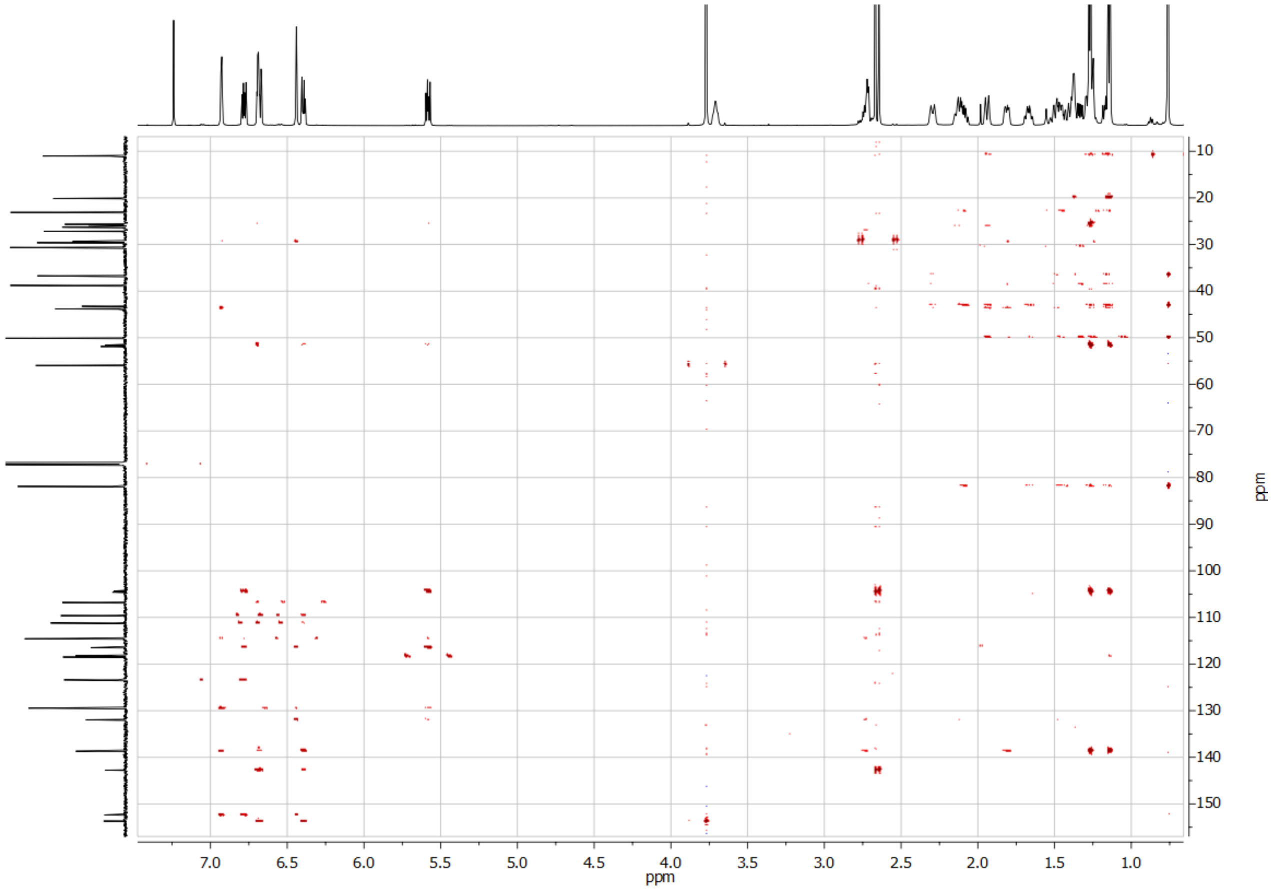

Figure S15. HMBC ^{1}H-^{13}C NMR spectrum of SPP (**4**).

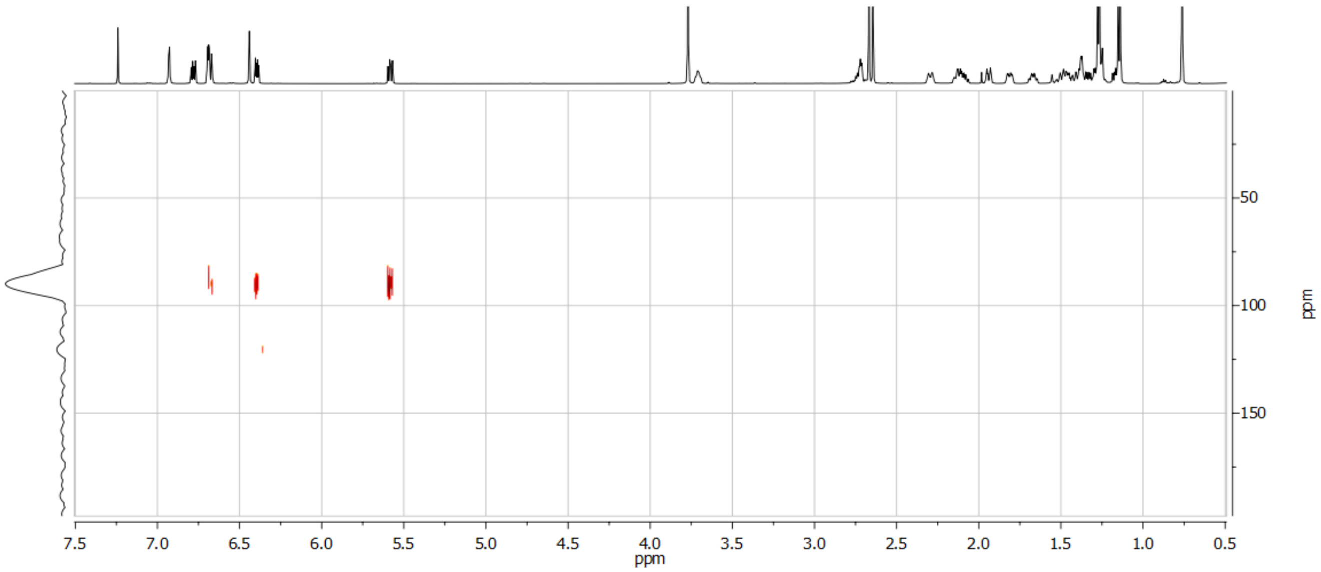

Figure S16. HMBC ^{1}H-^{15}N NMR spectrum of SPP (**4**).

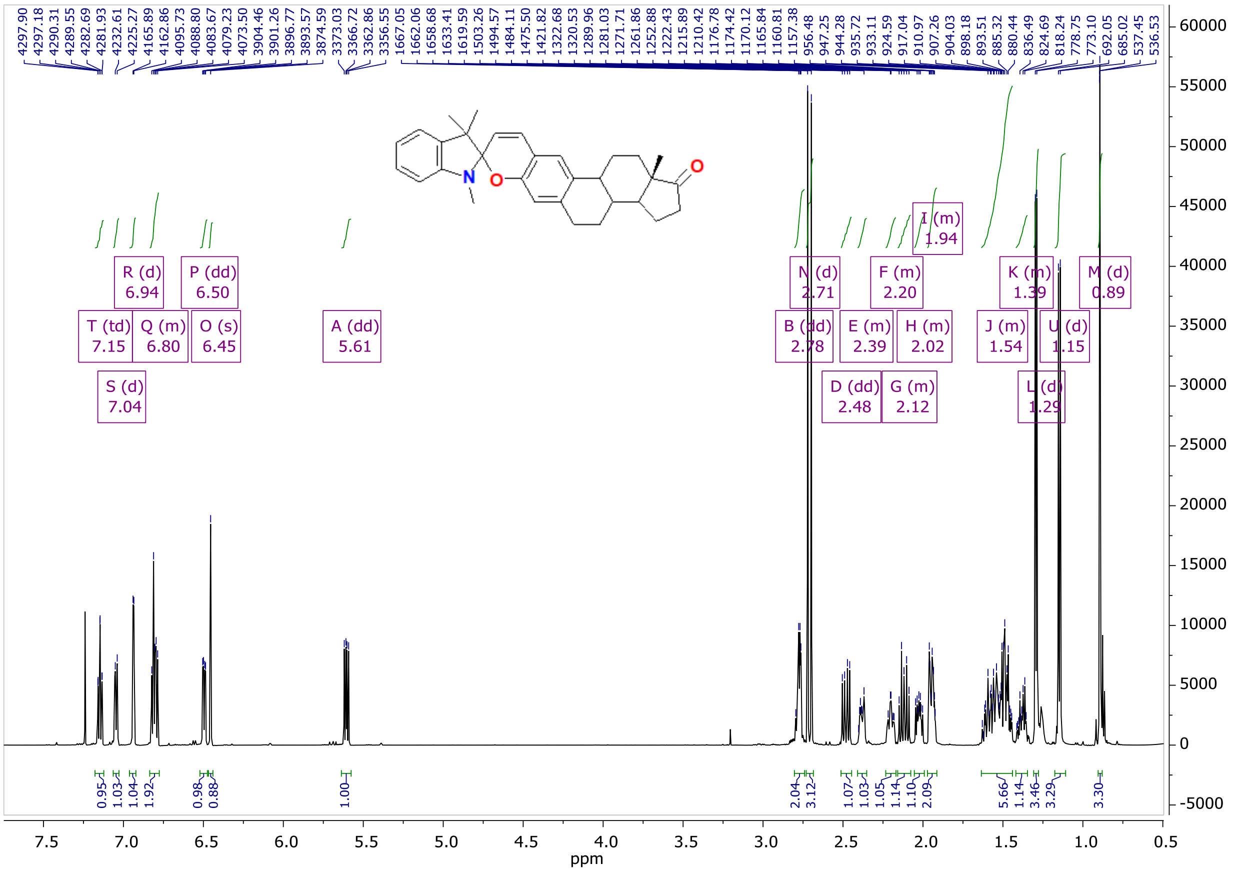

Figure S17. NMR ^{1}H spectrum of SPP (**5**).

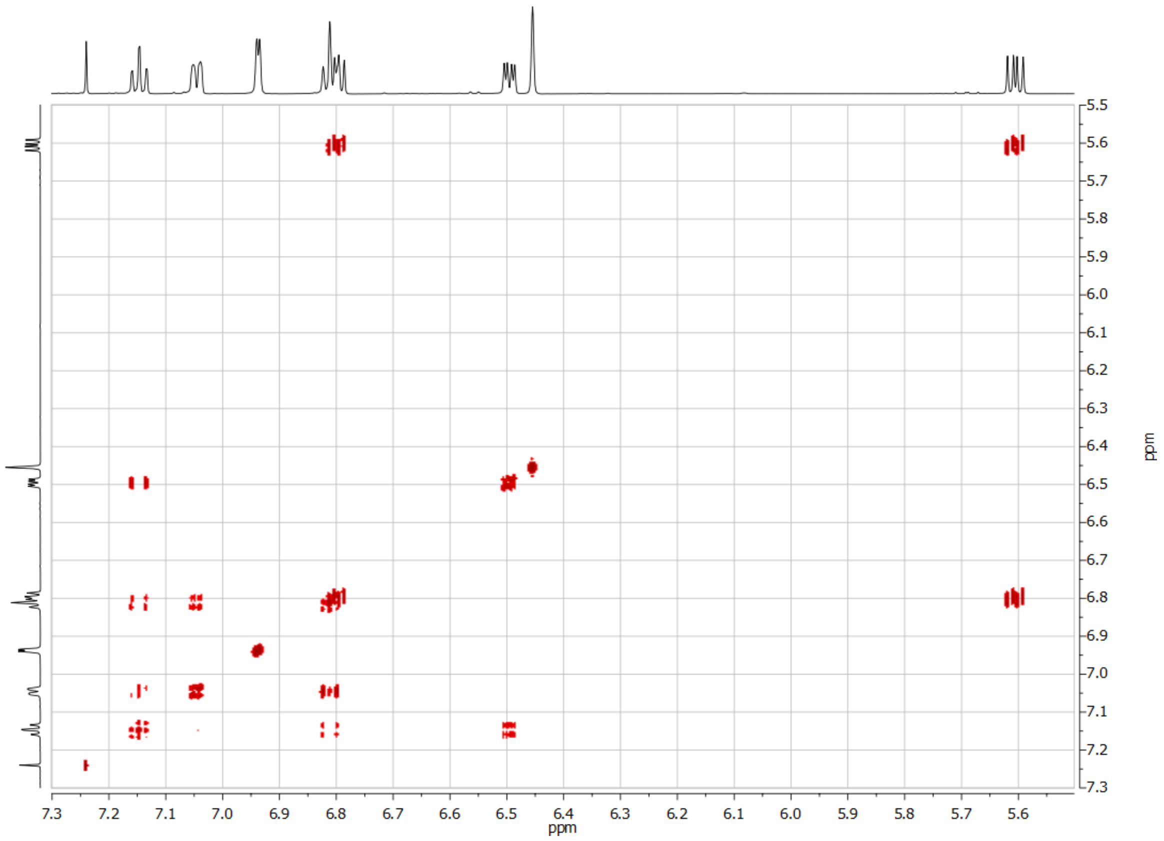

Figure S18. Fragment of COSY ^{1}H-^{1}H NMR spectrum of SPP (**5**), aromatic protons region.

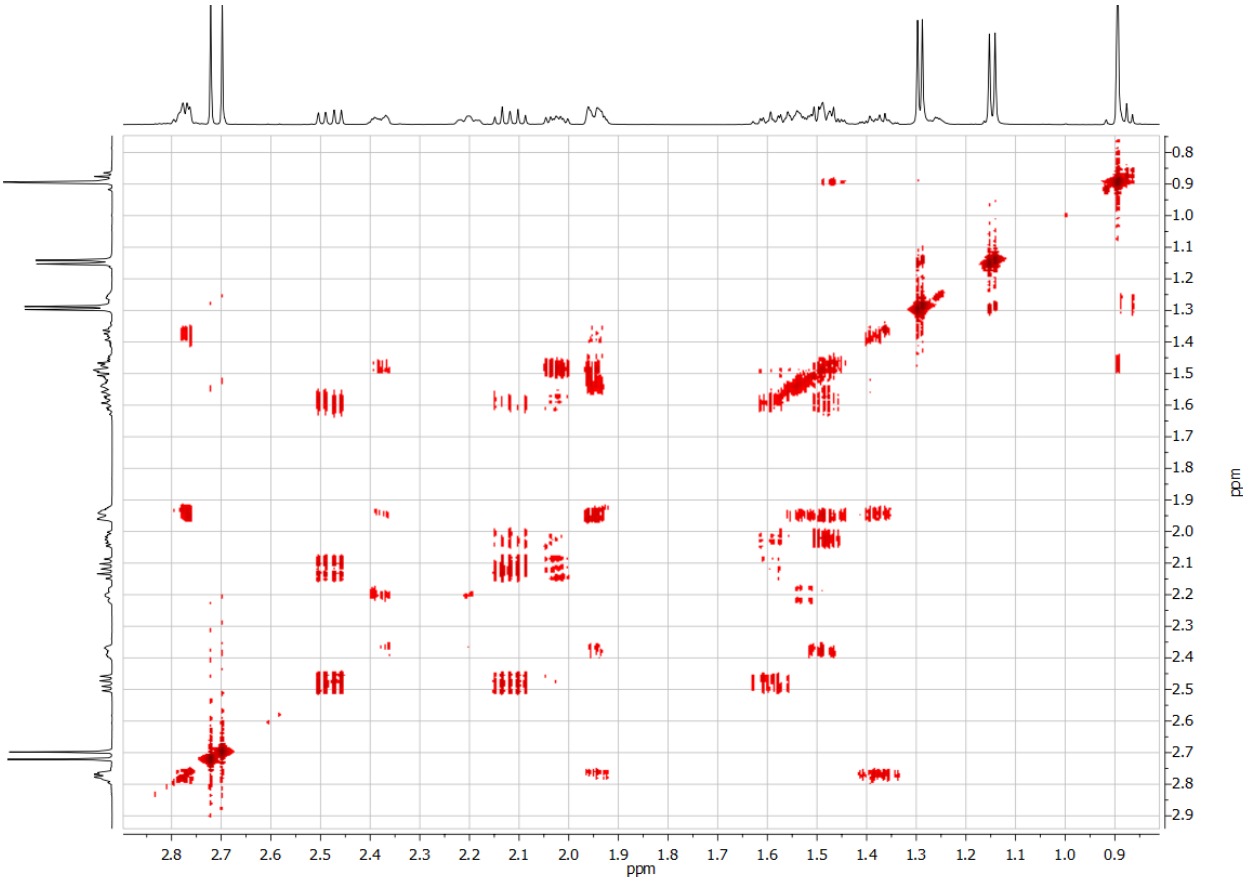

Figure S19. Fragment of COSY ^{1}H-^{1}H NMR spectrum of SPP (**5**), aliphatic protons region.

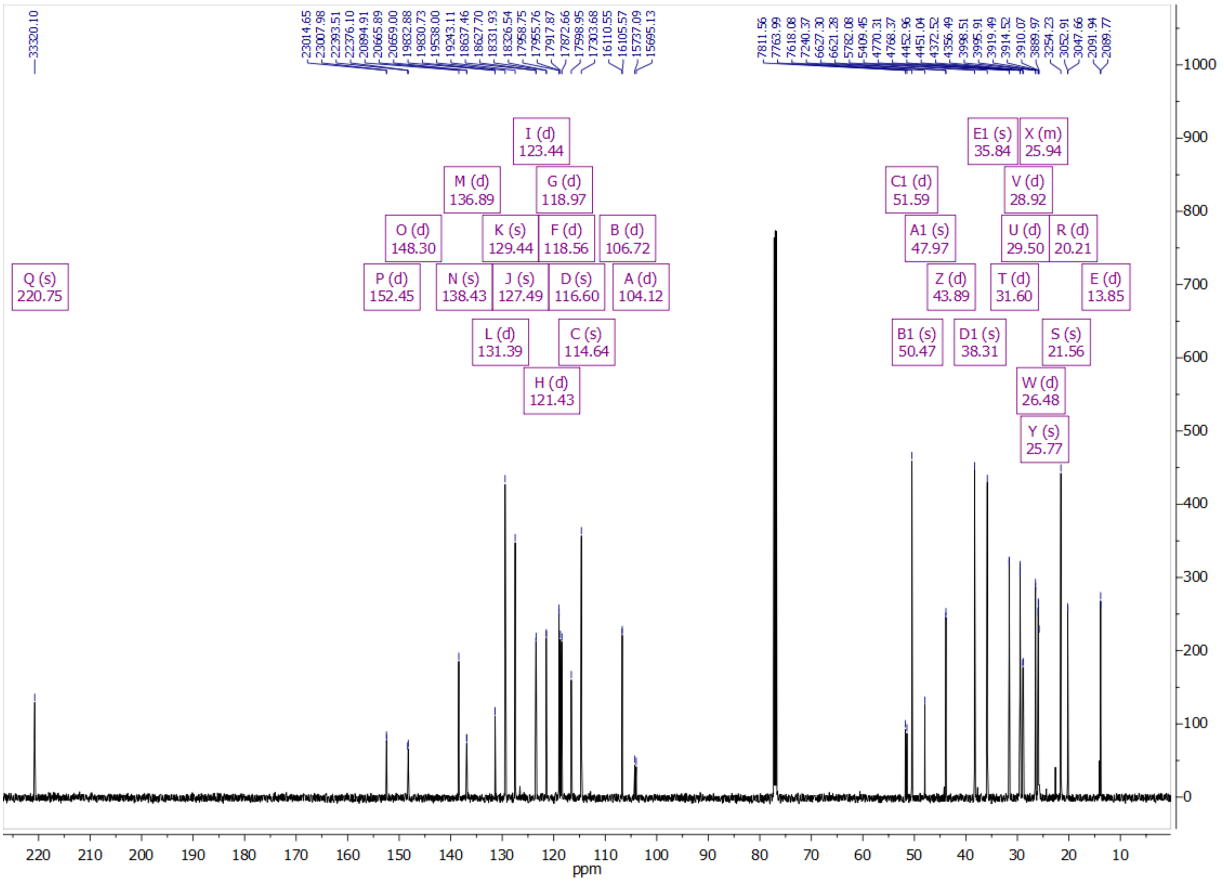

Figure S20. NMR ^{13}C spectrum of SPP (**5**).

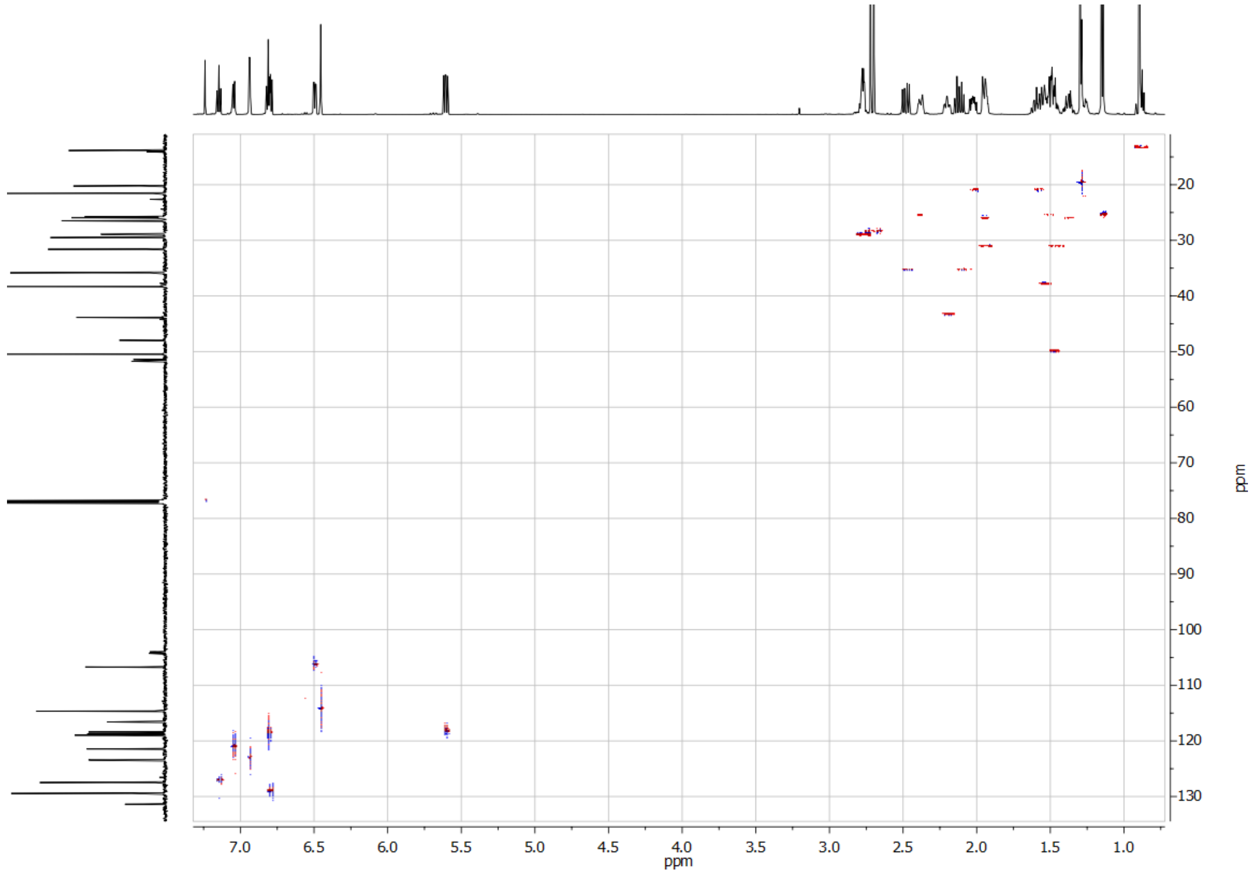

Figure S21. HSQC ^{1}H-^{13}C NMR spectrum of SPP (**5**).

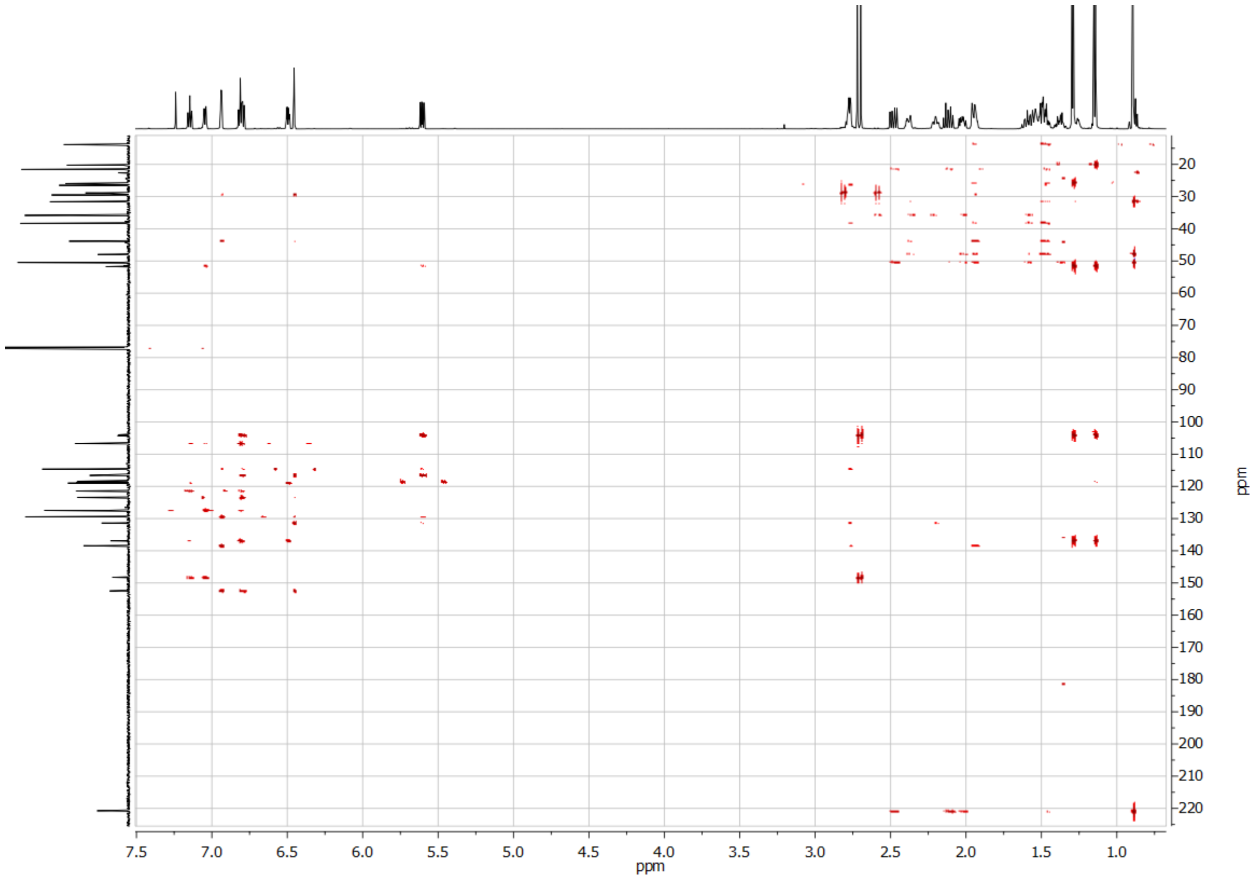

Figure S22. HMBC ^{1}H-^{13}C NMR spectrum of SPP (**5**).

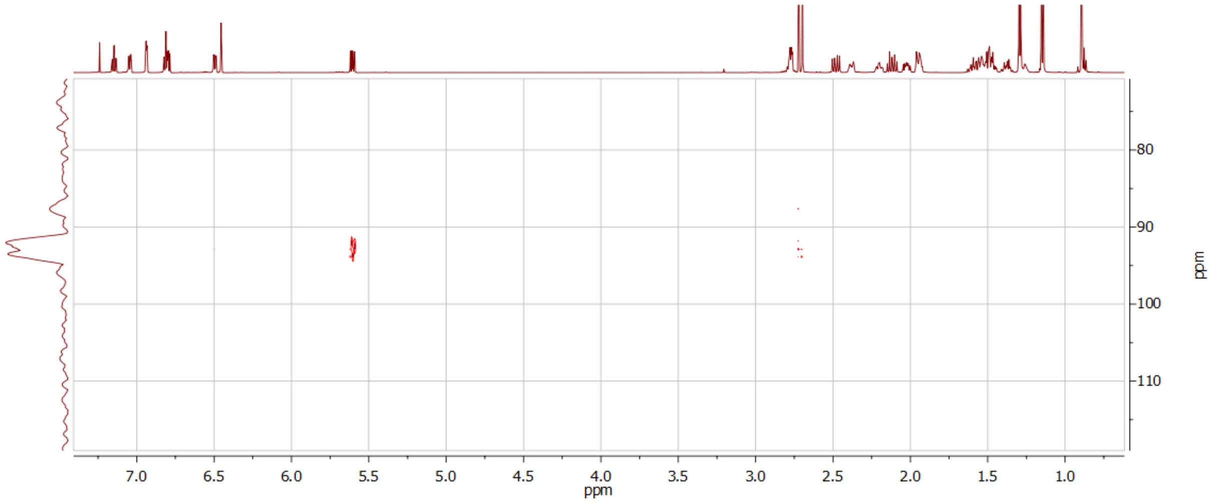

Figure S23. HMBC ^{1}H-^{15}N NMR spectrum of SPP (**5**).

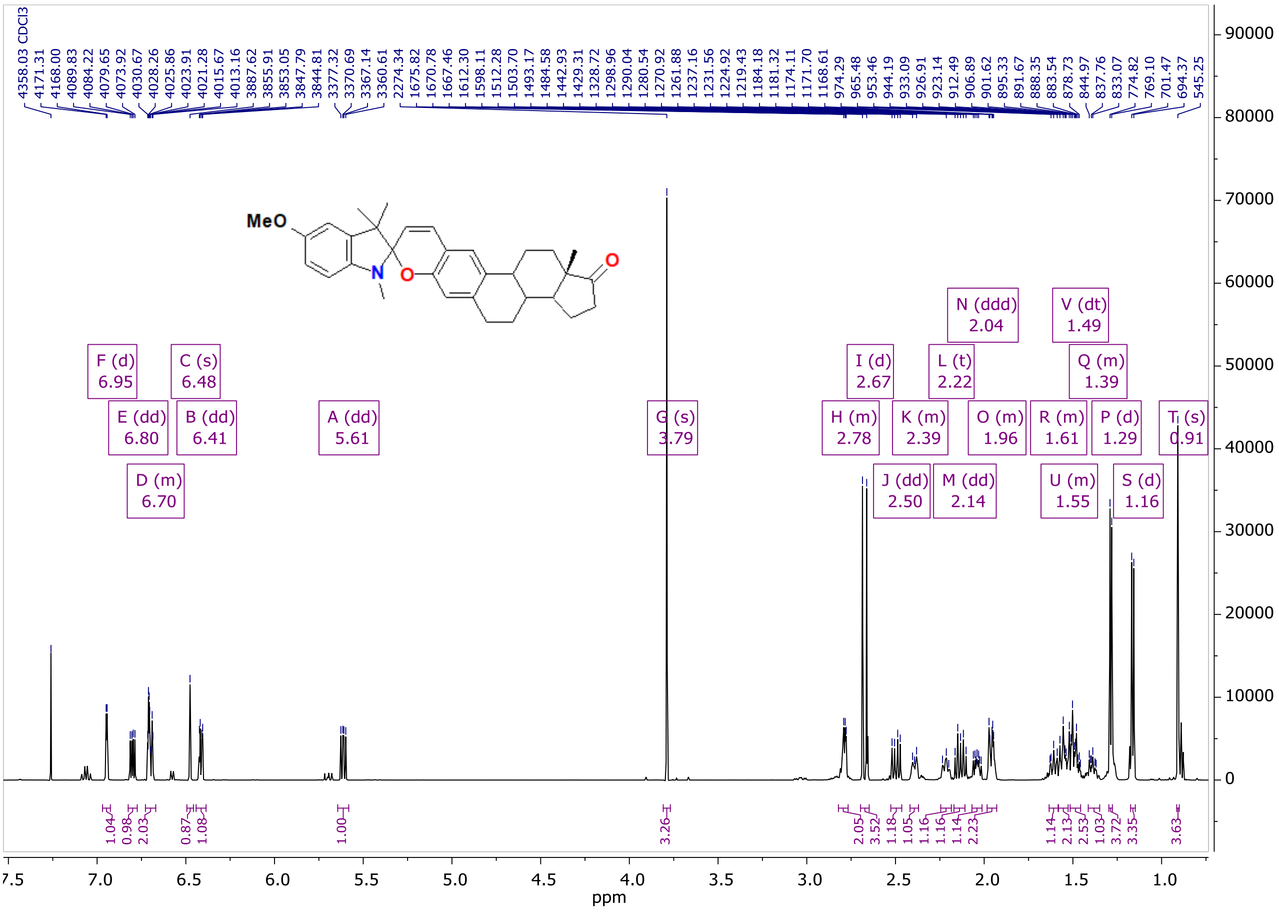

Figure S24. NMR ^{1}H spectrum of SPP (**6**).

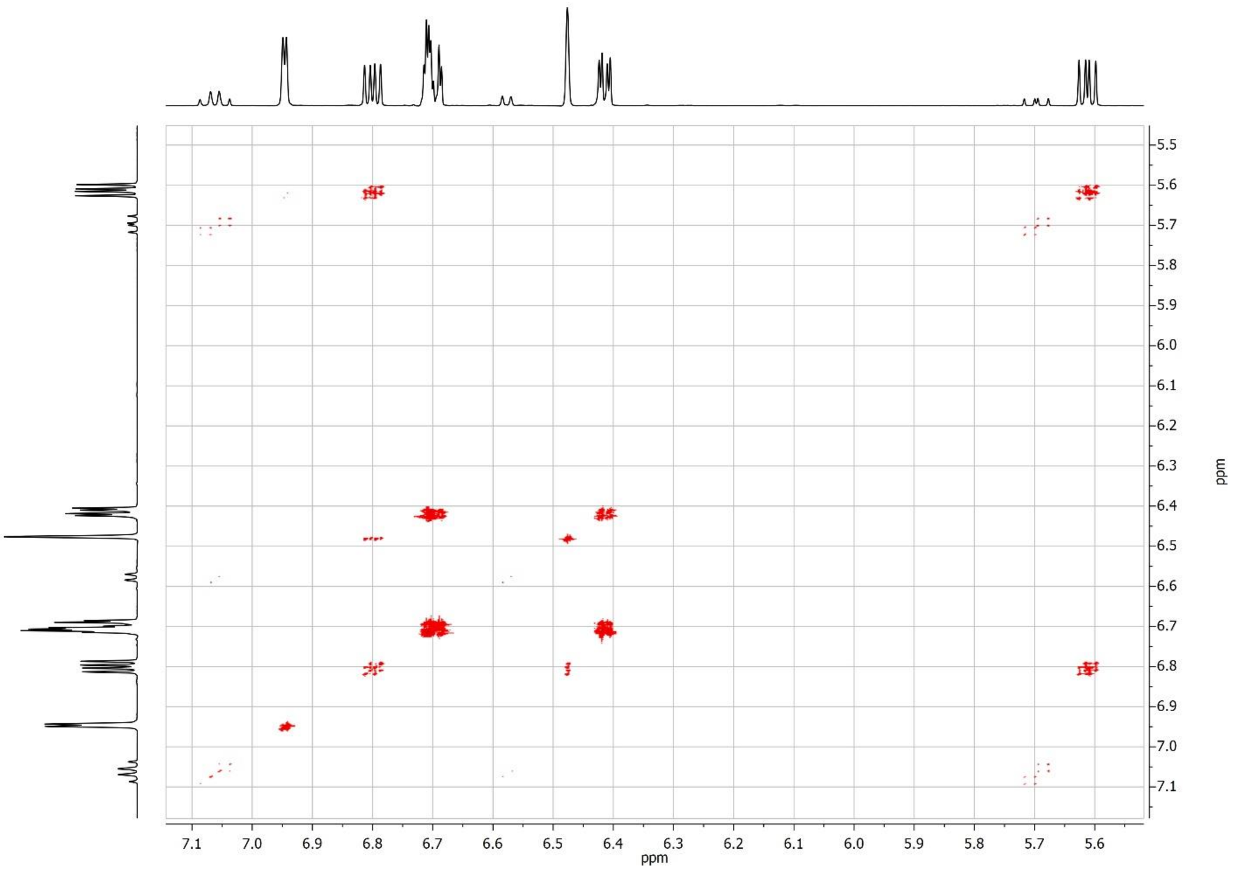

Figure S25. Fragment of COSY ^1H-^1H NMR spectrum of SPP (**6**), aromatic protons region.

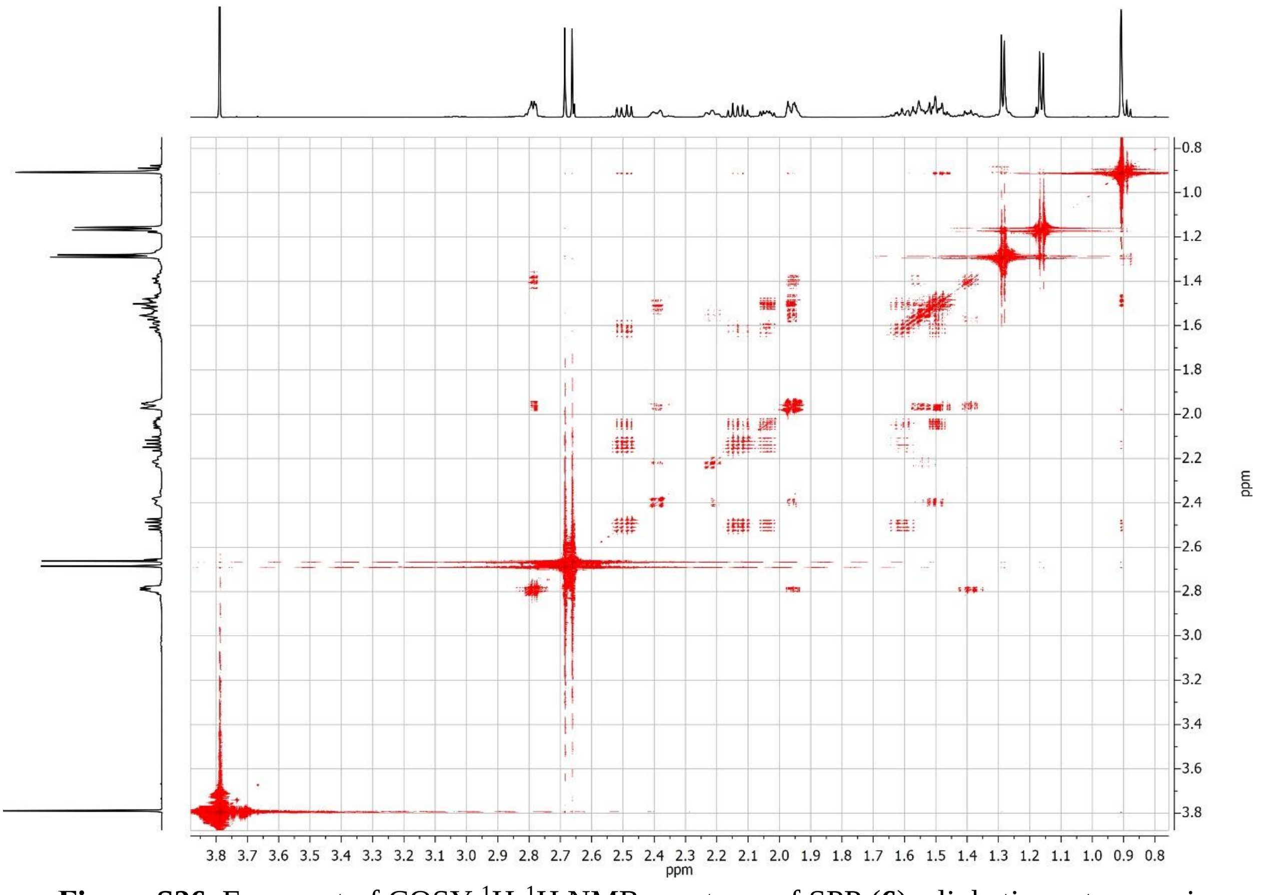

Figure S26. Fragment of COSY ^1H-^1H NMR spectrum of SPP (**6**), aliphatic protons region.

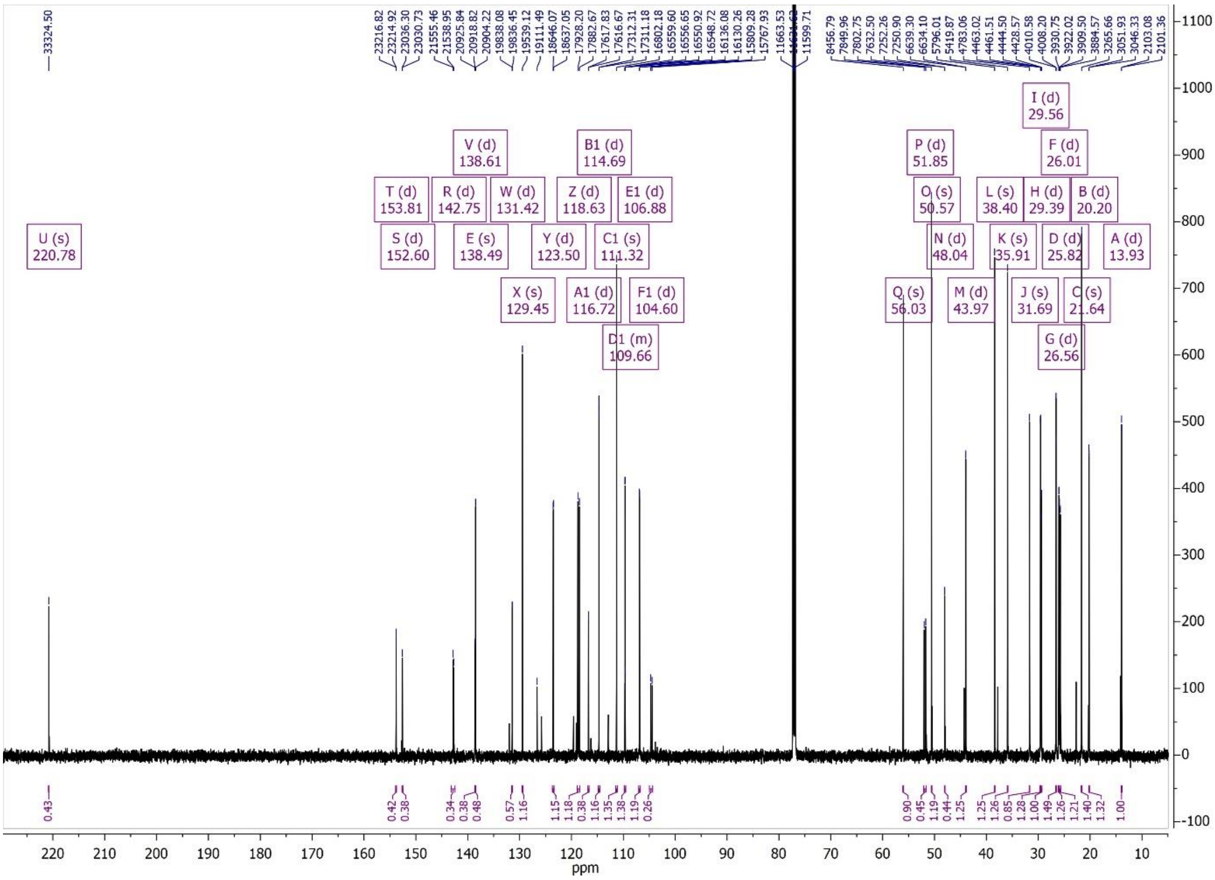

Figure S27. NMR ^{13}C spectrum of SPP (**6**).

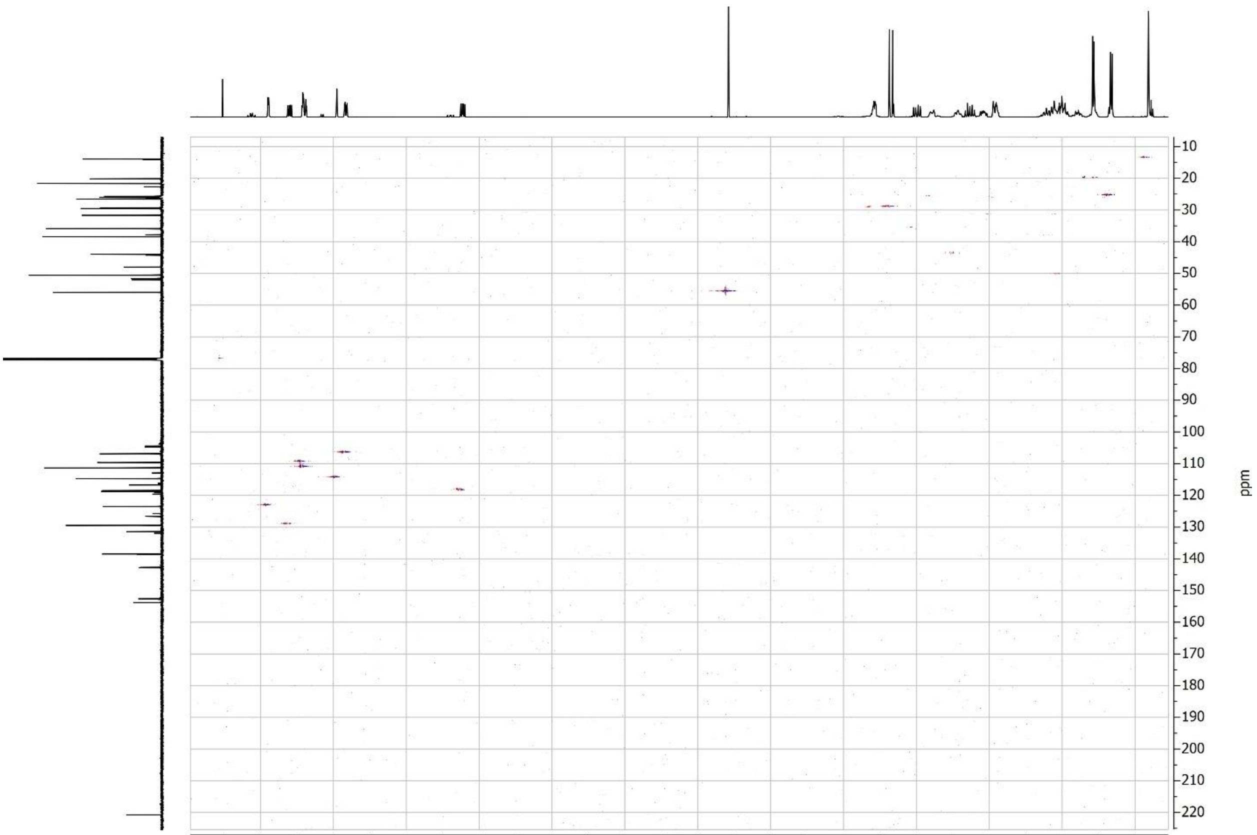

Figure S28. HSQC ^{1}H-^{13}C NMR spectrum of SPP (**6**).

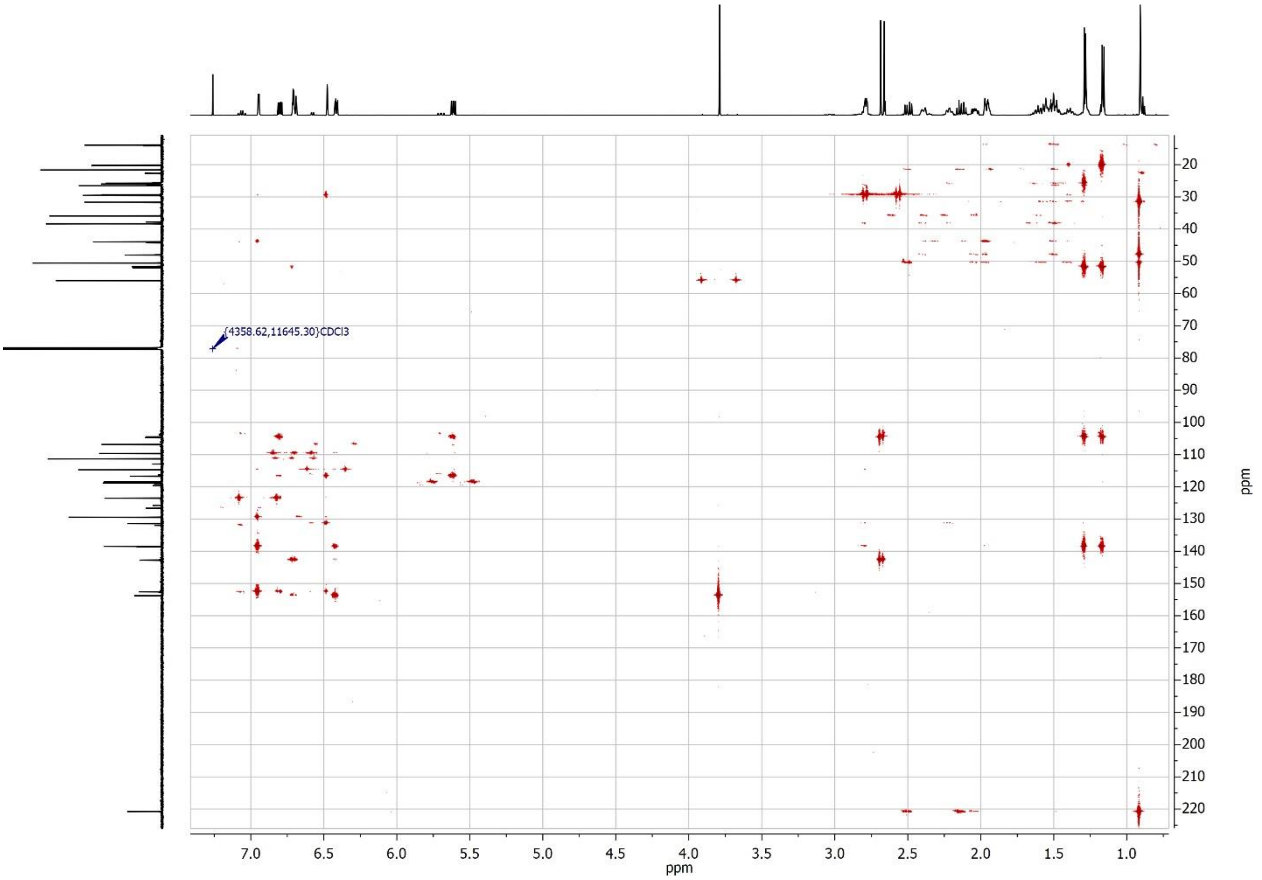

Figure S29. HMBC ^1H-^{13}C NMR spectrum of SPP (**6**).

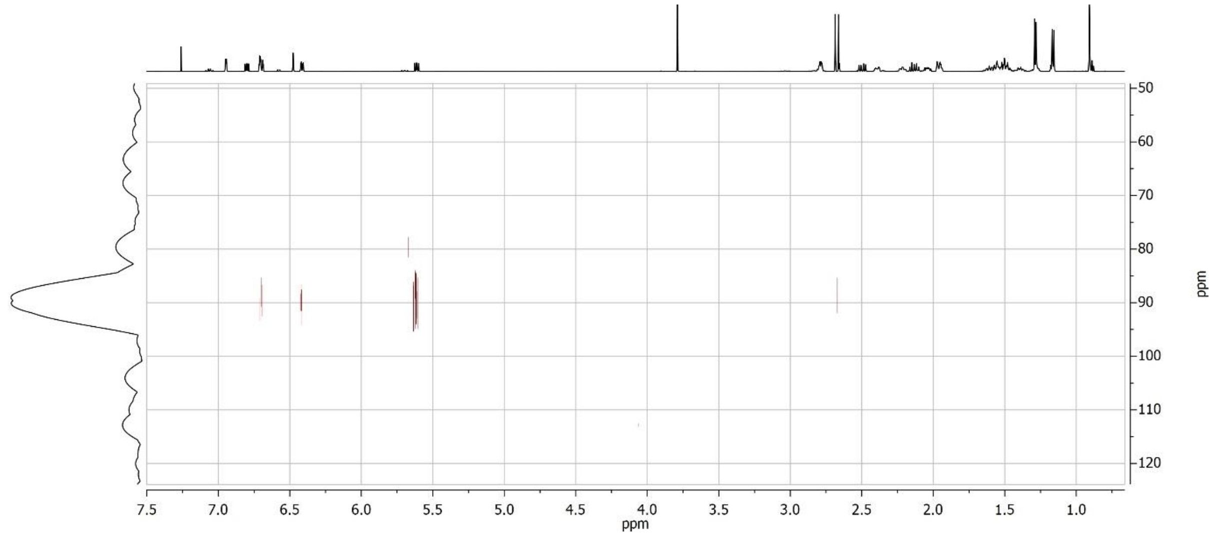

Figure S30. HMBC ^1H-^{15}N NMR spectrum of SPP (**6**).

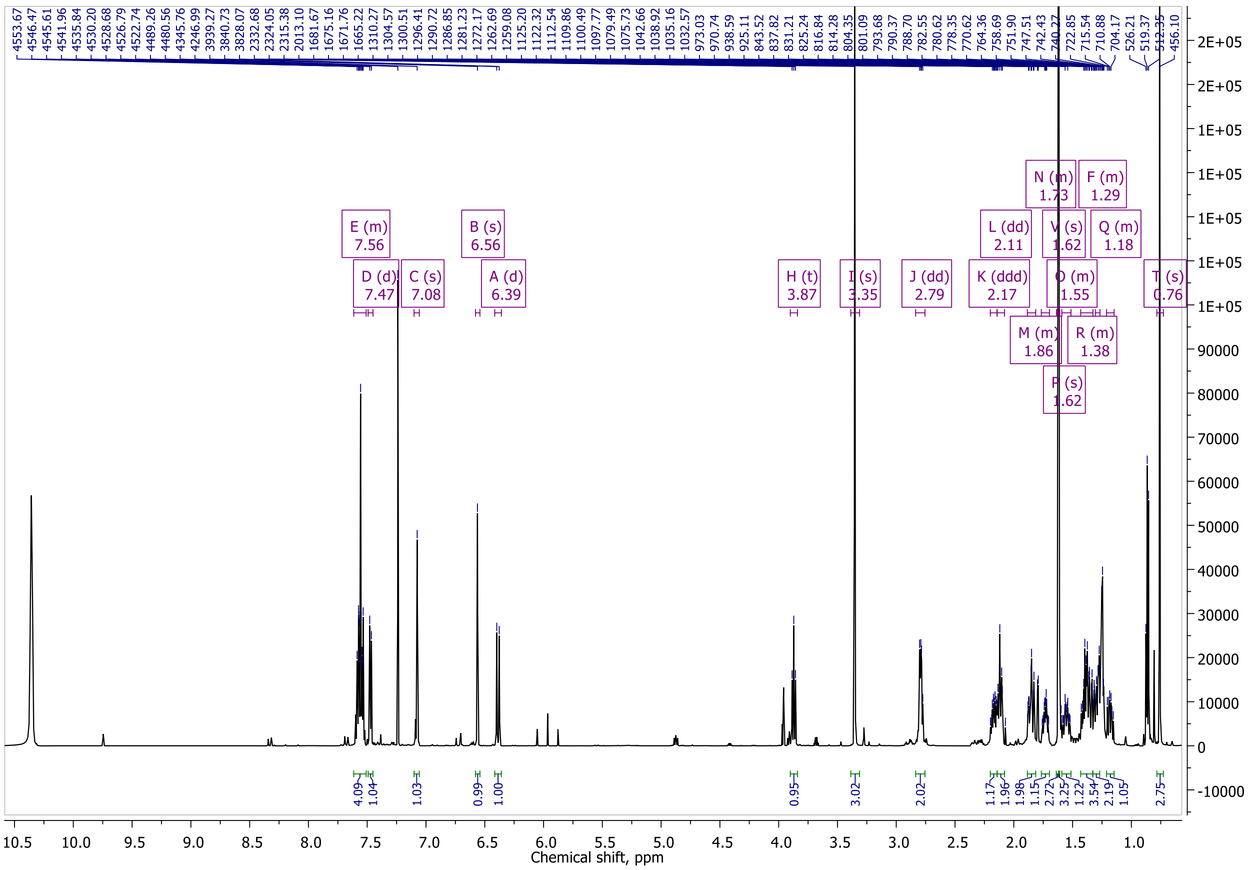

Figure S31. ^{1}H NMR spectrum of freshly prepared solution of SPP (**3**) in $CDCl_3$ with excess of TFA-*d*; CCCH form predominates.

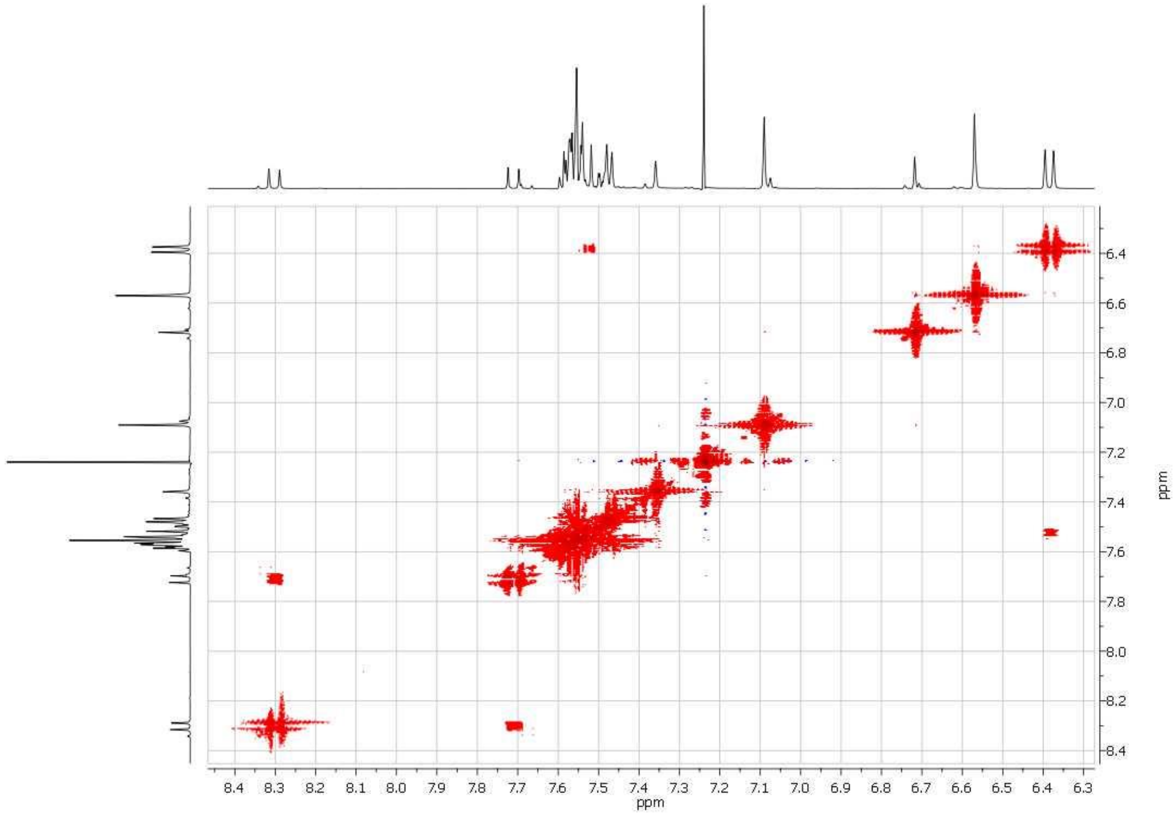

Figure S32. Fragment of COSY ^{1}H-^{1}H NMR spectrum of protonated SPP (**3**); mixture of CCCH and TTCH forms.

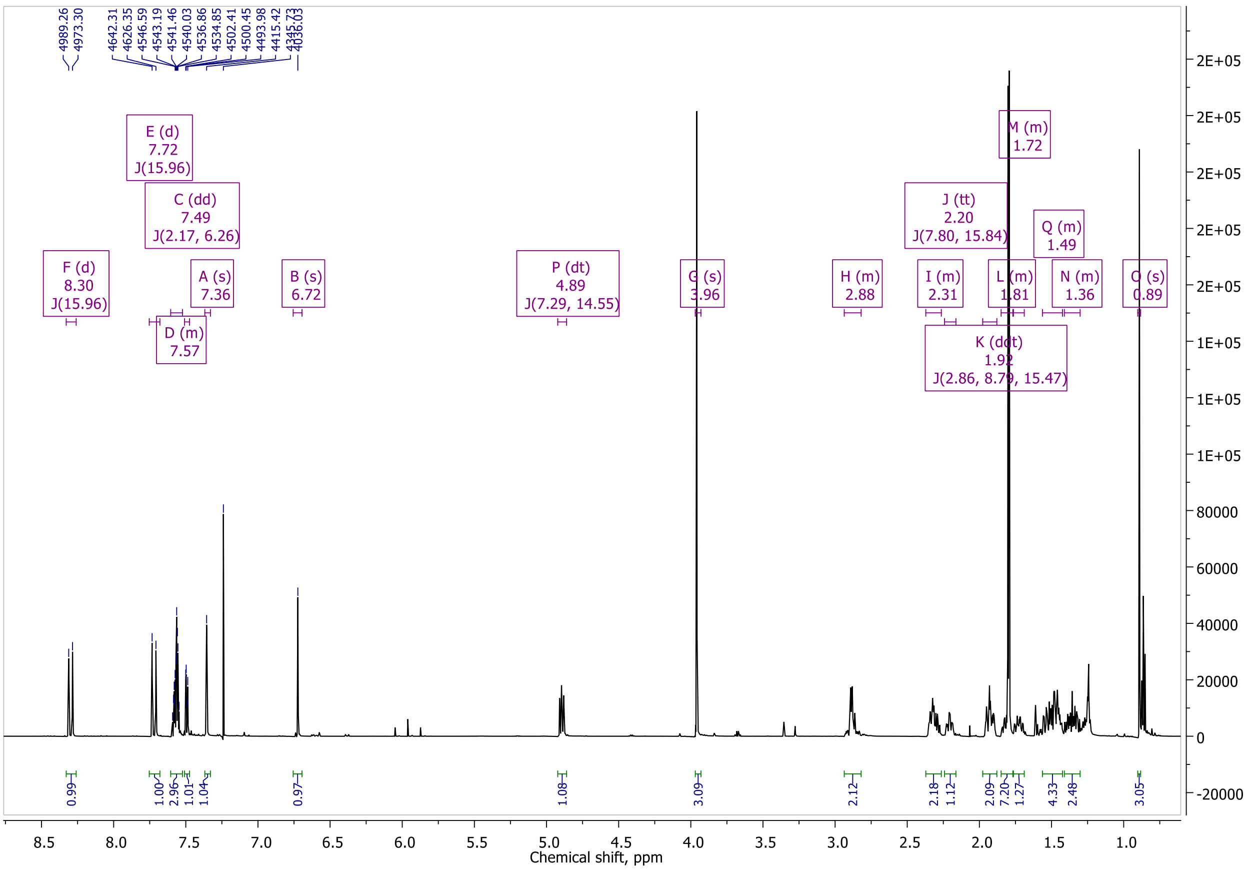

Figure S33. ^{1}H NMR spectrum of SPP (**3**) solution in $CDCl_3$ with excess of TFA-*d* registered after 24 h; TTCH form predominates.

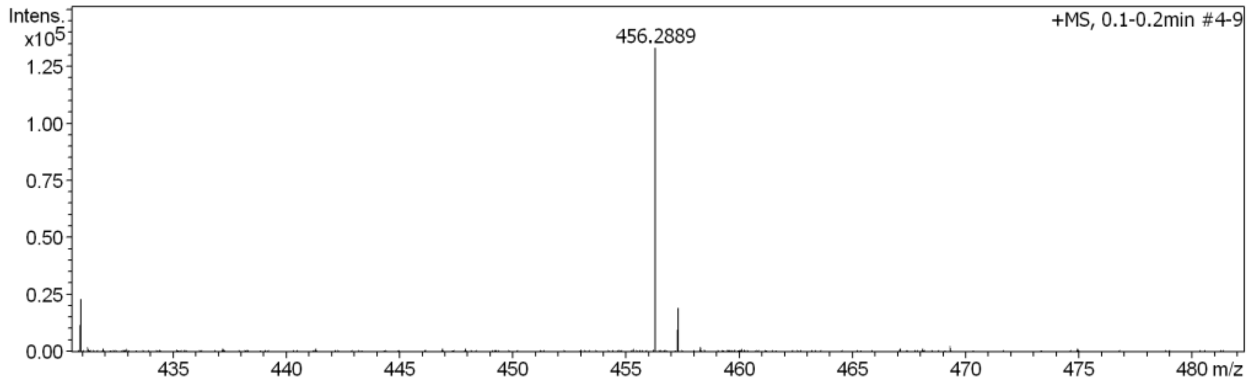

Figure S34. HRMS-spectrum of SPP (**3**).

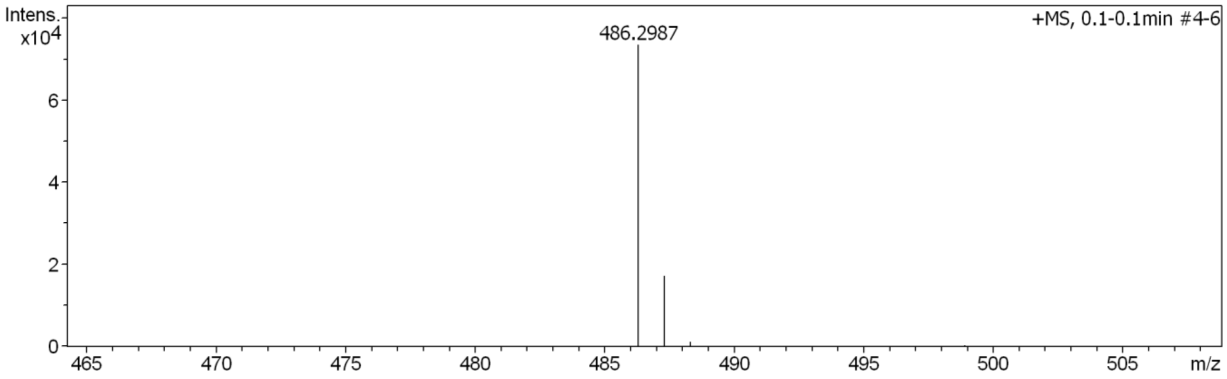

Figure S35. HRMS-spectrum of SPP (**4**).

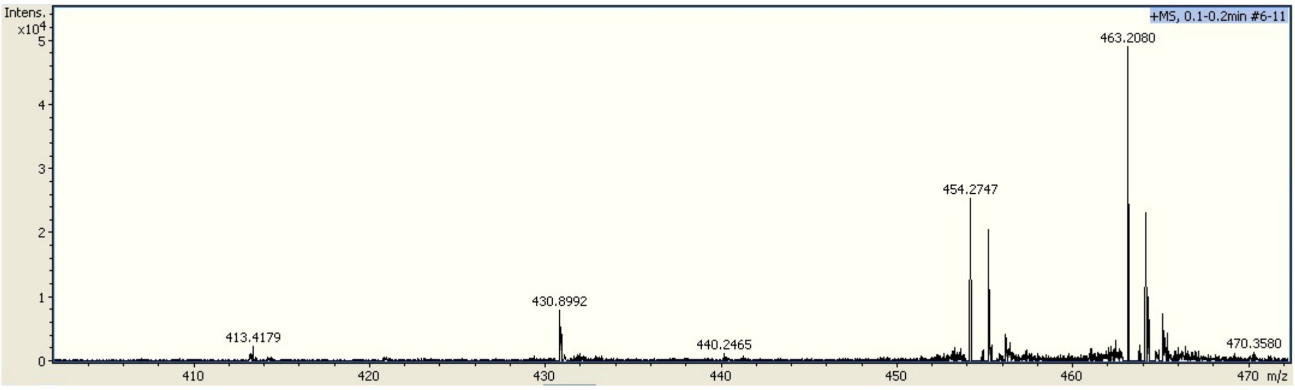

Figure S36. HRMS-spectrum of SPP (**5**).

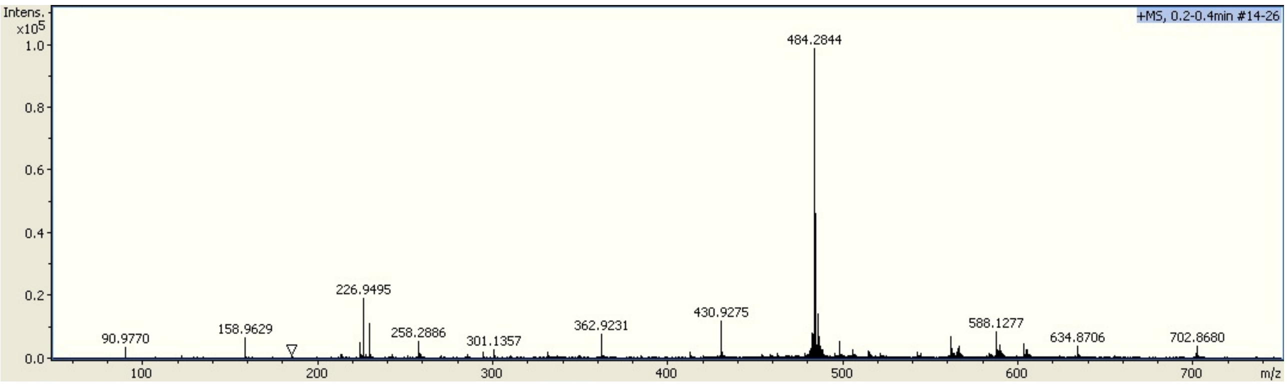

Figure S37. HRMS-spectrum of SPP (**6**).

Table S1. Crystal data and structure refinement for SPP (**3**).

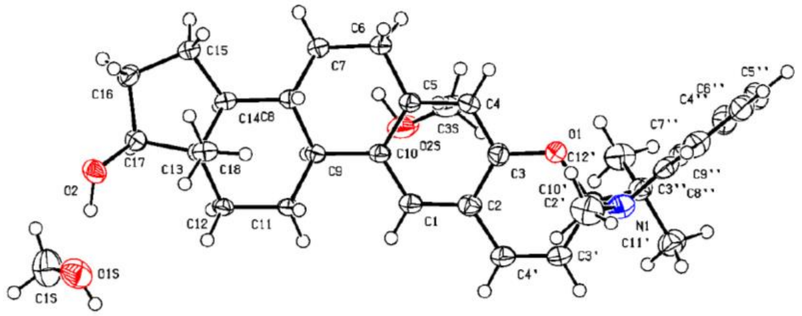

CCDC Number	2163146
Empirical formula	$C_{33}H_{45}NO_4$
Formula weight	519.70
Temperature/K	100.00(10)
Crystal system	monoclinic
Space group	$P2_1$
a/Å	6.60840(10)
b/Å	11.1387(2)
c/Å	19.9797(4)
α/°	90
β/°	92.557(2)
γ/°	90
Volume/Å^3	1469.22(5)
Z	2
ρ_{calc}/g·cm^{-3}	1.175
μ/mm^{-1}	0.597
F(000)	564.0
Crystal size/mm^3	0.458 × 0.376 × 0.195
Radiation	Cu Kα (λ = 1.54184)
2Θ range for data collection/°	4.428 to 152.858
Index ranges	$-8 \leq h \leq 8$, $-14 \leq k \leq 14$, $-24 \leq l \leq 25$
Reflections collected	39389
Independent reflections	6141 [R_{int} = 0.0493, R_{sigma} = 0.0254]
Data/restraints/parameters	6141/1/358
Goodness-of-fit on F^2	1.111
Final R indexes [I>=2σ (I)]	R_1 = 0.0623, wR_2 = 0.1731
Final R indexes [all data]	R_1 = 0.0628, wR_2 = 0.1736
Largest diff. peak/hole / e Å^{-3}	0.54/-0.27
Flack parameter	0.04(14)

Table S2. Bond Lengths for SPP (**3**).

Atom	Atom	Length/Å	Atom	Atom	Length/Å
O2	C17	1.421(5)	C17	C16	1.550(5)
O1	C3	1.366(5)	C4	C5	1.399(5)
O1	C2'	1.457(5)	C5	C6	1.512(5)
O1S	C1S	1.385(8)	C5	C10	1.412(5)
O2S	C3S	1.413(6)	C15	C16	1.554(5)

Table S2. Bond Lengths for SPP (**3**).

Atom	Atom	Length/Å	Atom	Atom	Length/Å
N1	C8"	1.398(6)	C6	C7	1.528(5)
N1	C2'	1.463(6)	C8"	C9"	1.394(7)
N1	C10'	1.438(7)	C8"	C7"	1.390(6)
C3	C4	1.384(6)	C1	C2	1.391(6)
C3	C2	1.396(5)	C1	C10	1.394(5)
C8	C14	1.531(5)	C2'	C3"	1.544(6)
C8	C9	1.541(5)	C2'	C3'	1.505(6)
C8	C7	1.528(5)	C2	C4'	1.446(6)
C14	C13	1.539(5)	C9"	C3"	1.511(6)
C14	C15	1.537(5)	C9"	C4"	1.375(7)
C12	C11	1.539(5)	C3"	C11'	1.544(6)
C12	C13	1.534(5)	C3"	C12'	1.536(7)
C11	C9	1.536(5)	C4'	C3'	1.334(6)
C9	C10	1.520(5)	C5"	C4"	1.405(7)
C13	C17	1.541(5)	C5"	C6"	1.387(8)
C13	C18	1.528(5)	C7"	C6"	1.400(7)

Table S3. Bond Angles for SPP (**3**).

Atom	Atom	Atom	Angle/°	Atom	Atom	Atom	Angle/°
C3	O1	C2'	122.8(3)	C9"	C8"	N1	110.2(4)
C8"	N1	C2'	106.7(4)	C7"	C8"	N1	128.4(5)
C8"	N1	C10'	119.6(4)	C7"	C8"	C9"	121.4(5)
C10'	N1	C2'	117.9(4)	C6	C7	C8	109.9(3)
O1	C3	C4	117.1(3)	C2	C1	C10	122.9(4)
O1	C3	C2	122.2(4)	O1	C2'	N1	107.6(3)
C4	C3	C2	120.7(4)	O1	C2'	C3"	106.2(4)
C14	C8	C9	108.1(3)	O1	C2'	C3'	112.9(3)
C7	C8	C14	113.8(3)	N1	C2'	C3"	102.7(3)
C7	C8	C9	108.8(3)	N1	C2'	C3'	112.8(4)
C8	C14	C13	112.5(3)	C3'	C2'	C3"	114.0(4)
C8	C14	C15	119.7(3)	C3	C2	C4'	117.8(4)
C15	C14	C13	104.0(3)	C1	C2	C3	118.1(4)
C13	C12	C11	111.2(3)	C1	C2	C4'	124.1(4)
C9	C11	C12	112.6(3)	C5	C10	C9	121.3(3)
C11	C9	C8	111.6(3)	C1	C10	C9	120.9(3)
C10	C9	C8	110.9(3)	C1	C10	C5	117.8(3)
C10	C9	C11	113.1(3)	C8"	C9"	C3"	108.0(4)
C14	C13	C17	98.8(3)	C4"	C9"	C8"	120.6(4)
C12	C13	C14	108.2(3)	C4"	C9"	C3"	131.2(4)
C12	C13	C17	115.1(3)	C17	C16	C15	105.3(3)
C18	C13	C14	113.8(3)	C9"	C3"	C2'	100.9(3)
C18	C13	C12	110.9(3)	C9"	C3"	C11'	108.2(4)
C18	C13	C17	109.7(3)	C9"	C3"	C12'	114.5(4)
O2	C17	C13	116.0(3)	C11'	C3"	C2'	112.4(4)
O2	C17	C16	110.2(3)	C12'	C3"	C2'	112.8(4)

Table S3. Bond Angles for SPP (**3**).

Atom	Atom	Atom	Angle/°	Atom	Atom	Atom	Angle/°
C13	C17	C16	103.9(3)	C12'	C3"	C11'	107.9(4)
C3	C4	C5	120.6(3)	C3'	C4'	C2	121.5(4)
C4	C5	C6	118.2(3)	C6"	C5"	C4"	119.4(5)
C4	C5	C10	119.8(3)	C9"	C4"	C5"	119.3(5)
C10	C5	C6	122.0(3)	C4'	C3'	C2'	122.6(4)
C14	C15	C16	104.0(3)	C8"	C7"	C6"	117.3(5)
C5	C6	C7	113.4(3)	C5"	C6"	C7"	122.0(4)

Table S4. Torsion Angles for SPP (**3**).

A	B	C	D	Angle/°	A	B	C	D	Angle/°
O2	C17	C16	C15	150.5(3)	C4	C5	C10	C1	-3.9(6)
O1	C3	C4	C5	178.2(4)	C5	C6	C7	C8	43.1(5)
O1	C3	C2	C1	-179.9(4)	C18	C13	C17	O2	-46.5(4)
O1	C3	C2	C4'	0.1(6)	C18	C13	C17	C16	74.7(4)
O1	C2'	C3"	C9"	81.6(4)	C15	C14	C13	C12	167.9(3)
O1	C2'	C3"	C11'	-163.4(4)	C15	C14	C13	C17	47.7(3)
O1	C2'	C3"	C12'	-41.1(5)	C15	C14	C13	C18	-68.4(4)
O1	C2'	C3'	C4'	-4.5(7)	C6	C5	C10	C9	-4.1(6)
N1	C8"	C9"	C3"	-0.3(5)	C6	C5	C10	C1	174.5(4)
N1	C8"	C9"	C4"	-177.0(4)	C8"	N1	C2'	O1	-79.0(4)
N1	C8"	C7"	C6"	177.3(4)	C8"	N1	C2'	C3"	32.8(4)
N1	C2'	C3"	C9"	-31.2(4)	C8"	N1	C2'	C3'	156.0(4)
N1	C2'	C3"	C11'	83.8(4)	C8"	C9"	C3"	C2'	19.9(4)
N1	C2'	C3"	C12'	-153.9(4)	C8"	C9"	C3"	C11'	-98.2(4)
N1	C2'	C3'	C4'	117.7(5)	C8"	C9"	C3"	C12'	141.4(4)
C3	O1	C2'	N1	-118.7(4)	C8"	C9"	C4"	C5"	-0.8(7)
C3	O1	C2'	C3"	131.9(4)	C8"	C7"	C6"	C5"	-0.5(7)
C3	O1	C2'	C3'	6.3(6)	C7	C8	C14	C13	-178.6(3)
C3	C4	C5	C6	-175.6(4)	C7	C8	C14	C15	-56.1(4)
C3	C4	C5	C10	2.8(6)	C7	C8	C9	C11	-179.2(3)
C3	C2	C4'	C3'	1.7(7)	C7	C8	C9	C10	53.7(4)
C8	C14	C13	C12	-61.2(4)	C1	C2	C4'	C3'	-178.3(5)
C8	C14	C13	C17	178.7(3)	C2'	O1	C3	C4	177.8(4)
C8	C14	C13	C18	62.5(4)	C2'	O1	C3	C2	-4.4(7)
C8	C14	C15	C16	-159.1(3)	C2'	N1	C8"	C9"	-21.1(4)
C8	C9	C10	C5	-19.1(5)	C2'	N1	C8"	C7"	160.6(4)
C8	C9	C10	C1	162.4(3)	C2	C3	C4	C5	0.3(6)
C14	C8	C9	C11	-55.2(4)	C2	C1	C10	C9	-179.4(4)
C14	C8	C9	C10	177.7(3)	C2	C1	C10	C5	2.1(6)
C14	C8	C7	C6	172.4(3)	C2	C4'	C3'	C2'	0.7(8)
C14	C13	C17	O2	-165.7(3)	C10	C5	C6	C7	-8.1(5)
C14	C13	C17	C16	-44.5(3)	C10	C1	C2	C3	0.9(6)
C14	C15	C16	C17	4.1(4)	C10	C1	C2	C4'	-179.0(4)
C12	C11	C9	C8	53.6(4)	C9"	C8"	C7"	C6"	-0.7(7)
C12	C11	C9	C10	179.4(3)	C3"	C2'	C3'	C4'	-125.7(5)
C12	C13	C17	O2	79.4(4)	C3"	C9"	C4"	C5"	-176.7(4)

Table S4. Torsion Angles for SPP (**3**).

A	B	C	D	Angle/°	A	B	C	D	Angle/°
C12	C13	C17	C16	-159.5(3)	C4"	C9"	C3"	C2'	-163.8(5)
C11	C12	C13	C14	56.0(4)	C4"	C9"	C3"	C11'	78.1(6)
C11	C12	C13	C17	165.4(3)	C4"	C9"	C3"	C12'	-42.3(7)
C11	C12	C13	C18	-69.4(4)	C4"	C5"	C6"	C7"	1.0(7)
C11	C9	C10	C5	-145.4(4)	C3'	C2'	C3"	C9"	-153.5(4)
C11	C9	C10	C1	36.1(5)	C3'	C2'	C3"	C11'	-38.5(5)
C9	C8	C14	C13	60.5(4)	C3'	C2'	C3"	C12'	83.8(5)
C9	C8	C14	C15	-177.0(3)	C7"	C8"	C9"	C3"	178.1(4)
C9	C8	C7	C6	-67.0(4)	C7"	C8"	C9"	C4"	1.3(7)
C13	C14	C15	C16	-32.5(4)	C6"	C5"	C4"	C9"	-0.3(7)
C13	C12	C11	C9	-54.0(4)	C10'	N1	C8"	C9"	-158.3(4)
C13	C17	C16	C15	25.5(4)	C10'	N1	C8"	C7"	23.5(7)
C4	C3	C2	C1	-2.2(6)	C10'	N1	C2'	O1	59.0(5)
C4	C3	C2	C4'	177.8(4)	C10'	N1	C2'	C3"	170.8(4)
C4	C5	C6	C7	170.2(4)	C10'	N1	C2'	C3'	-66.1(5)
C4	C5	C10	C9	177.5(3)					

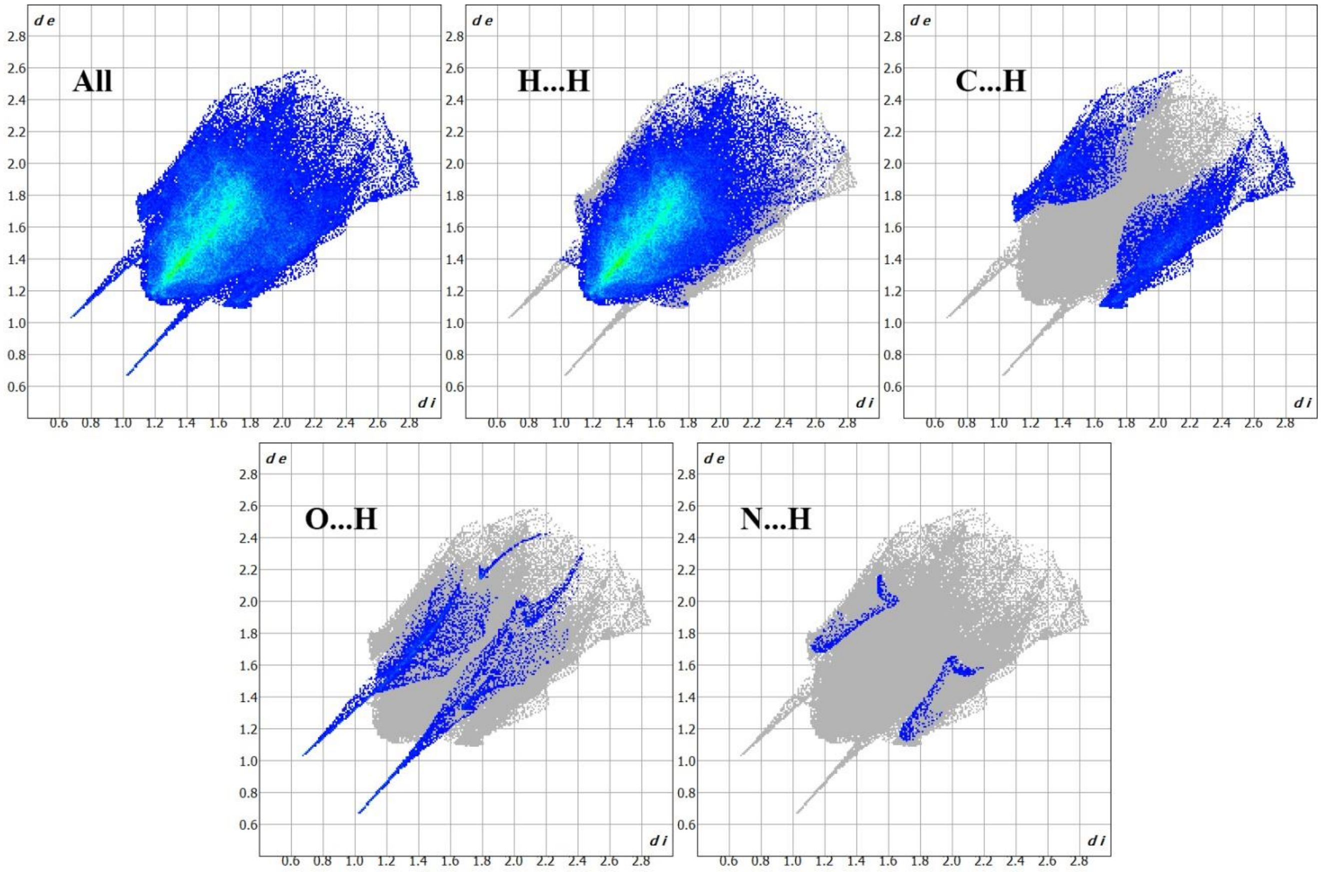

Figure S38. 2D fingerprint plot for SPP (**3**).

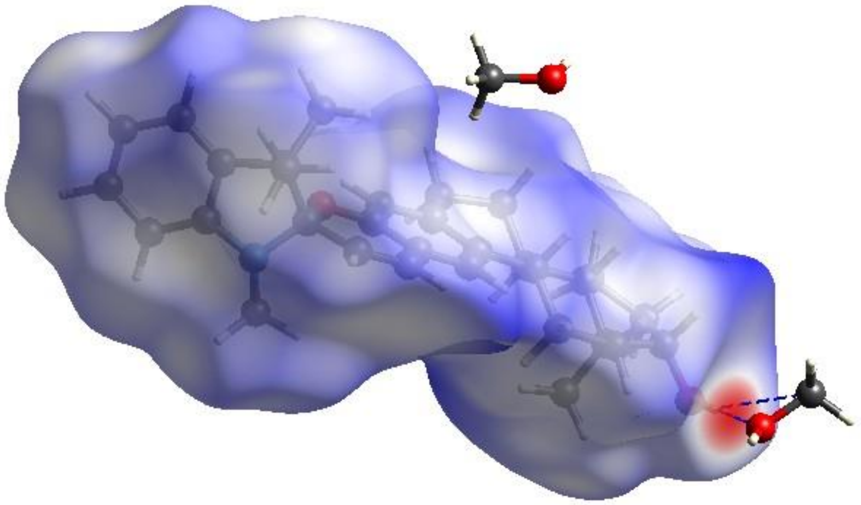

Figure S39. Classical hydrogen bond in the crystal of compound (**3**).

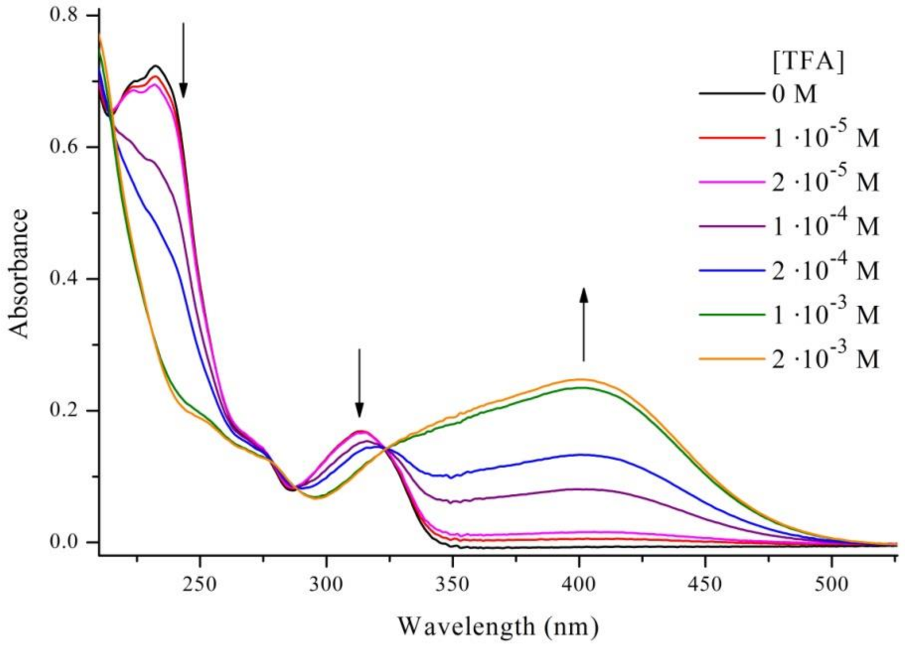

Figure S40. Changes in the UV-Vis spectra of SPP (**4**) in acetonitrile (C = 2 · 10^{-5} M, l = 1cm, T = 293 K) under increasing trifluoroacetic acid (TFA) concentration.

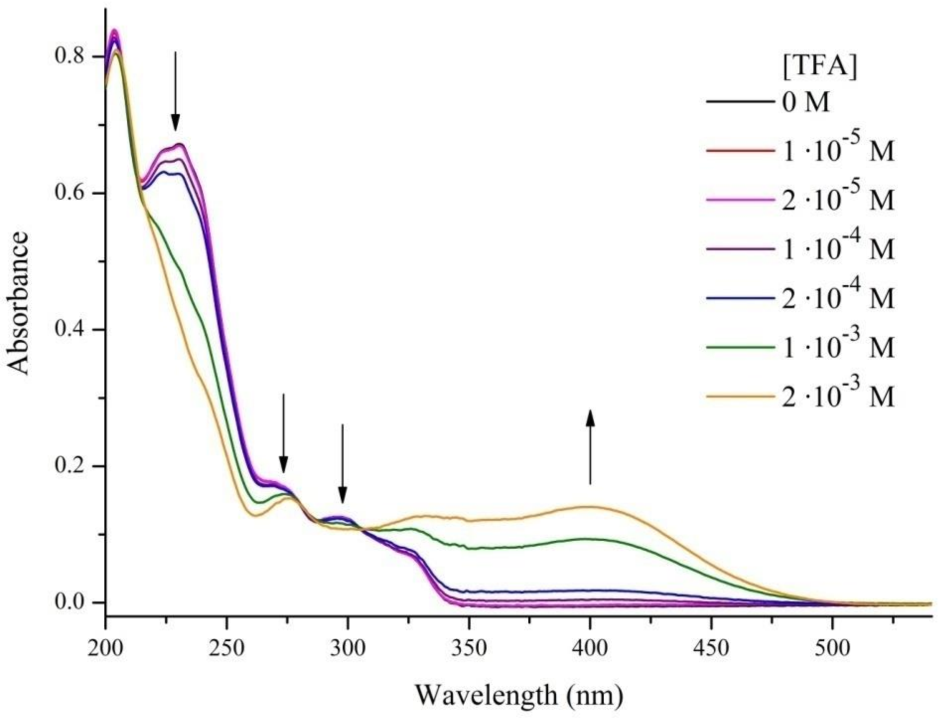

Figure S41. Changes in the UV-Vis spectra of SPP (**5**) in acetonitrile ($C = 2 \cdot 10^{-5}$ M, l = 1cm, T = 293 K) under increasing TFA concentration.

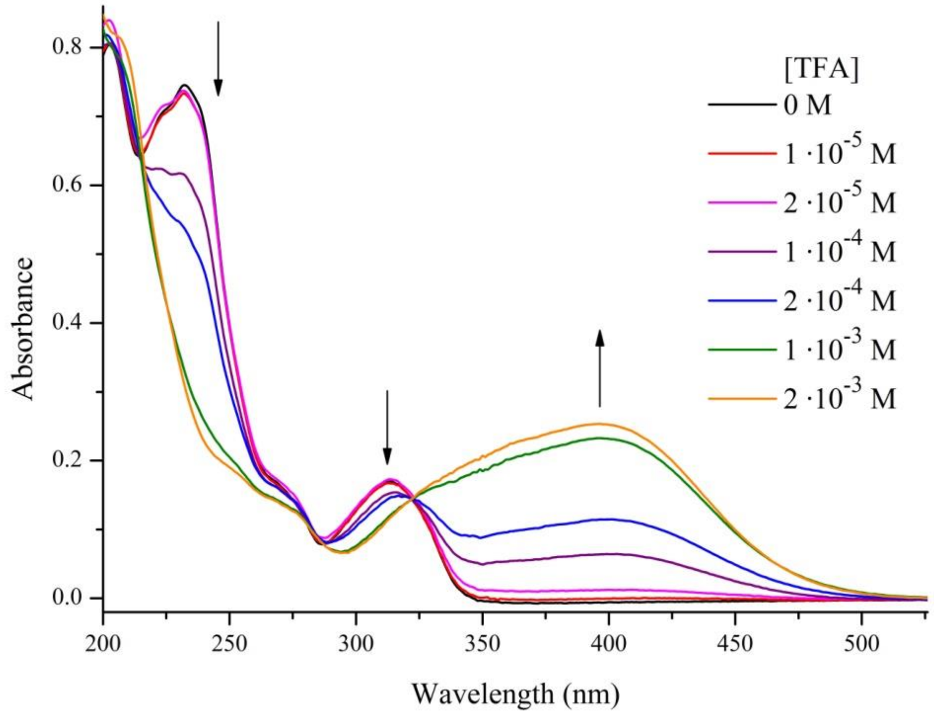

Figure S42. Changes in the UV-Vis spectra of SPP (**6**) in acetonitrile ($C = 2 \cdot 10^{-5}$ M, l = 1 cm, T = 293 K) under increasing TFA concentration.

Table S5. Optimized geometry of the cyclic **Sp** form of spiropyran (**3**). The bond lengths and Cartesian coordinates of the atoms are given in Angstroms.

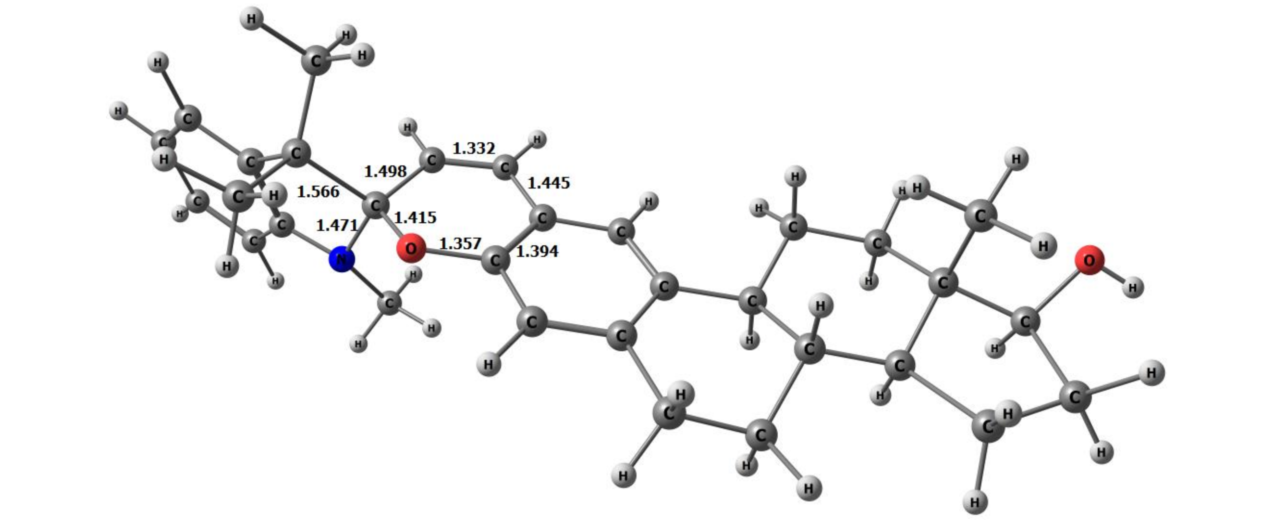

Atom	X	Y	Z	Atom	X	Y	Z
8	-5.152400643	9.031469249	11.508773178	1	-3.723051742	6.997028224	9.741696540
8	4.544763916	5.802900500	7.845037887	1	-3.293872255	8.340837582	8.686466606
6	-4.026689810	9.670651687	10.933437017	1	-2.500595244	8.583569135	12.901431337
6	-3.447071918	10.818546241	11.781630249	1	-3.465757924	7.270041851	12.231030137
6	-1.923145106	10.795488666	11.535253800	1	-1.722918459	7.305333199	11.972805670
6	-1.745838813	9.730336387	10.454057558	1	4.193536379	7.968598073	9.144575074
6	-0.394282377	9.082676224	10.245494616	1	0.026471737	5.822095701	7.654102719
6	0.726640851	10.073588553	9.992497432	6	3.468154730	4.025349976	6.586105208
6	2.061018197	9.352621632	9.970692261	6	4.735534102	4.751460947	6.917446373
6	2.044772598	8.089446106	9.152562715	6	5.861030575	3.797693920	7.441803946
6	3.265945355	7.504625230	8.824511910	7	5.329786648	5.290028517	5.684832594
6	3.320434490	6.334130012	8.092925684	6	6.478122332	3.367252570	6.134225272
6	2.143498943	5.719522605	7.667146825	6	6.146600694	4.309414627	5.153354606
6	0.929848627	6.312666175	7.998493809	6	5.328552205	2.664900434	8.301718977
6	0.846273588	7.488256783	8.738320191	6	6.905954302	4.592205173	8.231185788
6	-0.495351520	8.118229011	9.056284629	6	4.544243111	6.143233044	4.830173306
6	-1.624976509	7.102347056	9.242663809	6	7.297997090	2.304223490	5.832959147
6	-2.964305579	7.765232265	9.560486423	6	6.619723952	4.183964275	3.854836148
6	-2.853356074	8.710534899	10.747252542	6	7.788558563	2.167884073	4.530193983
6	-2.615772500	7.927002853	12.037132389	6	7.450303332	3.101845593	3.562249758
6	2.264385272	4.495619160	6.908138837	1	3.585964164	3.122351684	5.998475644
1	-5.881989729	9.658207299	11.509578386	1	1.360214243	3.977250190	6.604638082
1	-4.300096566	10.053236344	9.940292742	1	7.556229988	1.574594272	6.593741277
1	-3.686673312	10.636652773	12.832028451	1	8.426338030	1.329378400	4.276100280
1	-3.897377715	11.775093418	11.508746746	1	7.827566444	2.987128625	2.551788290
1	-1.384812452	10.514413536	12.444919116	1	6.351829050	4.898001924	3.085262892
1	-1.536586302	11.766705098	11.222510896	1	4.015302052	6.874696265	5.441082855
1	-2.017044517	10.199614093	9.495167551	1	5.206074275	6.689682712	4.156397573
1	-0.131301419	8.505843390	11.143396090	1	3.811938990	5.590466542	4.227663405
1	0.739794797	10.847392236	10.764307698	1	7.265380618	5.449508452	7.659754634
1	0.548810515	10.579144600	9.035221914	1	7.753896880	3.939475213	8.448105934
1	2.339178463	9.089526513	10.999215494	1	6.493254382	4.951939503	9.174931178
1	2.856002061	10.008932444	9.604965870	1	6.159064739	2.055872601	8.665642276
1	-0.774917969	8.743686377	8.193204476	1	4.647008205	2.014475520	7.751989194
1	-1.346944300	6.402751878	10.038005856	1	4.800227474	3.064821711	9.170993724
1	-1.743882922	6.504028679	8.336730201				

Table S6. Optimized geometry of **TS**(**Sp-CCC**) of spiropyran (**3**). The bond lengths and Cartesian coordinates of the atoms are given in Angstroms.

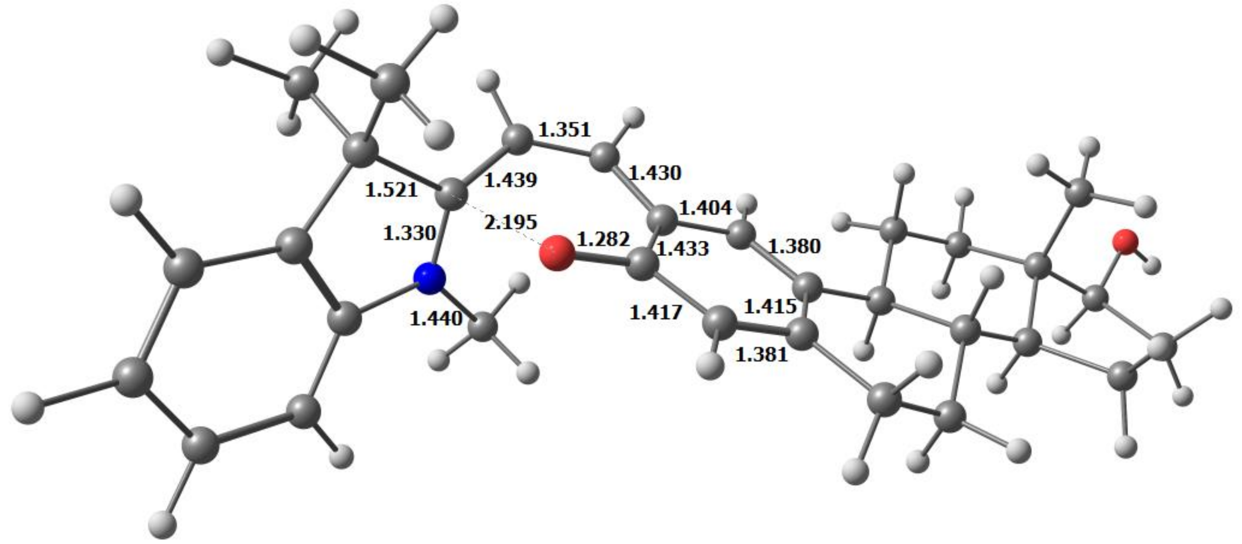

ν_{im} = -143.24 cm^{-1}

Atom	X	Y	Z	Atom	X	Y	Z
8	-4.957994000	9.469773000	11.497834000	1	-3.664167000	7.207518000	9.926209000
8	4.520842000	5.469074000	8.051262000	1	-3.217160000	8.408819000	8.716905000
6	-3.829010000	10.000129000	10.826175000	1	-2.228916000	9.086589000	12.841375000
6	-3.181680000	11.206693000	11.529278000	1	-3.306752000	7.768301000	12.383507000
6	-1.673256000	11.113924000	11.214841000	1	-1.582145000	7.659778000	12.038649000
6	-1.568332000	9.922719000	10.262051000	1	4.268702000	7.837334000	9.043879000
6	-0.244521000	9.210473000	10.092957000	1	0.018183000	5.545967000	8.149314000
6	0.884324000	10.128876000	9.660229000	6	3.093241000	3.234488000	7.250658000
6	2.206859000	9.384458000	9.676552000	6	4.368160000	3.695905000	6.767165000
6	2.140757000	8.005155000	9.072522000	6	5.674663000	2.981506000	7.077549000
6	3.326062000	7.341508000	8.826186000	7	4.529713000	4.424798000	5.666825000
6	3.394012000	6.036998000	8.277736000	6	6.629129000	3.723525000	6.182540000
6	2.134780000	5.394841000	8.044247000	6	5.891312000	4.546901000	5.340854000
6	0.944277000	6.082324000	8.328961000	6	5.548007000	1.528280000	6.576443000
6	0.900966000	7.374927000	8.811489000	6	6.048650000	2.984526000	8.551361000
6	-0.408314000	8.100947000	9.045287000	6	3.501483000	5.167800000	4.985509000
6	-1.579821000	7.168281000	9.361225000	6	8.001020000	3.668868000	6.061228000
6	-2.881832000	7.917699000	9.638929000	6	6.474501000	5.328611000	4.362887000
6	-2.695230000	8.982494000	10.708472000	6	8.616377000	4.454719000	5.086621000
6	-2.432046000	8.341373000	12.070135000	6	7.861737000	5.268162000	4.250300000
6	2.081281000	4.007723000	7.701216000	1	2.970726000	2.155727000	7.252636000
1	-5.666563000	10.117890000	11.444121000	1	1.143804000	3.501147000	7.920007000
1	-4.121270000	10.285903000	9.806093000	1	8.592324000	3.031627000	6.709577000
1	-3.371532000	11.131021000	12.602626000	1	9.694746000	4.432367000	4.981376000
1	-3.622445000	12.146115000	11.189524000	1	8.357794000	5.872375000	3.499611000
1	-1.095066000	10.930894000	12.125006000	1	5.889319000	5.970382000	3.716168000
1	-1.283906000	12.030440000	10.768692000	1	2.536437000	4.714442000	5.197904000
1	-1.858348000	10.283322000	9.262552000	1	3.495166000	6.204634000	5.330517000
1	0.040672000	8.752779000	11.050711000	1	3.692063000	5.142921000	3.913031000
1	0.947991000	11.001689000	10.315636000	1	6.164832000	4.001246000	8.919159000
1	0.666935000	10.502746000	8.651826000	1	6.982881000	2.435682000	8.687166000
1	2.536990000	9.278313000	10.717848000	1	5.271374000	2.488445000	9.136251000
1	2.987822000	9.963746000	9.175288000	1	6.522027000	1.044783000	6.665097000
1	-0.672765000	8.625621000	8.112036000	1	5.235163000	1.491653000	5.531117000
1	-1.314401000	6.536636000	10.215655000	1	4.831517000	0.972604000	7.183504000
1	-1.746935000	6.487788000	8.522746000				

Table S7. Optimized geometry of the open **CCC** form of spiropyran (**3**). The bond lengths and Cartesian coordinates of the atoms are given in Angstroms, the torsion angles - in degrees.

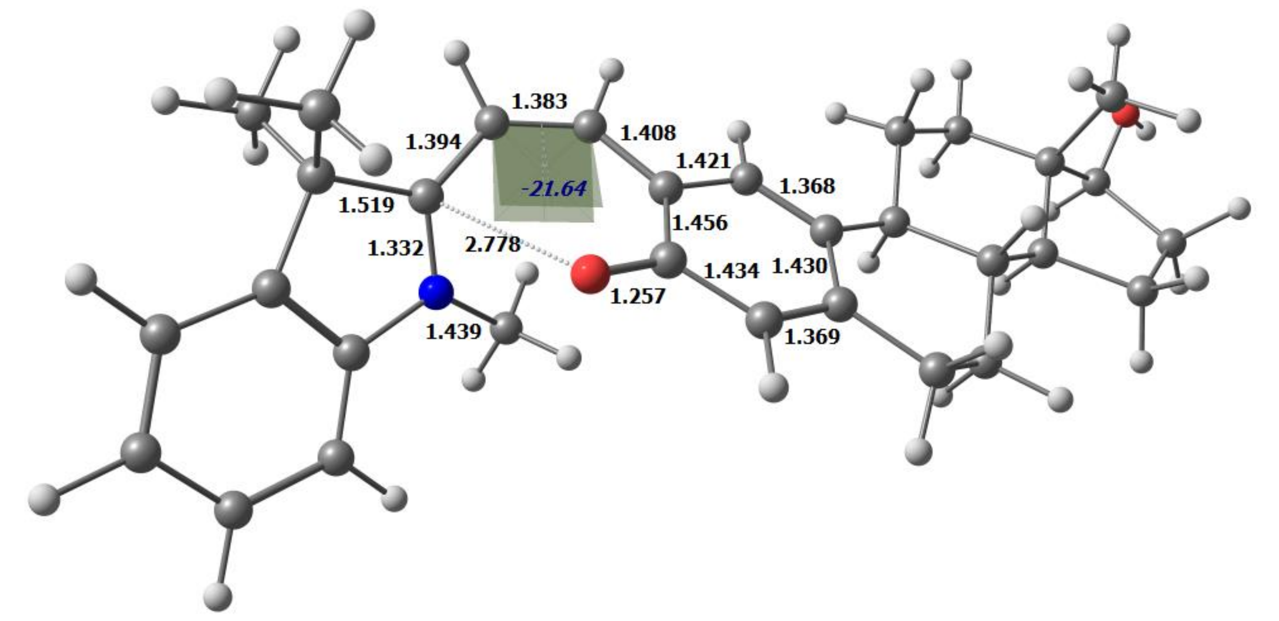

Atom	X	Y	Z	Atom	X	Y	Z
8	-5.095705882	9.386092329	11.381040296	1	-3.551742551	7.000981055	10.269815343
8	4.748925503	5.787577796	8.450003201	1	-3.202714352	8.009690828	8.868712011
6	-3.991549809	9.890905199	10.651285804	1	-2.392657654	9.480958283	12.822490800
6	-3.469467117	11.250874992	11.149518962	1	-3.327424056	8.006296519	12.578124125
6	-1.948194343	11.236641566	10.886611625	1	-1.588043316	8.009342186	12.288058577
6	-1.718522274	9.919594942	10.145343756	1	4.291159809	8.210161279	9.175621152
6	-0.335286975	9.306892661	10.100952149	1	0.243914717	5.497608368	8.510244496
6	0.728530024	10.241682695	9.555070257	6	3.306916000	3.303656000	7.464132000
6	2.098696512	9.610769611	9.711984679	6	4.483591771	3.588375050	6.773409334
6	2.166577874	8.185263163	9.232354064	6	5.684748279	2.659112412	6.748597458
6	3.393314995	7.618250622	9.016693550	7	4.713871050	4.636724722	5.984914601
6	3.594834361	6.265330297	8.586496742	6	6.645599469	3.440321746	5.895085104
6	2.373399584	5.503842397	8.364039599	6	6.020498490	4.605519522	5.470042730
6	1.124352505	6.115147002	8.655826088	6	5.322164123	1.321499277	6.092809780
6	0.974427139	7.416007322	9.052449903	6	6.220530898	2.431297682	8.162733703
6	-0.392852050	8.044467747	9.230364480	6	3.734222973	5.592478714	5.540238700
6	-1.464670078	7.067937649	9.718050422	6	7.939649670	3.171319430	5.501689980
6	-2.837008312	7.723771037	9.862880976	6	6.646598722	5.534998997	4.660712228
6	-2.773499230	8.971473952	10.730789654	6	8.595014903	4.093486894	4.686740497
6	-2.498718731	8.599736581	12.187249787	6	7.955091911	5.257110664	4.275578108
6	2.344170000	4.147093000	7.988796000	1	3.130027284	2.241338950	7.609363766
1	-5.843402340	9.971902762	11.229728367	1	1.405716487	3.647234722	8.226240340
1	-4.271196795	9.973522970	9.591663969	1	8.439816884	2.263567830	5.821317504
1	-3.689862221	11.342710952	12.215830064	1	9.614671077	3.903587649	4.372755210
1	-3.972768023	12.077215202	10.643620571	1	8.481747861	5.964483012	3.645662293
1	-1.389347518	11.253833828	11.826413111	1	6.153618507	6.445443591	4.343790713
1	-1.617886336	12.097879158	10.303745459	1	2.749547680	5.131142215	5.582865358
1	-2.016928296	10.086200716	9.098417852	1	3.731304908	6.485062079	6.168250602
1	-0.036786912	9.020319858	11.119254174	1	3.962991039	5.870304630	4.512381184
1	0.705531958	11.201807212	10.077401304	1	6.463457994	3.379156060	8.644325880
1	0.517423972	10.446834507	8.498129236	1	7.121749598	1.816565230	8.117365880
1	2.364543453	9.619686838	10.776912434	1	5.474680073	1.912570481	8.768157515
1	2.868881737	10.199231524	9.205866399	1	6.219608847	0.704126855	6.019392952
1	-0.718359127	8.397205560	8.237674411	1	4.918387096	1.470784517	5.089790573
1	-1.144097992	6.635620793	10.671859575	1	4.585617293	0.785847074	6.694468583
1	-1.556209236	6.232260891	9.020640350				

Table S8. Optimized geometry of **TS**(**CCC-CTC**) of spiropyran (**3**). The bond lengths and Cartesian coordinates of the atoms are given in Angstroms, the torsion angles - in degrees.

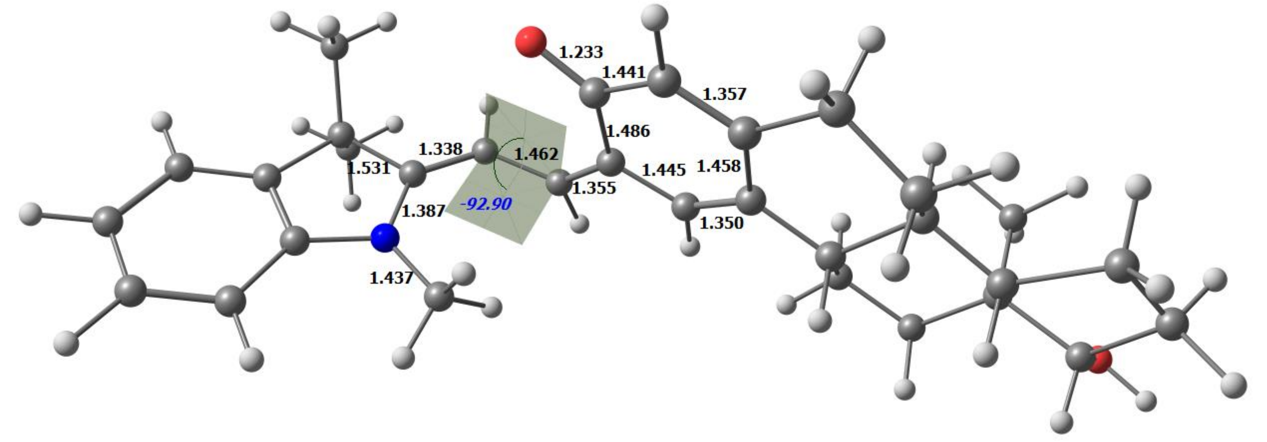

ν_{im} = -242.58 cm^{-1}

Atom	X	Y	Z	Atom	X	Y	Z
8	-5.332850000	9.347047000	10.654769000	1	-3.588192000	7.110226000	9.531016000
8	4.901977000	5.941544000	9.529456000	1	-2.952588000	8.297850000	8.395535000
6	-4.110032000	9.921727000	10.229584000	1	-2.980346000	9.181628000	12.598586000
6	-3.705847000	11.192501000	10.998868000	1	-3.834707000	7.773816000	11.968450000
6	-2.162951000	11.182295000	11.042981000	1	-2.072330000	7.782276000	12.036830000
6	-1.780739000	9.966978000	10.198157000	1	4.322135000	8.335122000	10.263266000
6	-0.414676000	9.338212000	10.365711000	1	0.489679000	5.712399000	8.532011000
6	0.726722000	10.316099000	10.159314000	6	3.927470000	3.694707000	7.984370000
6	2.042919000	9.653251000	10.512960000	6	4.752557000	3.683307000	6.930881000
6	2.234818000	8.295915000	9.900482000	6	5.972394000	2.765029000	6.823714000
6	3.483142000	7.765049000	9.875768000	7	4.700769000	4.471212000	5.790954000
6	3.773585000	6.427611000	9.424378000	6	6.604333000	3.248703000	5.541448000
6	2.615432000	5.686261000	8.861398000	6	5.809686000	4.256479000	4.991352000
6	1.309176000	6.299661000	8.930591000	6	5.540487000	1.302225000	6.705571000
6	1.088709000	7.545472000	9.402137000	6	6.915068000	2.942750000	8.011659000
6	-0.279787000	8.192244000	9.352325000	6	3.700260000	5.464181000	5.512943000
6	-1.433799000	7.192072000	9.445466000	6	7.760715000	2.849995000	4.910214000
6	-2.805444000	7.862851000	9.391891000	6	6.168013000	4.890026000	3.809468000
6	-2.927015000	8.974022000	10.423547000	6	8.133885000	3.469736000	3.714109000
6	-2.949142000	8.398710000	11.838660000	6	7.342071000	4.477257000	3.181001000
6	2.728426000	4.496942000	8.222093000	1	4.162050000	2.987614000	8.778161000
1	-6.039793000	9.962945000	10.440013000	1	1.784616000	4.072938000	7.872175000
1	-4.174881000	10.150490000	9.156578000	1	8.377613000	2.066027000	5.337857000
1	-4.134398000	11.147221000	12.002934000	1	9.043414000	3.168774000	3.207750000
1	-4.103284000	12.088455000	10.517900000	1	7.640006000	4.961231000	2.257286000
1	-1.802878000	11.068328000	12.069372000	1	5.568855000	5.685425000	3.383740000
1	-1.729139000	12.104322000	10.652873000	1	2.713402000	5.089162000	5.782341000
1	-1.854489000	10.273714000	9.142976000	1	3.881989000	6.396293000	6.057905000
1	-0.329984000	8.929775000	11.382282000	1	3.692789000	5.678277000	4.444875000
1	0.591615000	11.204978000	10.780091000	1	7.221234000	3.985444000	8.114578000
1	0.729829000	10.650582000	9.114750000	1	7.809132000	2.330369000	7.872023000
1	2.077218000	9.518003000	11.601886000	1	6.427764000	2.631717000	8.938482000
1	2.898529000	10.285295000	10.262290000	1	6.415668000	0.664150000	6.559761000
1	-0.358103000	8.678551000	8.366269000	1	4.864632000	1.162129000	5.859399000
1	-1.327577000	6.612860000	10.368462000	1	5.027762000	0.984105000	7.616490000
1	-1.372361000	6.473161000	8.625436000				

Table S9. Optimized geometry of the open **CTC** form of spiropyran (**3**). The bond lengths and Cartesian coordinates of the atoms are given in Angstroms, the torsion angles - in degrees.

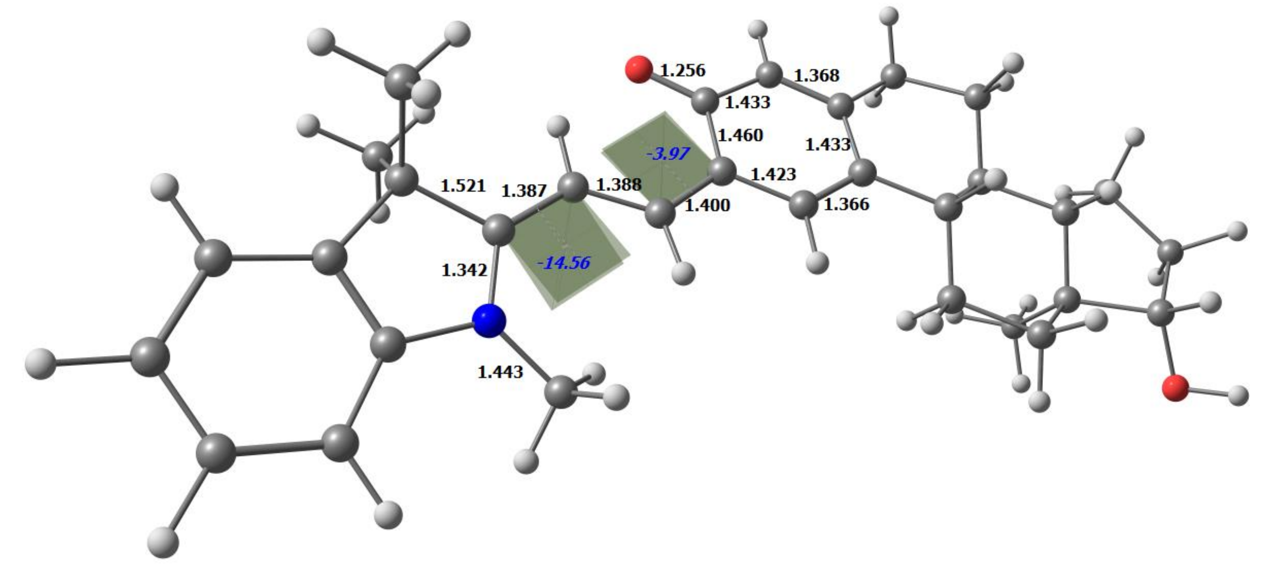

Atom	X	Y	Z	Atom	X	Y	Z
8	-5.072607000	10.094933000	9.187712000	1	-2.858945000	8.510094000	7.794996000
8	4.789714000	5.660353000	10.504050000	1	-1.837837000	9.931181000	8.000240000
6	-3.832005000	10.489321000	9.747593000	1	-3.934337000	8.313373000	11.379875000
6	-3.924173000	10.979093000	11.204903000	1	-4.290330000	7.752746000	9.746576000
6	-2.601396000	10.553042000	11.876371000	1	-2.783782000	7.283736000	10.533612000
6	-1.781715000	9.967964000	10.727369000	1	3.640741000	7.114277000	12.283582000
6	-0.625850000	9.037446000	11.023510000	1	1.352108000	7.066205000	7.924582000
6	0.400165000	9.621733000	11.976244000	6	4.631989000	4.896071000	7.736575000
6	1.418871000	8.561439000	12.347620000	6	5.094598000	4.208400000	6.624198000
6	1.976147000	7.816957000	11.164945000	6	6.534229000	3.722944000	6.545323000
6	3.132495000	7.103040000	11.322998000	7	4.446521000	3.844638000	5.506543000
6	3.734536000	6.308648000	10.294042000	6	6.564691000	3.073463000	5.191200000
6	3.028053000	6.318475000	9.016789000	6	5.305525000	3.180683000	4.614313000
6	1.833454000	7.082070000	8.897250000	6	6.828790000	2.715947000	7.661130000
6	1.289576000	7.823737000	9.907233000	6	7.502052000	4.906885000	6.622545000
6	0.058562000	8.681421000	9.697486000	6	3.038101000	4.009876000	5.240372000
6	-0.931676000	8.104197000	8.683541000	6	7.586242000	2.443711000	4.512996000
6	-2.155526000	8.996330000	8.478755000	6	5.025729000	2.682200000	3.354443000
6	-2.828394000	9.337815000	9.799657000	6	7.327797000	1.927752000	3.243951000
6	-3.492230000	8.102093000	10.404453000	6	6.064698000	2.048791000	2.677628000
6	3.459935000	5.626051000	7.879465000	1	5.306789000	4.911211000	8.582113000
1	-5.619837000	10.880651000	9.095723000	1	2.806941000	5.735010000	7.020135000
1	-3.393115000	11.280443000	9.123873000	1	8.572048000	2.352308000	4.955919000
1	-4.786167000	10.506404000	11.681879000	1	8.117980000	1.431178000	2.693313000
1	-4.083813000	12.058408000	11.248242000	1	5.880011000	1.646882000	1.688157000
1	-2.779820000	9.797594000	12.646850000	1	4.048848000	2.777374000	2.897760000
1	-2.091841000	11.387729000	12.360536000	1	2.819976000	5.011120000	4.862599000
1	-1.360362000	10.818511000	10.168938000	1	2.730881000	3.276639000	4.498231000
1	-1.016007000	8.116705000	11.479488000	1	2.473758000	3.837103000	6.156573000
1	-0.082484000	9.998058000	12.882009000	1	7.289676000	5.637079000	5.839438000
1	0.891982000	10.477235000	11.496364000	1	8.525627000	4.548229000	6.496966000
1	0.930622000	7.827387000	13.001772000	1	7.421736000	5.400428000	7.592827000
1	2.242286000	8.982454000	12.930727000	1	7.842929000	2.330410000	7.539073000
1	0.402423000	9.645556000	9.287305000	1	6.130652000	1.877658000	7.627575000
1	-1.236770000	7.105570000	9.013856000	1	6.757095000	3.195465000	8.638865000
1	-0.441348000	7.967794000	7.717074000				

Table S10. Optimized geometry of **TS**(**CTC-TTC**) of spiropyran (**3**). The bond lengths and Cartesian coordinates of the atoms are given in Angstroms, the torsion angles - in degrees.

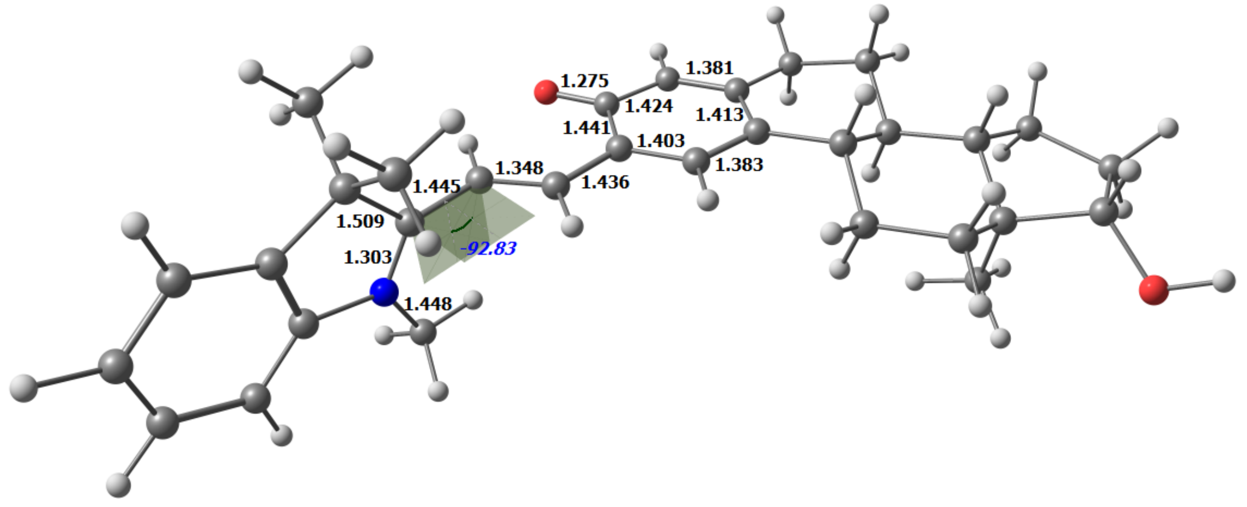

ν_{im} = -169.95 cm^{-1}

Atom	X	Y	Z	Atom	X	Y	Z
8	-4.269112000	11.181538000	8.682946000	1	-2.015616000	9.559808000	7.442753000
8	4.155744000	4.592276000	10.799606000	1	-0.900252000	10.634413000	8.282410000
6	-3.203316000	11.147017000	9.615495000	1	-4.081410000	8.718647000	10.441158000
6	-3.640943000	11.255306000	11.087988000	1	-3.986941000	8.630879000	8.683674000
6	-2.626457000	10.410456000	11.889177000	1	-2.863191000	7.703631000	9.676834000
6	-1.599662000	9.982501000	10.840404000	1	2.799735000	5.803082000	12.646118000
6	-0.693358000	8.798237000	11.101744000	1	1.711401000	7.222795000	8.075607000
6	0.117368000	8.924849000	12.379367000	6	4.453311000	4.421363000	8.004108000
6	0.874773000	7.638243000	12.652015000	6	5.009305000	3.924312000	6.766083000
6	1.592487000	7.089131000	11.445082000	6	6.225924000	4.480404000	6.067261000
6	2.558050000	6.119313000	11.633410000	7	4.560443000	2.883369000	6.123112000
6	3.271332000	5.483441000	10.577575000	6	6.367431000	3.533759000	4.910391000
6	2.908723000	5.924481000	9.254066000	6	5.349610000	2.594901000	4.980773000
6	1.933378000	6.920069000	9.094858000	6	7.428776000	4.439679000	7.016259000
6	1.261447000	7.523774000	10.141715000	6	5.947382000	5.917957000	5.612909000
6	0.265867000	8.643925000	9.913805000	6	3.413471000	2.079107000	6.491487000
6	-0.487039000	8.545618000	8.586196000	6	7.281416000	3.467998000	3.878163000
6	-1.465950000	9.699220000	8.379586000	6	5.185429000	1.574791000	4.066700000
6	-2.422975000	9.835499000	9.553871000	6	7.143609000	2.448359000	2.939395000
6	-3.390838000	8.654154000	9.598199000	6	6.113719000	1.517412000	3.032542000
6	3.504923000	5.377256000	8.067399000	1	4.842075000	4.010687000	8.934749000
1	-4.673766000	12.053016000	8.727331000	1	3.127322000	5.784087000	7.129674000
1	-2.499186000	11.958540000	9.384656000	1	8.087723000	4.188036000	3.797745000
1	-4.655390000	10.860725000	11.185426000	1	7.851189000	2.377498000	2.121702000
1	-3.667133000	12.296355000	11.416336000	1	6.030725000	0.734008000	2.288861000
1	-3.111156000	9.536458000	12.333348000	1	4.382228000	0.852791000	4.143195000
1	-2.171069000	10.972622000	12.705990000	1	2.692227000	2.096185000	5.674187000
1	-0.937454000	10.846456000	10.672660000	1	3.740965000	1.053730000	6.664686000
1	-1.304140000	7.887520000	11.181192000	1	2.972022000	2.495009000	7.392611000
1	-0.531964000	9.156643000	13.228273000	1	5.076328000	5.965519000	4.957035000
1	0.815995000	9.764874000	12.274575000	1	6.817589000	6.285245000	5.066831000
1	0.159384000	6.879877000	12.995814000	1	5.777582000	6.558827000	6.478879000
1	1.587048000	7.769430000	13.471839000	1	8.307433000	4.809982000	6.485934000
1	0.829956000	9.591378000	9.877341000	1	7.627983000	3.422962000	7.359116000
1	-1.012983000	7.585717000	8.542119000	1	7.243996000	5.078117000	7.881574000
1	0.221683000	8.542461000	7.754926000				

Table S11. Optimized geometry of the open **TTC** form of spiropyran (**3**). The bond lengths and Cartesian coordinates of the atoms are given in Angstroms.

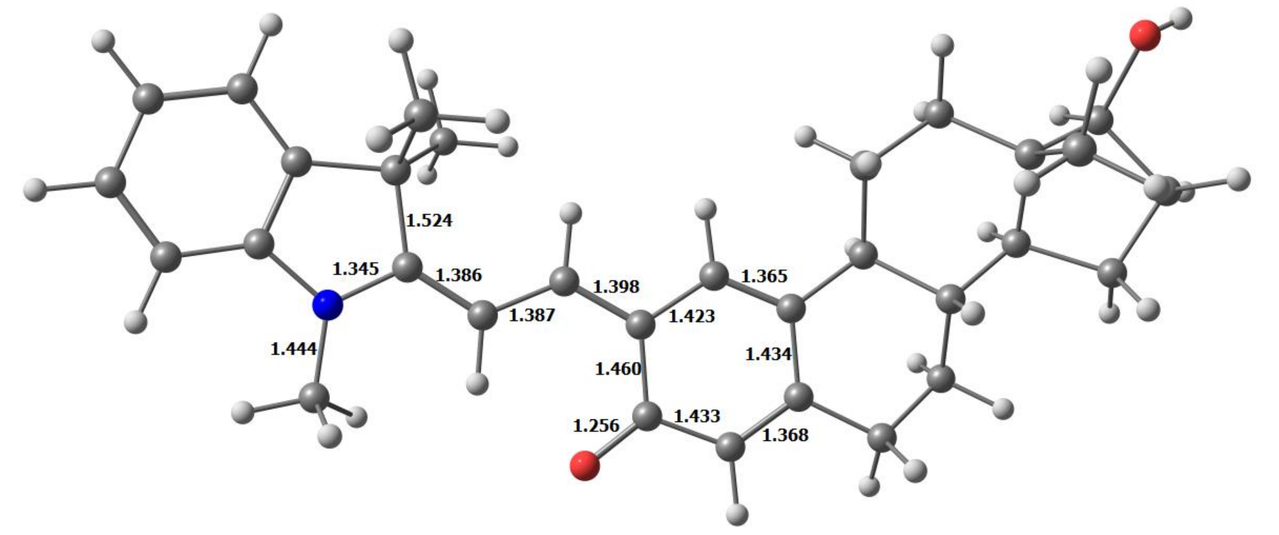

Atom	X	Y	Z	Atom	X	Y	Z
8	-7.040011000	3.065344000	12.136719000	1	-4.395015000	2.369887000	10.797050000
8	2.686598000	6.987821000	9.217335000	1	-4.827822000	3.531528000	9.545414000
6	-6.553228000	4.239293000	11.511440000	1	-4.960769000	4.702654000	13.675962000
6	-6.998953000	5.549936000	12.185412000	1	-4.751741000	3.011169000	13.225863000
6	-5.836322000	6.543987000	11.976764000	1	-3.429054000	4.155097000	13.003853000
6	-4.850041000	5.773676000	11.098808000	1	0.792638000	8.462741000	10.141254000
6	-3.399530000	6.199895000	11.041809000	1	-0.546868000	3.828067000	9.150147000
6	-3.210630000	7.650031000	10.635564000	6	3.274643000	4.314318000	8.332247000
6	-1.750982000	8.040941000	10.765932000	6	4.054216000	3.270801000	7.858141000
6	-0.795783000	7.050258000	10.156952000	6	3.677907000	1.820817000	7.580467000
6	0.494577000	7.440693000	9.922162000	7	5.356132000	3.447950000	7.571496000
6	1.515844000	6.576390000	9.408873000	6	4.992819000	1.262369000	7.097588000
6	1.083274000	5.208029000	9.139693000	6	5.952426000	2.267053000	7.114079000
6	-0.272927000	4.852028000	9.383301000	6	2.619980000	1.705265000	6.477146000
6	-1.216898000	5.710499000	9.869372000	6	3.238957000	1.094504000	8.857649000
6	-2.665343000	5.299719000	10.038576000	6	6.049574000	4.705703000	7.717572000
6	-2.852504000	3.814306000	10.352015000	6	5.328512000	-0.008010000	6.681337000
6	-4.320931000	3.424618000	10.512617000	6	7.261595000	2.043465000	6.725018000
6	-5.026891000	4.311734000	11.526528000	6	6.641368000	-0.258138000	6.284672000
6	-4.510550000	4.036304000	12.937973000	6	7.590165000	0.756710000	6.308830000
6	1.927328000	4.209267000	8.646391000	1	3.734550000	5.281346000	8.477329000
1	-7.995524000	3.046121000	12.027625000	1	1.444488000	3.247911000	8.502801000
1	-6.871383000	4.243540000	10.459655000	1	4.587883000	-0.800173000	6.662646000
1	-7.173491000	5.357607000	13.246793000	1	6.924423000	-1.250857000	5.955302000
1	-7.938854000	5.912005000	11.763931000	1	8.607290000	0.547756000	5.998304000
1	-5.375422000	6.812064000	12.931564000	1	8.010534000	2.824895000	6.737015000
1	-6.161632000	7.473565000	11.507359000	1	6.012945000	5.038390000	8.756673000
1	-5.230444000	5.829116000	10.066639000	1	7.087204000	4.581963000	7.422800000
1	-2.946294000	6.067856000	12.034548000	1	5.588237000	5.466457000	7.084978000
1	-3.822027000	8.308904000	11.257594000	1	3.992828000	1.198307000	9.640089000
1	-3.552580000	7.778663000	9.601029000	1	3.114008000	0.032521000	8.637485000
1	-1.507623000	8.119092000	11.833422000	1	2.291285000	1.478931000	9.234906000
1	-1.563144000	9.031500000	10.342952000	1	2.485266000	0.652177000	6.222826000
1	-3.167702000	5.482343000	9.073927000	1	2.936841000	2.239413000	5.579557000
1	-2.291383000	3.567632000	11.259584000	1	1.658151000	2.104938000	6.797961000
1	-2.420470000	3.207642000	9.553006000				

Table S12. Optimized geometry of **TS**(**CTC-CTT**) of spiropyran (**3**). The bond lengths and Cartesian coordinates of the atoms are given in Angstroms, the torsion angles - in degrees.

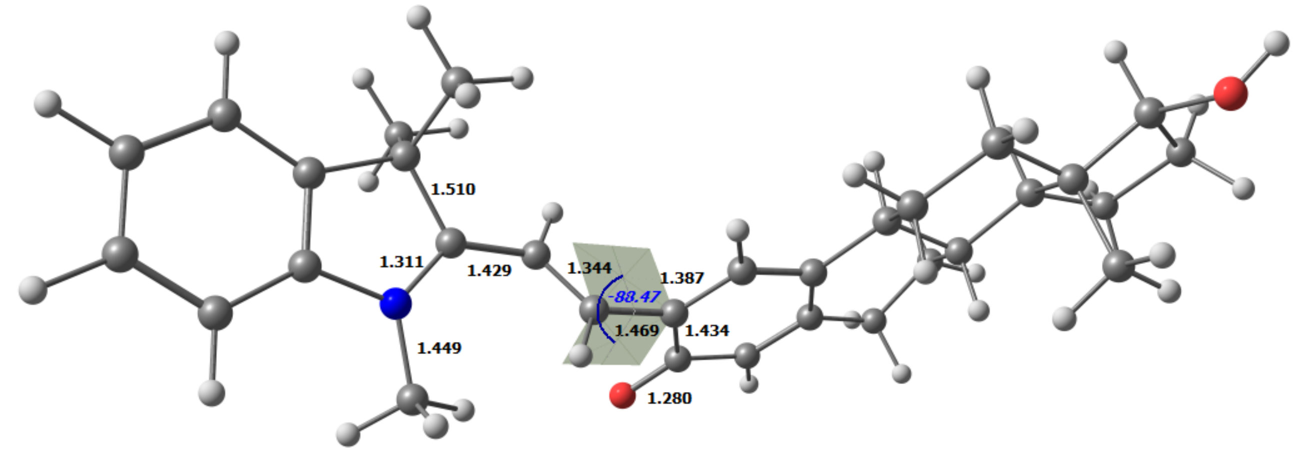

ν_{im} = -160.06 cm^{-1}

Atom	X	Y	Z	Atom	X	Y	Z
8	-3.780716000	11.547760000	8.476330000	1	-1.889380000	9.426723000	7.363056000
8	3.352984000	3.706724000	11.058742000	1	-0.579816000	10.348278000	8.097248000
6	-2.716057000	11.391129000	9.398450000	1	-4.012535000	9.247815000	10.432151000
6	-3.093216000	11.701805000	10.859237000	1	-3.984431000	9.012930000	8.686164000
6	-2.245581000	10.744078000	11.724510000	1	-3.034335000	7.961362000	9.734850000
6	-1.337955000	10.047114000	10.711727000	1	2.170662000	5.229005000	12.832765000
6	-0.669676000	8.735392000	11.063127000	1	1.536240000	6.644408000	8.174310000
6	0.144717000	8.780357000	12.343206000	6	4.310521000	4.756146000	7.906951000
6	0.621907000	7.384534000	12.699746000	6	4.943118000	4.125880000	6.791917000
6	1.244942000	6.653641000	11.536360000	6	5.930569000	4.845537000	5.904698000
6	2.023694000	5.535696000	11.798659000	7	4.777433000	2.891330000	6.381747000
6	2.657053000	4.749188000	10.799738000	6	6.237877000	3.787252000	4.885301000
6	2.446964000	5.229519000	9.465302000	6	5.528293000	2.640539000	5.207075000
6	1.655668000	6.339238000	9.209466000	6	7.178730000	5.234536000	6.709364000
6	1.033202000	7.078194000	10.215379000	6	5.280358000	6.085631000	5.283061000
6	0.238453000	8.327930000	9.896503000	6	4.005795000	1.846867000	7.025073000
6	-0.545498000	8.253015000	8.584478000	6	7.059468000	3.794338000	3.776949000
6	-1.303930000	9.544372000	8.280992000	6	5.586165000	1.478747000	4.463375000
6	-2.196226000	9.955222000	9.442925000	6	7.140340000	2.635810000	3.008469000
6	-3.370619000	8.987849000	9.588601000	6	6.414146000	1.498213000	3.346312000
6	3.070682000	4.497175000	8.355496000	1	4.863971000	5.581635000	8.343292000
1	-3.999322000	12.483368000	8.432639000	1	2.475541000	3.737133000	7.852826000
1	-1.880280000	12.035188000	9.091716000	1	7.627136000	4.677735000	3.507720000
1	-4.162665000	11.521517000	10.992139000	1	7.776934000	2.619881000	2.131732000
1	-2.911207000	12.751337000	11.099374000	1	6.490852000	0.610721000	2.729756000
1	-2.880347000	10.016683000	12.238592000	1	5.016843000	0.595326000	4.722256000
1	-1.676122000	11.269882000	12.492550000	1	3.017960000	1.773240000	6.568817000
1	-0.527697000	10.753371000	10.471562000	1	4.535290000	0.904646000	6.898381000
1	-1.441702000	7.963165000	11.192165000	1	3.907309000	2.075118000	8.084191000
1	-0.447558000	9.192806000	13.164967000	1	4.378497000	5.821762000	4.728310000
1	1.001032000	9.451206000	12.197545000	1	5.990546000	6.550407000	4.597358000
1	-0.235849000	6.802291000	13.060876000	1	5.021283000	6.807316000	6.059338000
1	1.333337000	7.415731000	13.530452000	1	7.905107000	5.680671000	6.028364000
1	0.952220000	9.161894000	9.781087000	1	7.631479000	4.362012000	7.182762000
1	-1.238442000	7.405800000	8.630844000	1	6.928680000	5.968991000	7.476052000
1	0.132375000	8.045611000	7.753371000				

Table S13. Optimized geometry of the open **CTT** form of spiropyran (**3**). The bond lengths and Cartesian coordinates of the atoms are given in Angstroms.

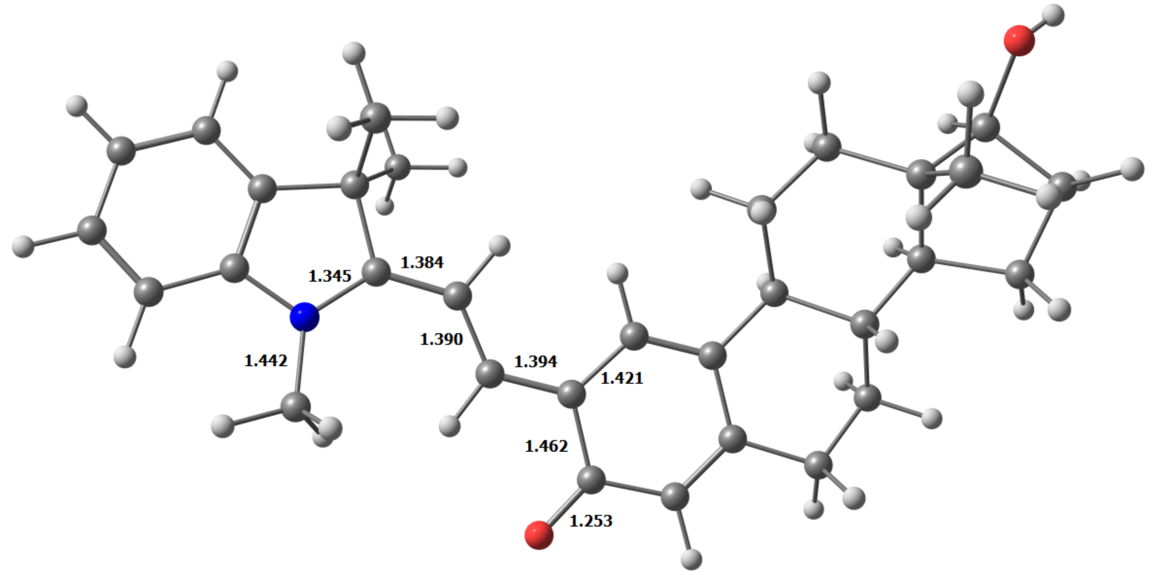

Atom	X	Y	Z	Atom	X	Y	Z
8	-2.611617000	12.075967000	8.239426000	1	-0.445191000	10.090105000	7.439160000
8	2.739901000	3.152437000	11.358909000	1	0.499814000	10.696916000	8.797113000
6	-1.960529000	11.643275000	9.420924000	1	-3.563544000	9.455868000	9.415359000
6	-2.841388000	11.685786000	10.683571000	1	-2.892982000	9.580693000	7.790329000
6	-2.374829000	10.504187000	11.560636000	1	-2.404444000	8.257748000	8.847768000
6	-1.173020000	9.939989000	10.804124000	1	1.197475000	4.487185000	12.951176000
6	-0.712042000	8.524298000	11.074042000	1	2.101035000	6.712517000	8.638446000
6	-0.387344000	8.262426000	12.533329000	6	3.676039000	4.789695000	7.630806000
6	-0.103532000	6.787818000	12.745771000	6	4.489849000	4.198153000	6.680288000
6	0.836766000	6.190362000	11.734287000	6	4.932722000	4.958728000	5.437811000
6	1.401483000	4.975686000	12.002088000	7	5.009486000	2.958442000	6.645735000
6	2.250762000	4.272518000	11.083616000	6	5.809616000	3.946180000	4.756855000
6	2.487847000	4.956494000	9.813466000	6	5.826187000	2.782760000	5.516345000
6	1.894411000	6.226677000	9.583903000	6	5.717275000	6.215978000	5.820331000
6	1.095224000	6.861685000	10.488776000	6	3.725569000	5.326704000	4.569607000
6	0.539738000	8.246994000	10.229965000	6	4.746974000	1.895822000	7.585155000
6	0.321761000	8.553102000	8.746871000	6	6.530819000	4.019089000	3.584451000
6	-0.248824000	9.949179000	8.507026000	6	6.563897000	1.672096000	5.146213000
6	-1.506692000	10.187446000	9.327742000	6	7.277848000	2.910667000	3.187681000
6	-2.657103000	9.317675000	8.823287000	6	7.291669000	1.757371000	3.962293000
6	3.301414000	4.313588000	8.882251000	1	3.334681000	5.777845000	7.344127000
1	-2.795185000	13.015772000	8.329418000	1	3.677152000	3.359791000	9.230216000
1	-1.063882000	12.257684000	9.582247000	1	6.519937000	4.921270000	2.982554000
1	-3.887626000	11.576238000	10.388167000	1	7.853919000	2.949333000	2.270749000
1	-2.749369000	12.645689000	11.195769000	1	7.881188000	0.905055000	3.644846000
1	-3.162195000	9.750448000	11.651319000	1	6.591750000	0.769483000	5.743117000
1	-2.113325000	10.815049000	12.573354000	1	3.678368000	1.848640000	7.796827000
1	-0.315750000	10.593607000	11.029739000	1	5.055027000	0.950977000	7.145651000
1	-1.504985000	7.824604000	10.775150000	1	5.294616000	2.048617000	8.517806000
1	-1.216074000	8.569607000	13.176438000	1	3.148302000	4.439521000	4.303212000
1	0.481865000	8.867452000	12.819953000	1	4.075375000	5.804327000	3.652179000
1	-1.052175000	6.240694000	12.672227000	1	3.073606000	6.026580000	5.095569000
1	0.277289000	6.594064000	13.752137000	1	6.082371000	6.703714000	4.914274000
1	1.289506000	8.969801000	10.592267000	1	6.572307000	5.967965000	6.451844000
1	-0.339446000	7.792390000	8.318500000	1	5.077116000	6.919000000	6.356628000
1	1.268349000	8.472202000	8.207631000				

Table S14. Optimized geometry of **TS**(**TTC-TTT**) of spiropyran (**3**). The bond lengths and Cartesian coordinates of the atoms are given in Angstroms, the torsion angles - in degrees.

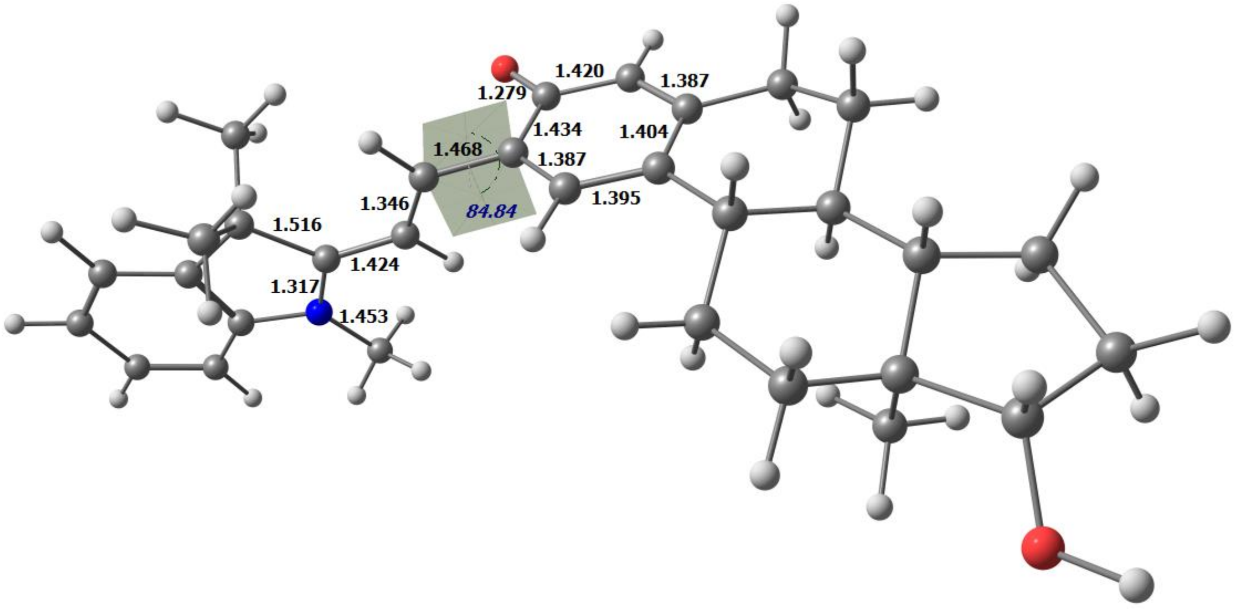

ν_{im} = -210.92 cm^{-1}

Atom	X	Y	Z	Atom	X	Y	Z
8	-4.851594000	3.335708000	15.446205000	1	-2.791639000	2.467857000	13.356672000
8	1.786620000	6.841247000	7.564051000	1	-3.951599000	2.959932000	12.125611000
6	-4.980776000	4.185519000	14.318942000	1	-2.929813000	5.650873000	15.314937000
6	-5.517447000	5.591704000	14.648358000	1	-2.388812000	3.973827000	15.326550000
6	-4.819216000	6.552090000	13.662731000	1	-1.653562000	5.132035000	14.218945000
6	-4.045681000	5.618358000	12.732911000	1	-0.008728000	8.360995000	8.717706000
6	-2.893761000	6.153692000	11.911019000	1	-0.392249000	3.579297000	9.766237000
6	-3.264494000	7.336873000	11.035911000	6	2.846687000	3.827273000	8.752596000
6	-2.018982000	7.920460000	10.395149000	6	3.811314000	2.899156000	8.267644000
6	-1.098807000	6.879533000	9.806209000	6	3.802432000	2.105263000	6.976517000
6	-0.088439000	7.301418000	8.954372000	7	4.914645000	2.647623000	8.941061000
6	0.868486000	6.437936000	8.358283000	6	5.124217000	1.392346000	7.066485000
6	0.707964000	5.059704000	8.718451000	6	5.752374000	1.745469000	8.250237000
6	-0.320170000	4.640755000	9.550350000	6	3.773638000	3.035265000	5.756542000
6	-1.237295000	5.519622000	10.126493000	6	2.654534000	1.086739000	6.938408000
6	-2.352561000	5.024144000	11.025275000	6	5.271280000	3.221470000	10.227050000
6	-1.983898000	3.788382000	11.848794000	6	5.735394000	0.507720000	6.201945000
6	-3.123562000	3.317495000	12.750628000	6	6.991365000	1.258224000	8.620799000
6	-3.639680000	4.444634000	13.633656000	6	6.983273000	-0.001187000	6.551029000
6	-2.593720000	4.829065000	14.679738000	6	7.599840000	0.369969000	7.741819000
6	1.657326000	4.080826000	8.175867000	1	3.099447000	4.372654000	9.654661000
1	-5.734816000	3.146219000	15.776576000	1	1.373042000	3.542423000	7.275442000
1	-5.640509000	3.705575000	13.582811000	1	5.259313000	0.216495000	5.272601000
1	-5.269128000	5.830470000	15.685376000	1	7.483087000	-0.695542000	5.886151000
1	-6.605395000	5.627105000	14.561751000	1	8.572294000	-0.036969000	7.991870000
1	-4.135162000	7.224318000	14.188635000	1	7.478447000	1.547126000	9.542812000
1	-5.527697000	7.179404000	13.119153000	1	4.408777000	3.206141000	10.889477000
1	-4.775331000	5.207467000	12.017067000	1	6.063250000	2.624961000	10.669321000
1	-2.091703000	6.480951000	12.587719000	1	5.619159000	4.246302000	10.091996000
1	-3.774719000	8.106631000	11.621876000	1	2.656057000	0.463523000	7.834189000
1	-3.971546000	7.000895000	10.266616000	1	2.793113000	0.444217000	6.067657000
1	-1.462874000	8.481143000	11.157687000	1	1.685871000	1.576782000	6.850877000
1	-2.283191000	8.649586000	9.623250000	1	3.888617000	2.431087000	4.855325000
1	-3.202001000	4.726454000	10.386870000	1	4.591160000	3.757064000	5.798710000
1	-1.091949000	4.007373000	12.445709000	1	2.830672000	3.577403000	5.694397000
1	-1.708349000	2.966776000	11.183531000				

Table S15. Optimized geometry of **TS**(**CTT-TTT**) of spiropyran (**3**). The bond lengths and Cartesian coordinates of the atoms are given in Angstroms, the torsion angles - in degrees.

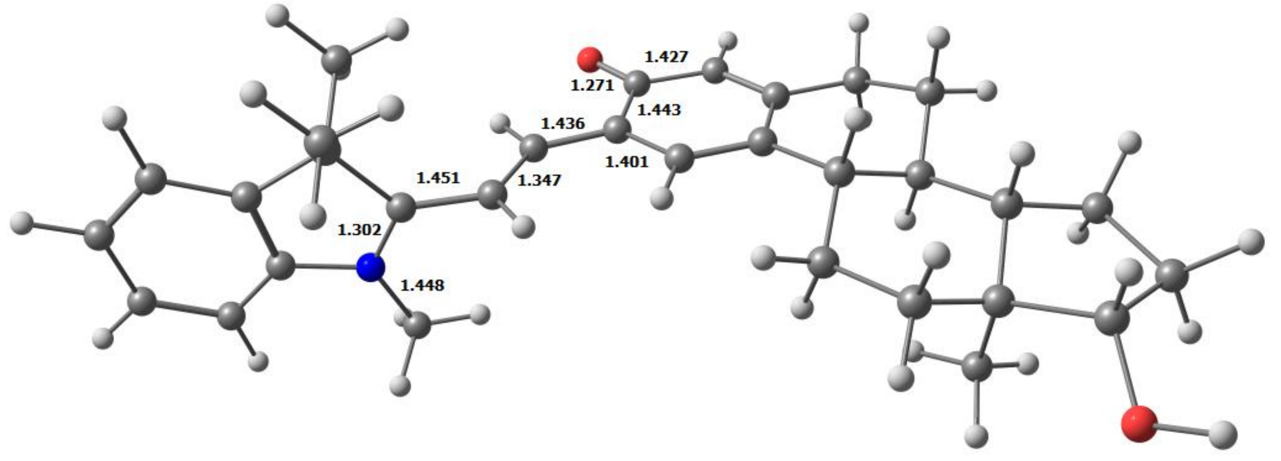

ν_{im} = -211.96 cm^{-1}

Atom	X	Y	Z	Atom	X	Y	Z
8	-4.851594000	3.335708000	15.446205000	1	-2.791639000	2.467857000	13.356672000
8	1.786620000	6.841247000	7.564051000	1	-3.951599000	2.959932000	12.125611000
6	-4.980776000	4.185519000	14.318942000	1	-2.929813000	5.650873000	15.314937000
6	-5.517447000	5.591704000	14.648358000	1	-2.388812000	3.973827000	15.326550000
6	-4.819216000	6.552090000	13.662731000	1	-1.653562000	5.132035000	14.218945000
6	-4.045681000	5.618358000	12.732911000	1	-0.008728000	8.360995000	8.717706000
6	-2.893761000	6.153692000	11.911019000	1	-0.392249000	3.579297000	9.766237000
6	-3.264494000	7.336873000	11.035911000	6	2.846687000	3.827273000	8.752596000
6	-2.018982000	7.920460000	10.395149000	6	3.811314000	2.899156000	8.267644000
6	-1.098807000	6.879533000	9.806209000	6	3.802432000	2.105263000	6.976517000
6	-0.088439000	7.301418000	8.954372000	7	4.914645000	2.647623000	8.941061000
6	0.868486000	6.437936000	8.358283000	6	5.124217000	1.392346000	7.066485000
6	0.707964000	5.059704000	8.718451000	6	5.752374000	1.745469000	8.250237000
6	-0.320170000	4.640755000	9.550350000	6	3.773638000	3.035265000	5.756542000
6	-1.237295000	5.519622000	10.126493000	6	2.654534000	1.086739000	6.938408000
6	-2.352561000	5.024144000	11.025275000	6	5.271280000	3.221470000	10.227050000
6	-1.983898000	3.788382000	11.848794000	6	5.735394000	0.507720000	6.201945000
6	-3.123562000	3.317495000	12.750628000	6	6.991365000	1.258224000	8.620799000
6	-3.639680000	4.444634000	13.633656000	6	6.983273000	-0.001187000	6.551029000
6	-2.593720000	4.829065000	14.679738000	6	7.599840000	0.369969000	7.741819000
6	1.657326000	4.080826000	8.175867000	1	3.099447000	4.372654000	9.654661000
1	-5.734816000	3.146219000	15.776576000	1	1.373042000	3.542423000	7.275442000
1	-5.640509000	3.705575000	13.582811000	1	5.259313000	0.216495000	5.272601000
1	-5.269128000	5.830470000	15.685376000	1	7.483087000	-0.695542000	5.886151000
1	-6.605395000	5.627105000	14.561751000	1	8.572294000	-0.036969000	7.991870000
1	-4.135162000	7.224318000	14.188635000	1	7.478447000	1.547126000	9.542812000
1	-5.527697000	7.179404000	13.119153000	1	4.408777000	3.206141000	10.889477000
1	-4.775331000	5.207467000	12.017067000	1	6.063250000	2.624961000	10.669321000
1	-2.091703000	6.480951000	12.587719000	1	5.619159000	4.246302000	10.091996000
1	-3.774719000	8.106631000	11.621876000	1	2.656057000	0.463523000	7.834189000
1	-3.971546000	7.000895000	10.266616000	1	2.793113000	0.444217000	6.067657000
1	-1.462874000	8.481143000	11.157687000	1	1.685871000	1.576782000	6.850877000
1	-2.283191000	8.649586000	9.623250000	1	3.888617000	2.431087000	4.855325000
1	-3.202001000	4.726454000	10.386870000	1	4.591160000	3.757064000	5.798710000
1	-1.091949000	4.007373000	12.445709000	1	2.830672000	3.577403000	5.694397000
1	-1.708349000	2.966776000	11.183531000				

Table S16. Optimized geometry of the open **TTT** form of spiropyran (**3**). The bond lengths and Cartesian coordinates of the atoms are given in Angstroms.

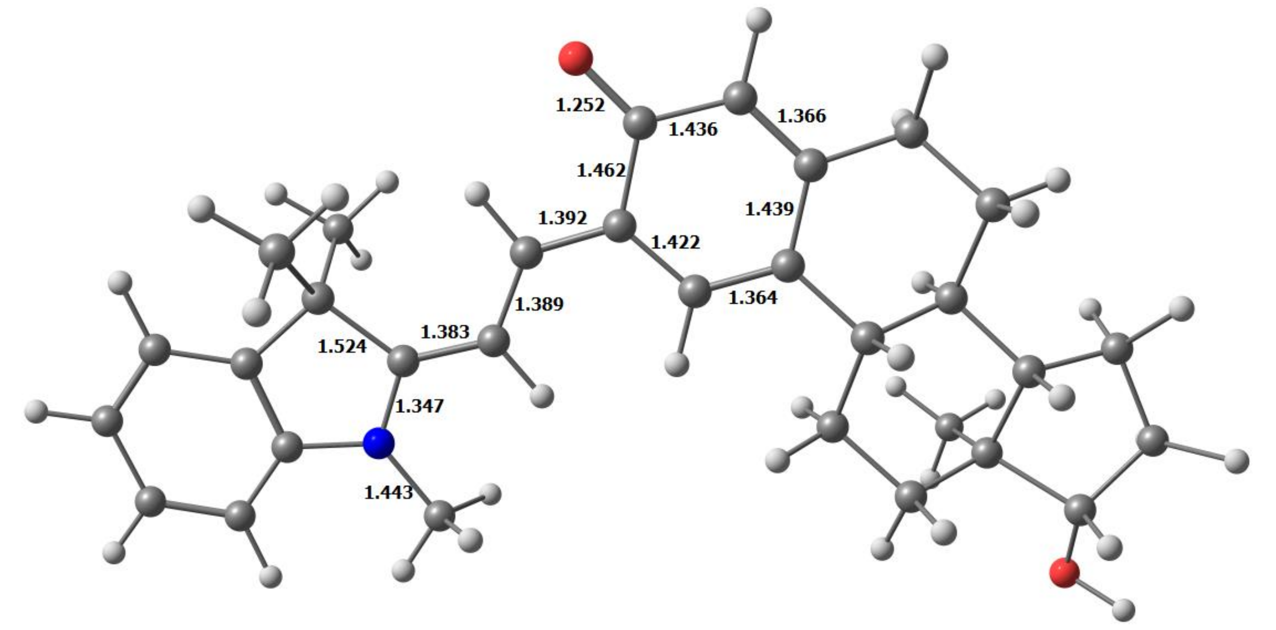

Atom	X	Y	Z	Atom	X	Y	Z
8	1.015630000	7.376652000	17.929332000	1	1.840010000	5.532826000	15.649329000
8	0.406109000	5.774426000	7.161764000	1	0.124680000	5.232816000	15.382307000
6	0.023458000	7.464402000	16.921725000	1	1.584743000	9.264409000	15.628032000
6	-0.718617000	8.812863000	16.876917000	1	2.625611000	7.895418000	16.014553000
6	-1.059587000	9.053983000	15.390699000	1	2.195626000	8.234436000	14.338050000
6	-0.603680000	7.769662000	14.698839000	1	-0.917250000	7.637492000	8.379731000
6	-0.348473000	7.769325000	13.206652000	1	1.558661000	4.588549000	11.372946000
6	-1.554462000	8.212482000	12.397968000	6	2.817445000	3.220490000	9.421911000
6	-1.194915000	8.322115000	10.928740000	6	3.601970000	2.268039000	8.796512000
6	-0.440742000	7.137890000	10.388423000	6	3.701980000	1.924600000	7.315107000
6	-0.378079000	6.959557000	9.035855000	7	4.428569000	1.461962000	9.490096000
6	0.385858000	5.920417000	8.405366000	6	4.720599000	0.812889000	7.338485000
6	1.147008000	5.071905000	9.321041000	6	5.115584000	0.578899000	8.650144000
6	1.022376000	5.269396000	10.723307000	6	4.223541000	3.100708000	6.482860000
6	0.248102000	6.243692000	11.281125000	6	2.366935000	1.402797000	6.771117000
6	0.042406000	6.348267000	12.777892000	6	4.583007000	1.504736000	10.924560000
6	1.200725000	5.786816000	13.602903000	6	5.256257000	0.067794000	6.310120000
6	0.958671000	5.890472000	15.107145000	6	6.045461000	-0.392798000	8.976803000
6	0.608780000	7.311921000	15.518600000	6	6.194391000	-0.917756000	6.614345000
6	1.818758000	8.232381000	15.360915000	6	6.579684000	-1.139740000	7.930733000
6	1.963637000	4.094117000	8.760244000	1	2.892824000	3.283713000	10.500659000
1	0.574122000	7.411073000	18.783253000	1	1.907438000	4.051879000	7.678832000
1	-0.704283000	6.653378000	17.064915000	1	4.954668000	0.242529000	5.282979000
1	-0.060174000	9.592274000	17.268156000	1	6.626169000	-1.514303000	5.819541000
1	-1.607849000	8.795058000	17.510241000	1	7.310735000	-1.908346000	8.153307000
1	-0.513484000	9.917156000	14.999808000	1	6.356787000	-0.576216000	9.997212000
1	-2.121332000	9.250133000	15.233557000	1	3.627269000	1.307071000	11.413864000
1	-1.385410000	7.014602000	14.878038000	1	5.297970000	0.747707000	11.232503000
1	0.482382000	8.452460000	12.981247000	1	4.948496000	2.484767000	11.237564000
1	-1.926320000	9.176792000	12.754207000	1	2.003043000	0.569531000	7.375004000
1	-2.364142000	7.486194000	12.542114000	1	2.510506000	1.051203000	5.747543000
1	-0.551959000	9.202087000	10.795926000	1	1.607140000	2.184212000	6.763681000
1	-2.079730000	8.502337000	10.312485000	1	4.407877000	2.759618000	5.462261000
1	-0.839802000	5.731298000	13.018379000	1	5.159998000	3.482224000	6.893928000
1	2.123528000	6.308710000	13.328136000	1	3.503112000	3.917401000	6.444600000
1	1.359476000	4.735021000	13.353900000				

Table S17. Optimized geometry of the N-protonated cationic form **SPH** of spiropyran (**3**). The bond lengths and Cartesian coordinates of the atoms are given in Angstroms.

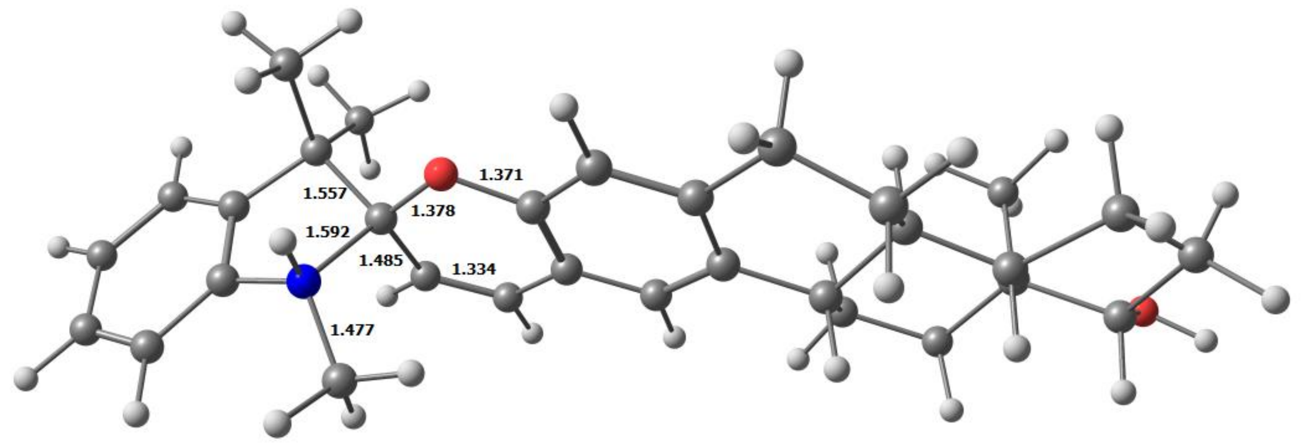

Atom	X	Y	Z	Atom	X	Y	Z
8	-5.150724000	9.004820000	11.502036000	1	-3.711646000	6.937122000	9.782857000
8	4.550650000	5.735720000	7.915848000	1	-3.301792000	8.255855000	8.688663000
6	-4.035733000	9.639361000	10.901889000	1	-2.467241000	8.627635000	12.885957000
6	-3.463141000	10.816250000	11.713488000	1	-3.445468000	7.295609000	12.272431000
6	-1.942007000	10.808276000	11.449770000	1	-1.706911000	7.318084000	11.987187000
6	-1.759030000	9.709466000	10.403542000	1	4.194047000	7.948280000	9.153952000
6	-0.401774000	9.068718000	10.211754000	1	0.034334000	5.740404000	7.710245000
6	0.710146000	10.060632000	9.927056000	6	3.494663000	3.960549000	6.653812000
6	2.049840000	9.349615000	9.923176000	6	4.727456000	4.702896000	7.021522000
6	2.042194000	8.061375000	9.147292000	6	5.900030000	3.780562000	7.467517000
6	3.268973000	7.474751000	8.842663000	7	5.337646000	5.339418000	5.695438000
6	3.317913000	6.290158000	8.143555000	6	6.436346000	3.334865000	6.133592000
6	2.153686000	5.654035000	7.727598000	6	6.123458000	4.242633000	5.138852000
6	0.933802000	6.245267000	8.042203000	6	5.411125000	2.645333000	8.353680000
6	0.847133000	7.439449000	8.748844000	6	6.977574000	4.578081000	8.209418000
6	-0.497764000	8.068725000	9.051868000	6	4.362452000	5.971613000	4.784193000
6	-1.618520000	7.048417000	9.267977000	6	7.178827000	2.213989000	5.807428000
6	-2.961955000	7.707568000	9.576074000	6	6.506779000	4.098622000	3.824452000
6	-2.853030000	8.686688000	10.735110000	6	7.586281000	2.041108000	4.489061000
6	-2.597252000	7.944004000	12.045108000	6	7.255686000	2.969744000	3.509366000
6	2.289896000	4.418691000	6.997041000	1	3.621336000	3.042038000	6.095757000
1	-5.887498000	9.622992000	11.488162000	1	1.394334000	3.868150000	6.729383000
1	-4.318804000	9.991078000	9.900017000	1	7.434863000	1.480960000	6.563511000
1	-3.687239000	10.656488000	12.770880000	1	8.165543000	1.165649000	4.219854000
1	-3.930627000	11.759316000	11.423335000	1	7.577715000	2.814800000	2.486861000
1	-1.388379000	10.569105000	12.362246000	1	6.241882000	4.822828000	3.064632000
1	-1.575192000	11.773627000	11.097571000	1	3.780302000	6.695704000	5.350210000
1	-2.039635000	10.142633000	9.430682000	1	4.910632000	6.483852000	3.996572000
1	-0.132562000	8.521502000	11.126096000	1	3.718453000	5.204570000	4.363841000
1	0.720549000	10.855949000	10.676375000	1	7.339516000	5.434002000	7.635528000
1	0.527146000	10.537649000	8.956420000	1	7.827112000	3.919768000	8.396540000
1	2.335479000	9.119560000	10.957417000	1	6.596703000	4.939384000	9.164979000
1	2.839339000	9.998083000	9.532821000	1	6.264863000	2.058171000	8.696361000
1	-0.780050000	8.665388000	8.169821000	1	4.725794000	1.979334000	7.829217000
1	-1.331122000	6.370078000	10.078173000	1	4.904448000	3.047509000	9.233466000
1	-1.737280000	6.428401000	8.376776000	1	5.971342000	6.073474000	6.026227000

Table S18. Optimized geometry of the protonated cationic form **CCCH** of spiropyran (**3**). The bond lengths and Cartesian coordinates of the atoms are given in Angstroms, the torsion angles - in degrees.

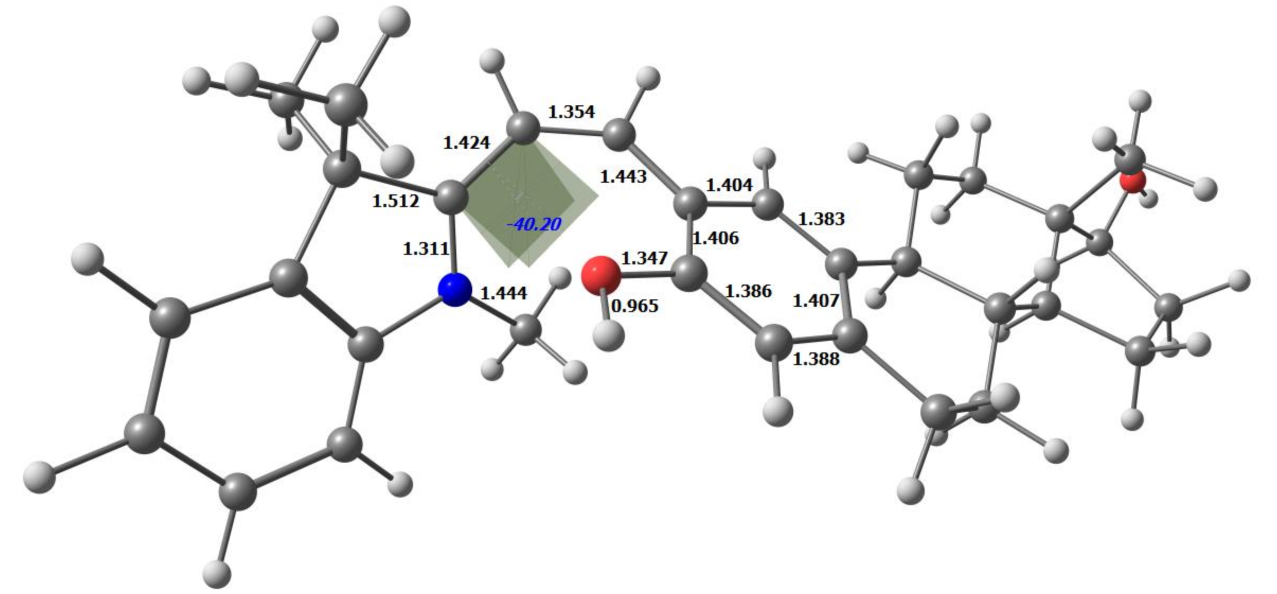

Atom	X	Y	Z	Atom	X	Y	Z
8	-5.010526000	9.447940000	11.371749000	1	-3.494369000	7.036745000	10.277459000
8	4.766808000	5.635998000	8.493387000	1	-3.136393000	8.028255000	8.866237000
6	-3.904740000	9.934156000	10.632577000	1	-2.312867000	9.529071000	12.806147000
6	-3.367814000	11.294647000	11.114216000	1	-3.253789000	8.056755000	12.573587000
6	-1.846836000	11.261494000	10.851951000	1	-1.514285000	8.049360000	12.285123000
6	-1.632804000	9.935917000	10.122031000	1	4.351870000	8.118991000	9.207627000
6	-0.256220000	9.308939000	10.081901000	1	0.282522000	5.463218000	8.557142000
6	0.816171000	10.228155000	9.526371000	6	3.242354000	3.252965000	7.422111000
6	2.180722000	9.583596000	9.674481000	6	4.427304000	3.561868000	6.695800000
6	2.211695000	8.146700000	9.238564000	6	5.652085000	2.674906000	6.700088000
6	3.445389000	7.541150000	9.046770000	7	4.605577000	4.577374000	5.885473000
6	3.562483000	6.218039000	8.651783000	6	6.578732000	3.452424000	5.809394000
6	2.401646000	5.451993000	8.442918000	6	5.911289000	4.575112000	5.344076000
6	1.167662000	6.073994000	8.690434000	6	5.312861000	1.306481000	6.090951000
6	1.033140000	7.400097000	9.057537000	6	6.196245000	2.508874000	8.121043000
6	-0.329947000	8.038801000	9.224120000	6	3.624034000	5.564574000	5.501393000
6	-1.408654000	7.075506000	9.722988000	6	7.880559000	3.220250000	5.415355000
6	-2.773213000	7.748392000	9.862909000	6	6.483042000	5.501463000	4.494953000
6	-2.695455000	9.003653000	10.718730000	6	8.484914000	4.138289000	4.559725000
6	-2.422201000	8.643048000	12.178260000	6	7.797008000	5.260484000	4.108685000
6	2.369190000	4.056874000	8.074677000	1	3.037262000	2.187497000	7.471248000
1	-5.754137000	10.037440000	11.214671000	1	1.478047000	3.546117000	8.431952000
1	-4.187216000	10.007888000	9.573093000	1	8.422007000	2.348292000	5.764361000
1	-3.587505000	11.402610000	12.179079000	1	9.508373000	3.978370000	4.241631000
1	-3.861847000	12.119610000	10.597188000	1	8.291084000	5.961502000	3.446740000
1	-1.287978000	11.279464000	11.791707000	1	5.947746000	6.376352000	4.148828000
1	-1.506657000	12.114136000	10.262217000	1	2.629701000	5.180973000	5.712186000
1	-1.931390000	10.096010000	9.074192000	1	3.785532000	6.491381000	6.054097000
1	0.039720000	9.026773000	11.101925000	1	3.729966000	5.756721000	4.434940000
1	0.808497000	11.189869000	10.045189000	1	6.425568000	3.475561000	8.569519000
1	0.602042000	10.431737000	8.470219000	1	7.107897000	1.910405000	8.084020000
1	2.480332000	9.619646000	10.729661000	1	5.465694000	1.993425000	8.746836000
1	2.946039000	10.141415000	9.127532000	1	6.230129000	0.719453000	6.023928000
1	-0.646048000	8.376754000	8.224397000	1	4.890644000	1.411831000	5.090234000
1	-1.091924000	6.648323000	10.680300000	1	4.605503000	0.768280000	6.723539000
1	-1.511635000	6.235741000	9.032141000	1	5.467192000	6.254680000	8.733871000

Table S19. Optimized geometry of the protonated cationic form **TCCH** of spiropyran (**3**). The bond lengths and Cartesian coordinates of the atoms are given in Angstroms, the torsion angles - in degrees.

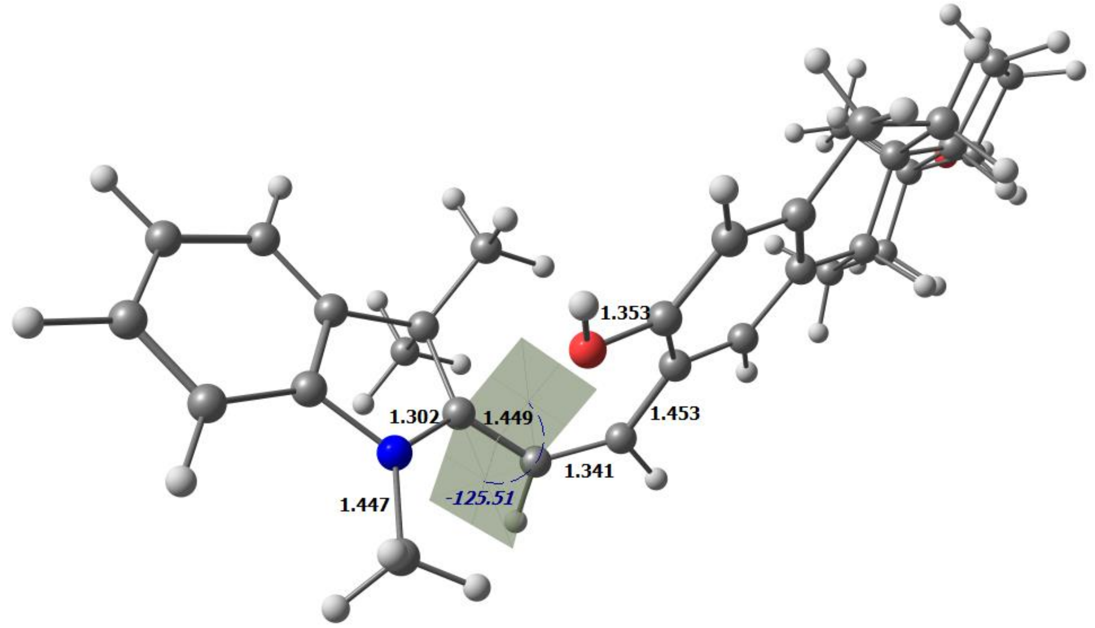

Atom	X	Y	Z	Atom	X	Y	Z
8	-4.161518000	8.262523000	12.762822000	1	-3.117827000	6.542292000	10.472510000
8	4.098109000	6.756782000	5.867929000	1	-3.275356000	7.962485000	9.440896000
6	-3.408395000	9.097041000	11.901090000	1	-1.152692000	8.253929000	13.163341000
6	-2.760272000	10.308000000	12.598121000	1	-2.082529000	6.811723000	12.761162000
6	-1.431247000	10.558834000	11.854400000	1	-0.555434000	7.135161000	11.942106000
6	-1.465041000	9.558472000	10.699940000	1	3.861981000	8.913133000	7.349396000
6	-0.176773000	9.161713000	10.011843000	1	-0.087691000	6.008789000	7.285314000
6	0.603812000	10.341415000	9.460871000	6	2.752068000	4.140899000	5.452757000
6	1.949774000	9.882322000	8.931060000	6	4.151444000	4.063769000	5.822482000
6	1.877776000	8.626098000	8.108862000	6	4.691245000	3.692711000	7.186833000
6	2.989770000	8.264205000	7.355938000	7	5.112930000	4.058094000	4.944236000
6	3.021863000	7.096533000	6.614365000	6	6.164794000	3.617798000	6.905074000
6	1.906916000	6.247684000	6.608904000	6	6.369040000	3.818097000	5.548830000
6	0.781379000	6.654709000	7.331494000	6	4.133710000	2.286655000	7.499609000
6	0.736821000	7.809466000	8.098109000	6	4.330853000	4.646977000	8.320888000
6	-0.511261000	8.202403000	8.861676000	6	4.975114000	4.306940000	3.525425000
6	-1.348874000	7.009119000	9.325605000	6	7.247119000	3.374707000	7.726400000
6	-2.589366000	7.430590000	10.111836000	6	7.611260000	3.783357000	4.949059000
6	-2.232512000	8.356052000	11.265274000	6	8.515052000	3.336168000	7.151352000
6	-1.455657000	7.601306000	12.342631000	6	8.693895000	3.535480000	5.785689000
6	1.813405000	5.010965000	5.852665000	1	2.439299000	3.337216000	4.788978000
1	-4.922551000	8.763068000	13.071687000	1	0.802161000	4.768667000	5.535498000
1	-4.055754000	9.448757000	11.085606000	1	7.118123000	3.216142000	8.790893000
1	-2.590258000	10.060883000	13.648749000	1	9.378708000	3.147089000	7.777966000
1	-3.421361000	11.176584000	12.573801000	1	9.691518000	3.499256000	5.364968000
1	-0.574717000	10.363729000	12.505872000	1	7.744508000	3.940233000	3.886144000
1	-1.338001000	11.587843000	11.503766000	1	3.937324000	4.534109000	3.300509000
1	-2.115202000	9.986340000	9.921037000	1	5.605456000	5.155186000	3.256768000
1	0.467750000	8.637755000	10.731452000	1	5.300021000	3.424446000	2.973609000
1	0.755670000	11.100346000	10.232462000	1	4.734199000	5.642818000	8.147834000
1	0.019242000	10.811575000	8.660802000	1	4.757587000	4.251228000	9.244047000
1	2.619294000	9.687027000	9.778295000	1	3.249926000	4.710730000	8.443646000
1	2.433053000	10.669803000	8.345635000	1	4.587502000	1.936232000	8.427391000
1	-1.145169000	8.782422000	8.171969000	1	4.369743000	1.578700000	6.703247000
1	-0.722177000	6.343889000	9.929307000	1	3.051518000	2.335920000	7.627822000
1	-1.672940000	6.422883000	8.462695000	1	4.783752000	7.430162000	5.951995000

Table S20. Optimized geometry of **TS**[5] (**CCCH**-to-**CTCH**) of the protonated spiropyran (**3**). The bond lengths and Cartesian coordinates of the atoms are given in Angstroms, the torsion angles - in degrees.

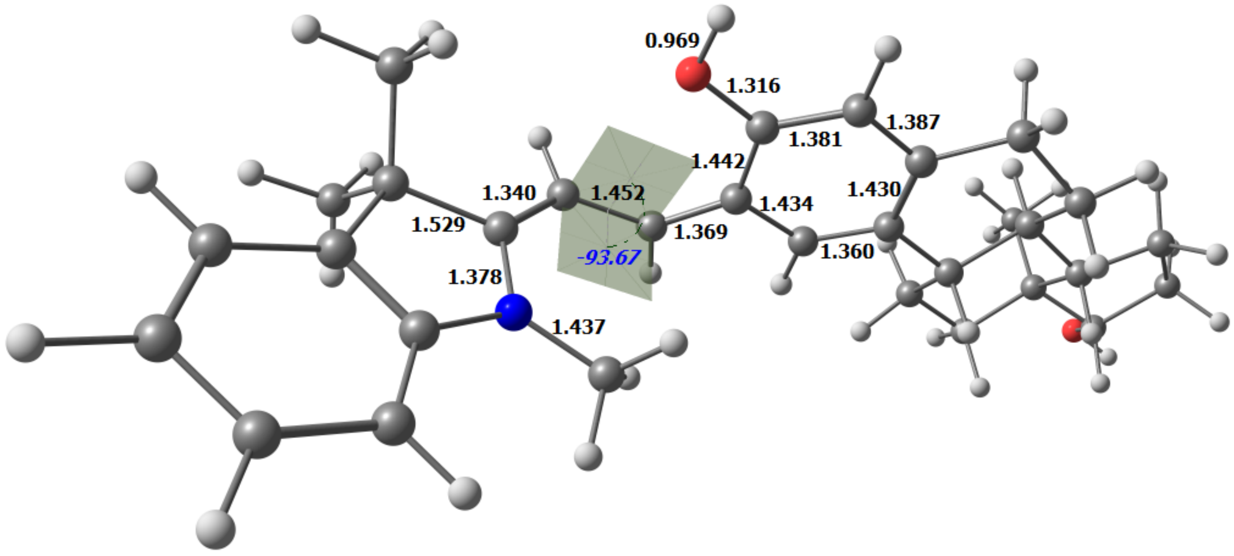

ν_{im} = -747.97 cm^{-1}

Atom	X	Y	Z	Atom	X	Y	Z
8	-5.276032000	9.308197000	10.767770000	1	-3.542034000	7.109984000	9.565736000
8	4.949217000	5.979832000	9.309047000	1	-2.964117000	8.317058000	8.419773000
6	-4.077436000	9.907012000	10.310294000	1	-2.859438000	9.157570000	12.631031000
6	-3.664991000	11.173728000	11.082322000	1	-3.712942000	7.744108000	12.013574000
6	-2.121319000	11.185544000	11.073486000	1	-1.949779000	7.777735000	12.025059000
6	-1.752940000	9.986959000	10.199825000	1	4.350965000	8.393272000	10.126284000
6	-0.373341000	9.376189000	10.309764000	1	0.528468000	5.755731000	8.448396000
6	0.744388000	10.372829000	10.066066000	6	3.940931000	3.730621000	7.955071000
6	2.090075000	9.727797000	10.327449000	6	4.750312000	3.673837000	6.888170000
6	2.243464000	8.356642000	9.761818000	6	5.972451000	2.758966000	6.804725000
6	3.517447000	7.807958000	9.750274000	7	4.665955000	4.402039000	5.721344000
6	3.753650000	6.528665000	9.287695000	6	6.575754000	3.185856000	5.488889000
6	2.645374000	5.762937000	8.773955000	6	5.763122000	4.157987000	4.906342000
6	1.342493000	6.359827000	8.829465000	6	5.540924000	1.291835000	6.762492000
6	1.112795000	7.618521000	9.291222000	6	6.931818000	2.997608000	7.967904000
6	-0.263710000	8.244160000	9.278723000	6	3.644413000	5.365086000	5.415203000
6	-1.395046000	7.222560000	9.403954000	6	7.722820000	2.766138000	4.853675000
6	-2.776112000	7.875247000	9.406038000	6	6.086980000	4.738200000	3.689757000
6	-2.875628000	8.974044000	10.453241000	6	8.063586000	3.332090000	3.622482000
6	-2.841366000	8.383408000	11.861925000	6	7.251977000	4.306010000	3.057502000
6	2.753866000	4.539104000	8.169773000	1	4.180396000	3.077927000	8.793953000
1	-5.999623000	9.914433000	10.582948000	1	1.798657000	4.114907000	7.852289000
1	-4.180962000	10.148179000	9.243106000	1	8.355678000	2.008358000	5.304125000
1	-4.056873000	11.109041000	12.100114000	1	8.965002000	3.014904000	3.111637000
1	-4.091272000	12.069957000	10.627595000	1	7.526551000	4.746905000	2.105619000
1	-1.724553000	11.062653000	12.085131000	1	5.469644000	5.506050000	3.240118000
1	-1.713772000	12.118326000	10.680762000	1	2.674023000	5.005124000	5.758190000
1	-1.867874000	10.304973000	9.151792000	1	3.846674000	6.339685000	5.871131000
1	-0.242154000	8.956060000	11.316412000	1	3.577085000	5.490218000	4.335145000
1	0.634314000	11.245485000	10.712963000	1	7.233767000	4.045358000	8.017630000
1	0.688174000	10.730076000	9.031445000	1	7.826432000	2.383925000	7.840655000
1	2.239159000	9.623406000	11.411669000	1	6.461941000	2.725097000	8.915570000
1	2.921767000	10.348567000	9.981836000	1	6.415463000	0.651437000	6.626135000
1	-0.376054000	8.731435000	8.297778000	1	4.847300000	1.112374000	5.938569000
1	-1.246918000	6.637166000	10.317229000	1	5.050238000	1.013911000	7.698260000
1	-1.352876000	6.513760000	8.573676000	1	5.601483000	6.592215000	9.681165000

Table S21. Optimized geometry of the protonated cationic form **CTCH** of spiropyran (**3**). The bond lengths and Cartesian coordinates of the atoms are given in Angstroms, the torsion angles - in degrees.

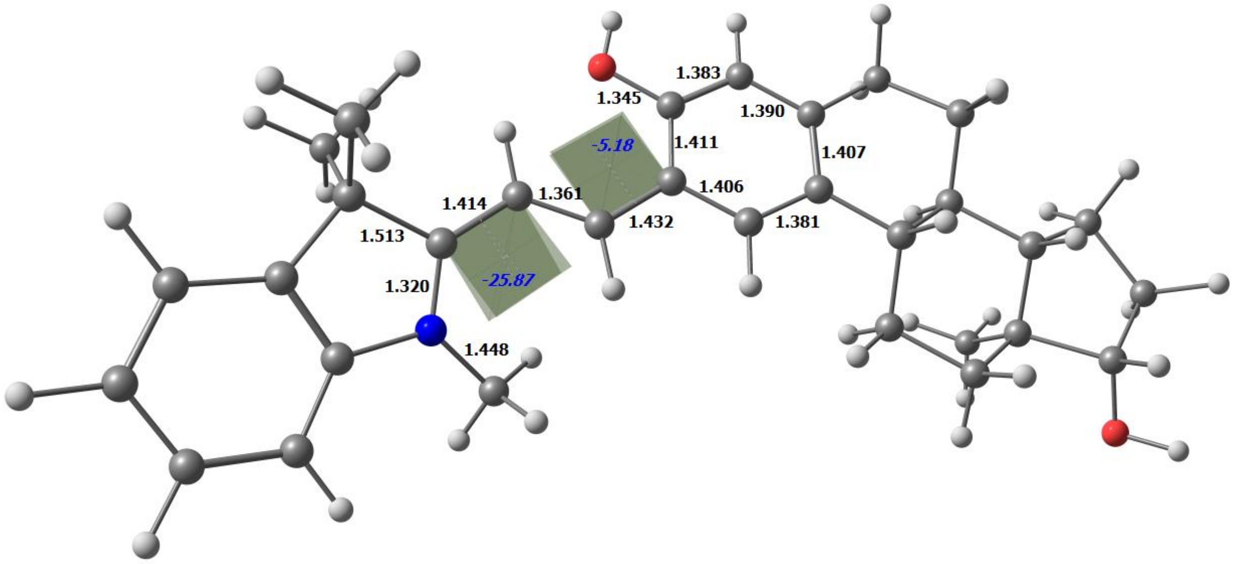

Atom	X	Y	Z	Atom	X	Y	Z
8	-4.955595000	10.087812000	9.066196000	1	-2.735882000	8.460140000	7.740916000
8	4.834788000	5.576599000	10.543334000	1	-1.713802000	9.886498000	7.902979000
6	-3.719690000	10.504109000	9.619254000	1	-3.828139000	8.387042000	11.326318000
6	-3.820026000	11.043087000	11.058742000	1	-4.174489000	7.769198000	9.711741000
6	-2.499417000	10.643594000	11.750609000	1	-2.672456000	7.329152000	10.523339000
6	-1.673478000	10.021597000	10.625578000	1	3.689050000	7.129452000	12.310398000
6	-0.518473000	9.102846000	10.958979000	1	1.468572000	7.018853000	7.938357000
6	0.504184000	9.719801000	11.894631000	6	4.661027000	4.761312000	7.812872000
6	1.530507000	8.679673000	12.301277000	6	5.065411000	4.092595000	6.634009000
6	2.048526000	7.868707000	11.149594000	6	6.520103000	3.772644000	6.365517000
6	3.193212000	7.104831000	11.343665000	7	4.316981000	3.652848000	5.639192000
6	3.727600000	6.313616000	10.342433000	6	6.441574000	3.141091000	5.006540000
6	3.097691000	6.275023000	9.080396000	6	5.112602000	3.092498000	4.613658000
6	1.939380000	7.054995000	8.913738000	6	7.042997000	2.781699000	7.414417000
6	1.394991000	7.848822000	9.903853000	6	7.360104000	5.053384000	6.362228000
6	0.169726000	8.700908000	9.648452000	6	2.871188000	3.637785000	5.569228000
6	-0.814533000	8.083361000	8.652395000	6	7.422601000	2.645497000	4.173244000
6	-2.036047000	8.969254000	8.411193000	6	4.704375000	2.571416000	3.400990000
6	-2.714960000	9.356494000	9.716367000	6	7.039754000	2.111914000	2.944957000
6	-3.380442000	8.142042000	10.361476000	6	5.701765000	2.077668000	2.566754000
6	3.534336000	5.511778000	7.950675000	1	5.384865000	4.737224000	8.614637000
1	-5.506009000	10.867718000	8.947938000	1	2.880892000	5.617535000	7.091957000
1	-3.278771000	11.274550000	8.971504000	1	8.466798000	2.672417000	4.463686000
1	-4.682955000	10.584432000	11.547505000	1	7.794302000	1.720875000	2.272748000
1	-3.982627000	12.122730000	11.064407000	1	5.427150000	1.662590000	1.604384000
1	-2.679130000	9.913804000	12.545052000	1	3.665245000	2.550468000	3.098626000
1	-1.993577000	11.495362000	12.208056000	1	2.499323000	4.540371000	5.081777000
1	-1.252158000	10.853265000	10.039709000	1	2.563171000	2.766599000	4.995294000
1	-0.910873000	8.197665000	11.442802000	1	2.463324000	3.566780000	6.575616000
1	0.019392000	10.121461000	12.787710000	1	6.983595000	5.767954000	5.628372000
1	0.993222000	10.561546000	11.389365000	1	8.391038000	4.801471000	6.108351000
1	1.071299000	7.984714000	13.016121000	1	7.347935000	5.519906000	7.348592000
1	2.373961000	9.134727000	12.828021000	1	8.064457000	2.501925000	7.151669000
1	0.523300000	9.643701000	9.201657000	1	6.429267000	1.879930000	7.444215000
1	-1.121508000	7.097790000	9.017932000	1	7.054588000	3.237988000	8.405403000
1	-0.321304000	7.913262000	7.692782000	1	5.154001000	5.702406000	11.445784000

Table S22. Optimized geometry of **TS**^{6} (**CTCH**-to-**TTCH**) of the protonated spiropyran (**3**). The bond lengths and Cartesian coordinates of the atoms are given in Angstroms, the torsion angles - in degrees.

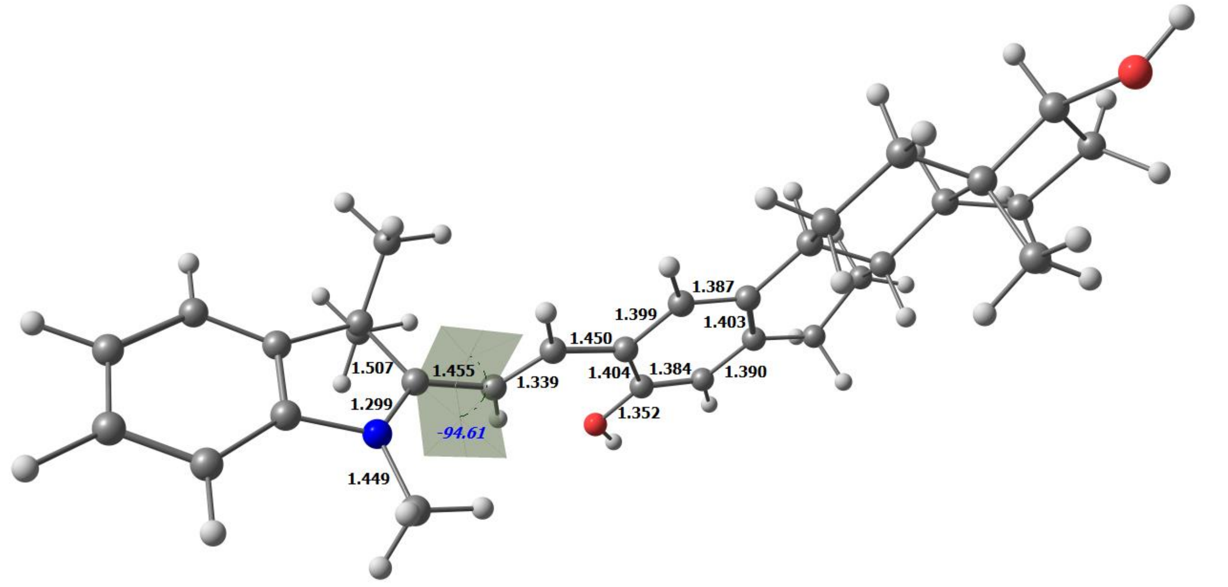

ν_{im} = -125.23 cm^{-1}

Atom	X	Y	Z	Atom	X	Y	Z
8	-4.315060000	11.052142000	8.667123000	1	-2.173366000	9.254827000	7.438401000
8	4.253048000	4.662132000	10.785501000	1	-0.994060000	10.422190000	8.029627000
6	-3.166825000	11.120541000	9.493551000	1	-3.953730000	8.815594000	10.695808000
6	-3.470043000	11.412708000	10.974780000	1	-4.048758000	8.539356000	8.957203000
6	-2.385358000	10.666395000	11.780706000	1	-2.833727000	7.700622000	9.920368000
6	-1.461232000	10.096287000	10.705569000	1	2.998281000	6.043646000	12.621811000
6	-0.544262000	8.937779000	11.032046000	1	1.693678000	7.099632000	8.016873000
6	0.357879000	9.191783000	12.226075000	6	4.485820000	4.355553000	7.977688000
6	1.116243000	7.927108000	12.580339000	6	4.991702000	3.868269000	6.703832000
6	1.728521000	7.249537000	11.386665000	6	6.131592000	4.484646000	5.935255000
6	2.699396000	6.278162000	11.603027000	7	4.560885000	2.799901000	6.104442000
6	3.308335000	5.602737000	10.559688000	6	6.252437000	3.534675000	4.778637000
6	2.946311000	5.898908000	9.235496000	6	5.294457000	2.542343000	4.916633000
6	1.962576000	6.874987000	9.042751000	6	7.389725000	4.509322000	6.812916000
6	1.338760000	7.559418000	10.075437000	6	5.759232000	5.904167000	5.490531000
6	0.316798000	8.643244000	9.797099000	6	3.488007000	1.933870000	6.550802000
6	-0.542929000	8.385610000	8.557320000	6	7.106242000	3.508023000	3.694505000
6	-1.543727000	9.510970000	8.296673000	6	5.132308000	1.503414000	4.023970000
6	-2.394835000	9.801715000	9.523900000	6	6.969379000	2.470899000	2.775563000
6	-3.359056000	8.647694000	9.796242000	6	5.999897000	1.485540000	2.937602000
6	3.521430000	5.281776000	8.055799000	1	4.972246000	3.949894000	8.857751000
1	-4.719813000	11.924538000	8.649858000	1	3.093522000	5.639782000	7.121968000
1	-2.488299000	11.890416000	9.100121000	1	7.865381000	4.269885000	3.559981000
1	-4.469463000	11.037807000	11.208318000	1	7.630303000	2.428724000	1.917888000
1	-3.472820000	12.486265000	11.173265000	1	5.917446000	0.690002000	2.206993000
1	-2.825997000	9.862727000	12.377310000	1	4.376479000	0.739445000	4.154897000
1	-1.854650000	11.322992000	12.471851000	1	2.721075000	1.897946000	5.777166000
1	-0.810834000	10.920339000	10.372702000	1	3.888700000	0.932993000	6.712187000
1	-1.151173000	8.049696000	11.256924000	1	3.072823000	2.330412000	7.472741000
1	-0.225786000	9.523537000	13.088335000	1	4.846601000	5.907531000	4.892337000
1	1.056501000	10.000971000	11.980798000	1	6.576238000	6.301263000	4.886459000
1	0.426709000	7.218623000	13.056793000	1	5.620316000	6.547784000	6.359919000
1	1.896891000	8.127815000	13.319574000	1	8.215488000	4.913326000	6.225704000
1	0.879863000	9.570001000	9.602286000	1	7.656085000	3.506829000	7.151668000
1	-1.065015000	7.430264000	8.677117000	1	7.229263000	5.150491000	7.681073000
1	0.091718000	8.279901000	7.674752000	1	4.392058000	4.564484000	11.735172000

Table S23. Optimized geometry of the protonated cationic form **TTCH** of spiropyran (**3**). The bond lengths and Cartesian coordinates of the atoms are given in Angstroms, the torsion angles - in degrees.

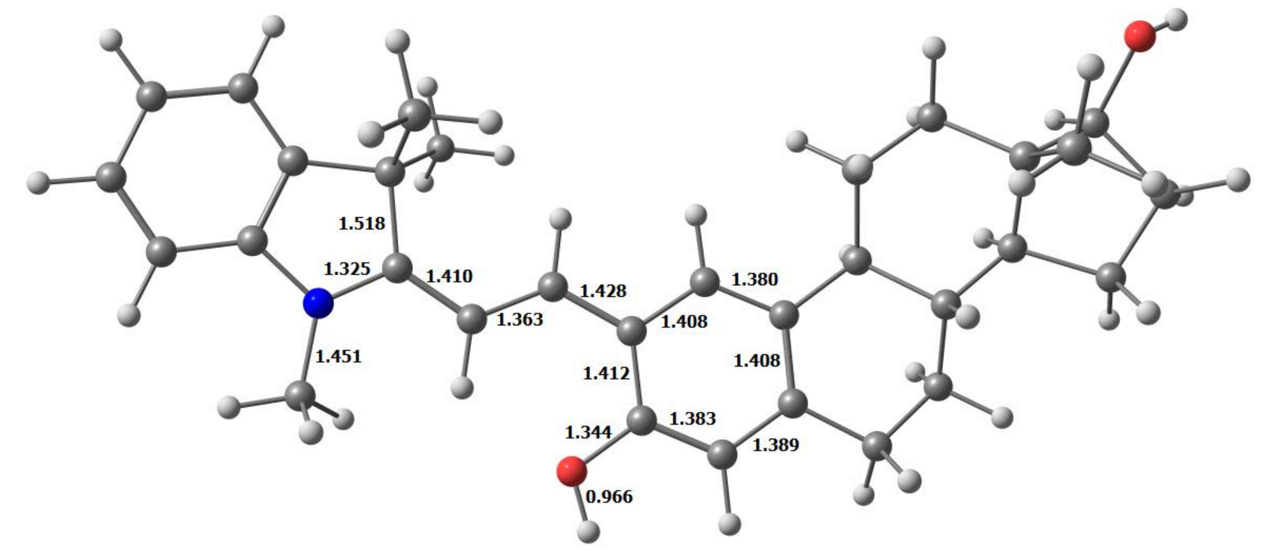

Atom	X	Y	Z	Atom	X	Y	Z
8	-3.174381000	12.257038000	8.667194000	1	-1.038982000	10.426134000	7.478791000
8	3.304954000	3.720236000	10.625998000	1	0.059782000	11.075855000	8.693329000
6	-2.332014000	11.831997000	9.723085000	1	-3.770275000	9.532966000	9.875195000
6	-3.022031000	11.773173000	11.098270000	1	-3.370346000	9.766265000	8.173954000
6	-2.350207000	10.607430000	11.854694000	1	-2.639035000	8.442585000	9.080678000
6	-1.239126000	10.152631000	10.909799000	1	1.836348000	4.854996000	12.465807000
6	-0.641480000	8.770731000	11.058237000	1	2.009004000	7.278806000	8.203211000
6	-0.098178000	8.497288000	12.449045000	6	4.179596000	4.026554000	7.933797000
6	0.351935000	7.053951000	12.563773000	6	4.844709000	3.711258000	6.731150000
6	1.166135000	6.583578000	11.394498000	6	4.855902000	4.456713000	5.408386000
6	1.872379000	5.394171000	11.522903000	7	5.592728000	2.621533000	6.634669000
6	2.623195000	4.869997000	10.486361000	6	5.745838000	3.566908000	4.581834000
6	2.676161000	5.555716000	9.253045000	6	6.160194000	2.490863000	5.352659000
6	1.953111000	6.759869000	9.152864000	6	5.486338000	5.849067000	5.546933000
6	1.205650000	7.297426000	10.181179000	6	3.454733000	4.527041000	4.786091000
6	0.493253000	8.625729000	10.035629000	6	5.810226000	1.669363000	7.707133000
6	0.025279000	8.927112000	8.610664000	6	6.165719000	3.680131000	3.273049000
6	-0.672587000	10.282093000	8.500216000	6	6.994314000	1.500794000	4.867390000
6	-1.804574000	10.413299000	9.507684000	6	7.005346000	2.695416000	2.758905000
6	-2.960722000	9.482174000	9.145037000	6	7.411514000	1.623911000	3.547015000
6	3.401979000	5.133889000	8.097316000	1	4.303451000	3.341947000	8.758245000
1	-3.407394000	13.176808000	8.825110000	1	3.295917000	5.810447000	7.257761000
1	-1.462266000	12.501301000	9.779729000	1	5.852048000	4.515426000	2.657013000
1	-4.088928000	11.590261000	10.949286000	1	7.347632000	2.764374000	1.733169000
1	-2.928307000	12.723144000	11.628226000	1	8.065826000	0.868319000	3.128847000
1	-3.062027000	9.796635000	12.034210000	1	7.318748000	0.663375000	5.470946000
1	-1.960512000	10.911120000	12.827558000	1	6.465172000	0.879761000	7.354088000
1	-0.406020000	10.862508000	11.030168000	1	6.276816000	2.168388000	8.556926000
1	-1.412110000	8.018023000	10.841361000	1	4.859227000	1.234241000	8.015187000
1	-0.856141000	8.704636000	13.208176000	1	3.009888000	3.532927000	4.718235000
1	0.742278000	9.174721000	12.642171000	1	3.540755000	4.937699000	3.778886000
1	-0.531596000	6.405850000	12.630802000	1	2.788663000	5.169430000	5.361368000
1	0.915365000	6.883686000	13.485522000	1	5.590681000	6.284818000	4.552028000
1	1.223652000	9.407075000	10.299433000	1	6.475947000	5.783364000	6.002051000
1	-0.640386000	8.125171000	8.274266000	1	4.868186000	6.514314000	6.148764000
1	0.877969000	8.924577000	7.927888000	1	3.177717000	3.369145000	11.516451000

Table S24. Optimized geometry of the protonated cationic form **CTTH** of spiropyran (**3**). The bond lengths and Cartesian coordinates of the atoms are given in Angstroms, the torsion angles - in degrees.

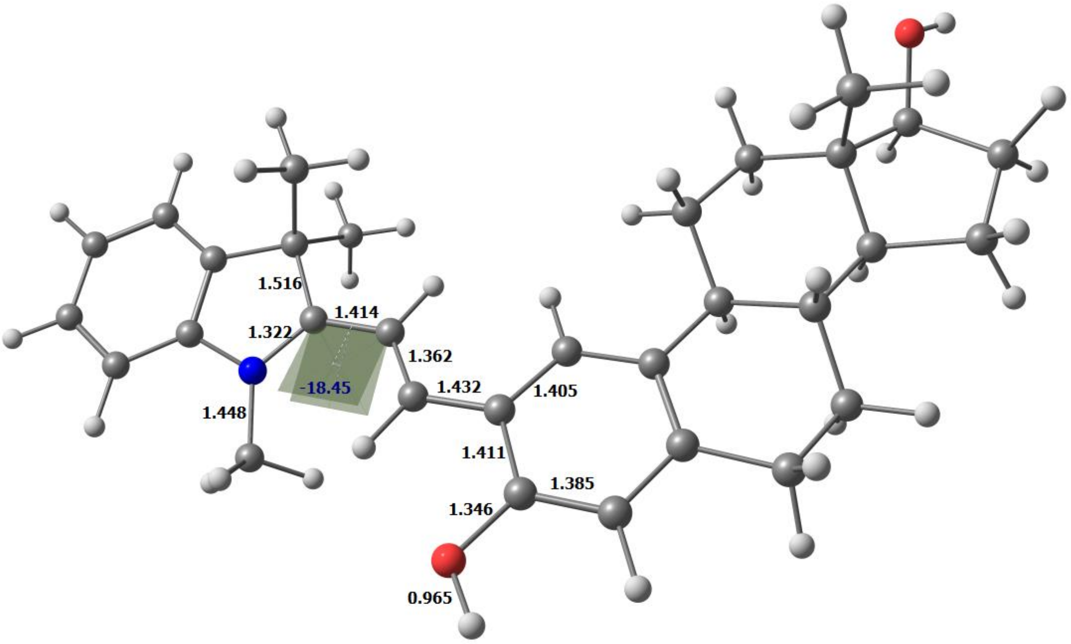

Atom	X	Y	Z	Atom	X	Y	Z
8	-0.921914000	2.339911000	16.149981000	1	0.338439000	2.213196000	13.361199000
8	4.131562000	8.085830000	8.412454000	1	-0.962116000	3.276384000	12.832259000
6	-1.125398000	3.593627000	15.522035000	1	1.387267000	4.308877000	16.281357000
6	-1.235079000	4.781317000	16.497936000	1	1.517291000	2.717637000	15.535692000
6	-0.614540000	5.989445000	15.762847000	1	2.208108000	4.126794000	14.733833000
6	-0.318446000	5.449564000	14.362532000	1	3.056368000	9.195091000	10.542732000
6	0.679448000	6.162329000	13.472120000	1	1.910802000	4.459762000	9.878550000
6	0.358853000	7.631167000	13.252400000	6	3.295466000	4.167878000	7.568933000
6	1.484789000	8.304638000	12.489592000	6	3.843699000	3.487279000	6.457681000
6	1.978595000	7.512316000	11.313242000	6	3.828232000	1.973885000	6.370596000
6	2.806873000	8.147320000	10.398131000	7	4.409648000	3.993938000	5.375133000
6	3.325555000	7.478436000	9.302338000	6	4.560406000	1.742577000	5.080667000
6	2.999070000	6.121976000	9.092901000	6	4.889443000	2.970460000	4.527020000
6	2.160192000	5.501446000	10.034177000	6	2.378182000	1.471624000	6.286489000
6	1.645568000	6.151201000	11.138852000	6	4.549559000	1.344797000	7.566993000
6	0.719393000	5.446401000	12.112980000	6	4.499644000	5.390636000	5.005335000
6	1.001867000	3.948050000	12.261995000	6	4.914213000	0.578444000	4.429478000
6	0.053339000	3.264450000	13.248049000	6	5.582450000	3.096810000	3.337406000
6	0.033139000	3.972480000	14.596312000	6	5.608587000	0.676218000	3.226142000
6	1.362578000	3.778241000	15.327682000	6	5.938902000	1.917394000	2.692451000
6	3.527520000	5.457381000	7.939278000	1	2.685029000	3.537680000	8.205505000
1	-1.719587000	2.120480000	16.640988000	1	4.198791000	6.063050000	7.346405000
1	-2.035216000	3.541935000	14.907723000	1	4.661487000	-0.391382000	4.843507000
1	-0.683996000	4.540811000	17.410575000	1	5.899810000	-0.225738000	2.700621000
1	-2.273211000	4.959170000	16.786628000	1	6.486248000	1.971467000	1.758711000
1	0.306367000	6.315157000	16.255034000	1	5.851200000	4.058640000	2.919272000
1	-1.285190000	6.850171000	15.738294000	1	5.388747000	5.850540000	5.440942000
1	-1.274569000	5.444978000	13.815283000	1	4.555196000	5.459402000	3.921684000
1	1.676424000	6.102105000	13.931426000	1	3.607739000	5.912782000	5.347647000
1	0.210067000	8.143385000	14.206286000	1	5.574124000	1.712192000	7.648243000
1	-0.582538000	7.713649000	12.695659000	1	4.577401000	0.261585000	7.435852000
1	2.336130000	8.455612000	13.165921000	1	4.020712000	1.565075000	8.495915000
1	1.194817000	9.303807000	12.151302000	1	2.389846000	0.390841000	6.134655000
1	-0.299701000	5.535096000	11.703131000	1	1.847072000	1.935384000	5.453327000
1	2.042393000	3.807253000	12.574186000	1	1.840723000	1.682763000	7.212377000
1	0.904006000	3.449903000	11.294318000	1	4.282768000	9.001847000	8.675462000

Table S25. Optimized geometry of **TS**^{7} (**TTCH**-to-**TTTH**) of the protonated spiropyran (**3**). The bond lengths and Cartesian coordinates of the atoms are given in Angstroms, the torsion angles - in degrees.

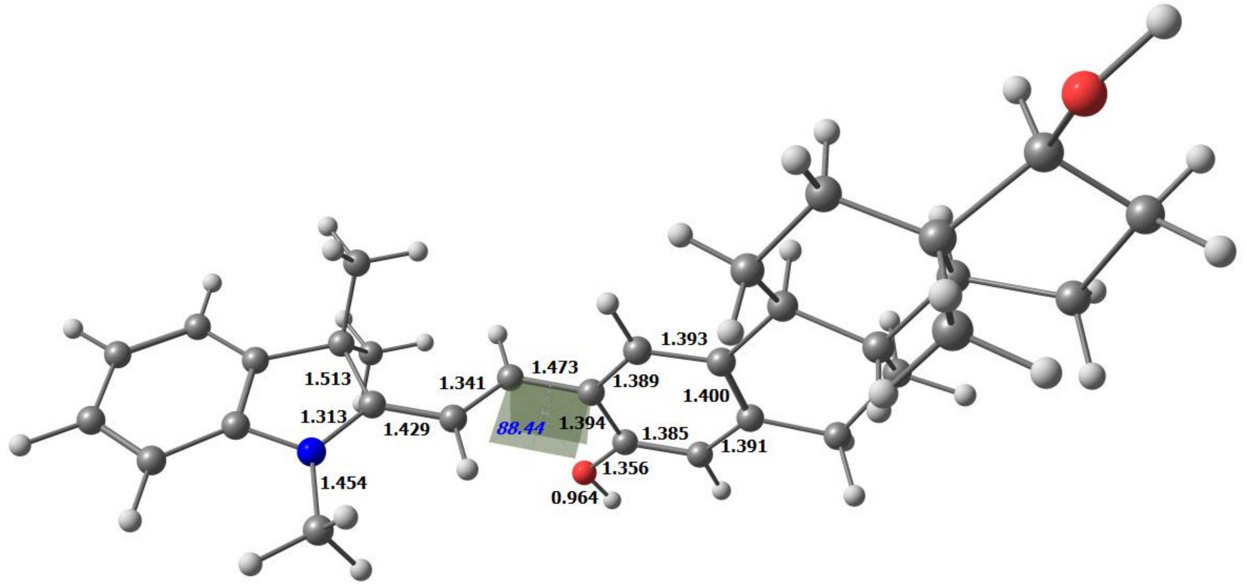

ν_{im} = -86.16 cm^{-1}

Atom	X	Y	Z	Atom	X	Y	Z
8	-4.117710000	10.927733000	8.463625000	1	-1.745807000	9.369014000	7.347623000
8	4.699889000	5.006917000	10.961392000	1	-0.715323000	10.538732000	8.168256000
6	-3.076283000	10.984535000	9.421842000	1	-3.847454000	8.542855000	10.326981000
6	-3.559105000	11.128488000	10.877348000	1	-3.716751000	8.407370000	8.574846000
6	-2.527332000	10.368682000	11.738585000	1	-2.568813000	7.564877000	9.614507000
6	-1.453023000	9.952404000	10.734564000	1	3.128522000	6.097980000	12.798964000
6	-0.497055000	8.826622000	11.066944000	1	2.159147000	7.403063000	8.164969000
6	0.254679000	9.030736000	12.369715000	6	3.884651000	4.537113000	7.697646000
6	1.071496000	7.796040000	12.700030000	6	4.605397000	3.997862000	6.587465000
6	1.866130000	7.281343000	11.530445000	6	5.772524000	4.594258000	5.831676000
6	2.903940000	6.387924000	11.775605000	7	4.274053000	2.837880000	6.068090000
6	3.668858000	5.862055000	10.747440000	6	6.008795000	3.535831000	4.789501000
6	3.383495000	6.222561000	9.431058000	6	5.097089000	2.506934000	4.968355000
6	2.351373000	7.121686000	9.193289000	6	7.011569000	4.742200000	6.726681000
6	1.578347000	7.666961000	10.215434000	6	5.388695000	5.924032000	5.167336000
6	0.506983000	8.695545000	9.914779000	6	3.201196000	1.979685000	6.545627000
6	-0.197178000	8.492071000	8.572291000	6	6.934407000	3.462961000	3.768980000
6	-1.233048000	9.579333000	8.291768000	6	5.059792000	1.383139000	4.164685000
6	-2.229997000	9.712347000	9.433331000	6	6.917836000	2.343528000	2.942433000
6	-3.139494000	8.486256000	9.498082000	6	5.995118000	1.320503000	3.139304000
6	4.181858000	5.691065000	8.313266000	1	3.034400000	3.967011000	8.053941000
1	-4.566472000	11.778572000	8.463185000	1	5.032389000	6.284959000	7.994305000
1	-2.407427000	11.821033000	9.175601000	1	7.656567000	4.255826000	3.611270000
1	-4.555024000	10.687459000	10.964322000	1	7.634262000	2.266028000	2.133274000
1	-3.645524000	12.179004000	11.161967000	1	6.002361000	0.458364000	2.483439000
1	-2.979670000	9.490283000	12.207451000	1	4.346074000	0.583333000	4.310888000
1	-2.120647000	10.985027000	12.541922000	1	3.167857000	1.083719000	5.935219000
1	-0.830140000	10.841467000	10.549698000	1	3.388208000	1.702933000	7.582422000
1	-1.061088000	7.887062000	11.150092000	1	2.248992000	2.502842000	6.466157000
1	-0.439543000	9.241697000	13.187156000	1	4.500066000	5.804758000	4.545475000
1	0.909617000	9.905331000	12.271675000	1	6.216412000	6.245788000	4.533991000
1	0.393762000	6.998610000	13.030002000	1	5.198107000	6.700346000	5.907610000
1	1.745960000	7.983623000	13.540093000	1	7.854357000	5.036828000	6.099900000
1	1.014848000	9.671790000	9.855257000	1	7.254256000	3.796653000	7.214319000
1	-0.665748000	7.502276000	8.557855000	1	6.868323000	5.505268000	7.490551000
1	0.532370000	8.497435000	7.759608000	1	4.803261000	4.853733000	11.907937000

Table S26. Optimized geometry of the protonated cationic form **TTTH** of spiropyran (**3**). The bond lengths and Cartesian coordinates of the atoms are given in Angstroms, the torsion angles - in degrees.

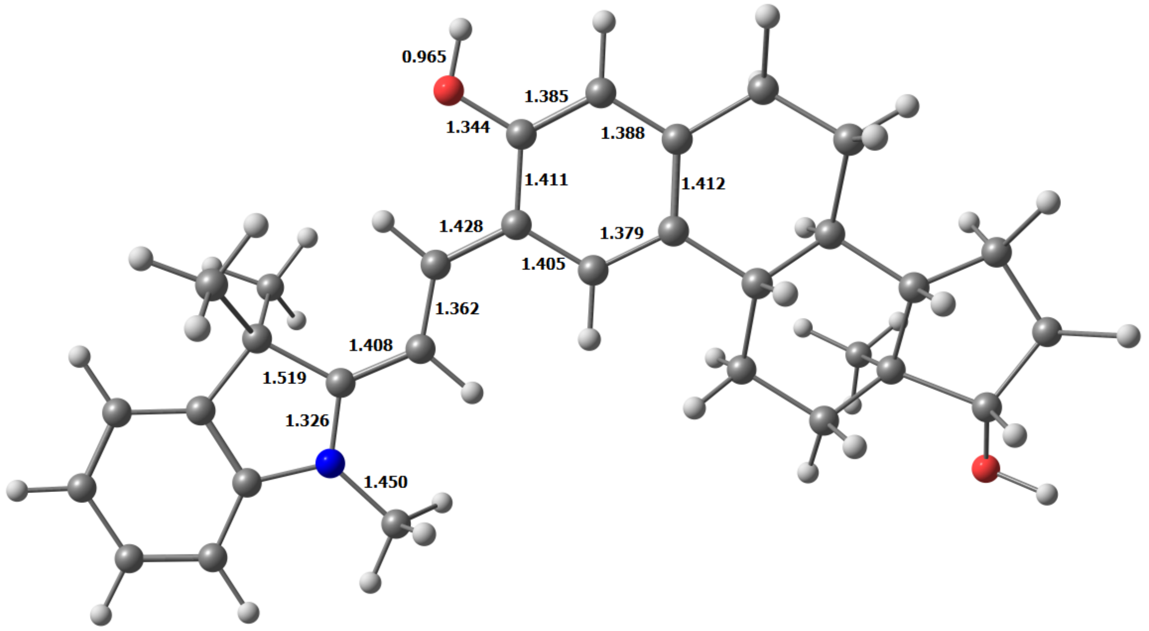

Atom	X	Y	Z	Atom	X	Y	Z
8	-4.826273000	9.326860000	9.026058000	1	-2.429964000	7.847478000	7.860792000
8	5.437362000	6.191109000	10.823245000	1	-1.587883000	9.394227000	7.896259000
6	-3.655584000	9.920671000	9.556264000	1	-3.552212000	7.945661000	11.428840000
6	-3.837722000	10.554095000	10.947989000	1	-3.797741000	7.163279000	9.868388000
6	-2.488817000	10.364643000	11.674933000	1	-2.268799000	6.968942000	10.723926000
6	-1.583782000	9.756072000	10.604534000	1	4.117830000	7.757008000	12.474030000
6	-0.327155000	9.016079000	11.007875000	1	1.877476000	6.925056000	8.176460000
6	0.606719000	9.843784000	11.871767000	6	3.789371000	5.307374000	7.178000000
6	1.767815000	8.995299000	12.352670000	6	4.500056000	4.495047000	6.273096000
6	2.385821000	8.151713000	11.276714000	6	5.883736000	3.885297000	6.417030000
6	3.621025000	7.570740000	11.525891000	7	3.985856000	4.161119000	5.097589000
6	4.240314000	6.757372000	10.592192000	6	6.022303000	3.168961000	5.100575000
6	3.608137000	6.505295000	9.356518000	6	4.870956000	3.359781000	4.351184000
6	2.358889000	7.106005000	9.128356000	6	5.943505000	2.874749000	7.570788000
6	1.731486000	7.920663000	10.047139000	6	6.967278000	4.963229000	6.553101000
6	0.411058000	8.591104000	9.731329000	6	2.674627000	4.559893000	4.623560000
6	-0.485000000	7.779598000	8.794092000	6	7.057102000	2.410594000	4.594361000
6	-1.800192000	8.491659000	8.482656000	6	4.701387000	2.818481000	3.090079000
6	-2.531126000	8.904319000	9.751334000	6	6.911448000	1.852905000	3.326880000
6	-3.063259000	7.678001000	10.490494000	6	5.750481000	2.056608000	2.588608000
6	4.265036000	5.672794000	8.400345000	1	2.813097000	5.650922000	6.860445000
1	-5.462511000	10.027258000	8.853809000	1	5.237266000	5.312791000	8.707769000
1	-3.290393000	10.682698000	8.853643000	1	7.963096000	2.251212000	5.168262000
1	-4.648306000	10.037874000	11.467895000	1	7.711926000	1.253440000	2.909868000
1	-4.123079000	11.605007000	10.868673000	1	5.656911000	1.614310000	1.603967000
1	-2.593704000	9.682942000	12.523612000	1	3.802595000	2.969192000	2.506370000
1	-2.090783000	11.302337000	12.065793000	1	2.501173000	4.121386000	3.646578000
1	-1.258555000	10.582755000	9.953456000	1	1.908133000	4.207183000	5.313654000
1	-0.601494000	8.115281000	11.574248000	1	2.623199000	5.645975000	4.541393000
1	0.073967000	10.253553000	12.733137000	1	6.904836000	5.682320000	5.734533000
1	0.972823000	10.695761000	11.286364000	1	7.945318000	4.480959000	6.517411000
1	1.414179000	8.315703000	13.138926000	1	6.883806000	5.500304000	7.497418000
1	2.544611000	9.608371000	12.818390000	1	6.904013000	2.358716000	7.531354000
1	0.650707000	9.529966000	9.207236000	1	5.147723000	2.133417000	7.480154000
1	-0.676433000	6.798891000	9.242161000	1	5.857446000	3.362931000	8.541033000
1	0.031216000	7.589309000	7.850384000	1	5.758287000	6.443849000	11.697461000

Table S27. Optimized geometry of the protonated form **CCCH*TFA** of spiropyran (**3**) with TFA anion. The bond lengths and Cartesian coordinates of the atoms are given in Angstroms, the torsion angles - in degrees.

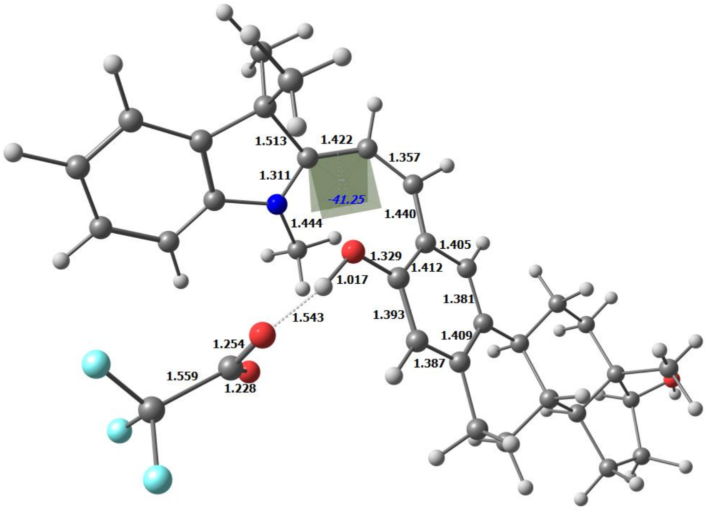

Atom	X	Y	Z	Atom	X	Y	Z
8	-5.199429000	9.540383000	11.106857000	1	-1.892695000	7.853357000	12.209017000
8	4.716985000	5.672223000	8.887697000	1	4.311865000	8.051622000	9.896457000
6	-3.995198000	10.073868000	10.586015000	1	0.262449000	5.742509000	8.375275000
6	-3.477750000	11.318245000	11.331379000	6	3.258951000	3.563960000	7.267449000
6	-1.937590000	11.243478000	11.253049000	6	4.574744000	3.853049000	6.811335000
6	-1.677149000	10.030938000	10.360171000	6	5.703067000	2.848244000	6.887788000
6	-0.328566000	9.344545000	10.389807000	7	4.983543000	4.947874000	6.216593000
6	0.835898000	10.276121000	10.104542000	6	6.838632000	3.637868000	6.301954000
6	2.150458000	9.548323000	10.312513000	6	6.361636000	4.880124000	5.912477000
6	2.189165000	8.188139000	9.673510000	6	5.376980000	1.614761000	6.033811000
6	3.414970000	7.553463000	9.543081000	6	5.959031000	2.443225000	8.341802000
6	3.543712000	6.290849000	8.969656000	6	4.183400000	6.093299000	5.852329000
6	2.379966000	5.636961000	8.508502000	6	8.169137000	3.326310000	6.111420000
6	1.145208000	6.277831000	8.705211000	6	7.154892000	5.849693000	5.332344000
6	1.012542000	7.539417000	9.250094000	6	8.995727000	4.287225000	5.533737000
6	-0.337328000	8.219257000	9.346990000	6	8.495389000	5.527822000	5.151022000
6	-1.504999000	7.253704000	9.556872000	1	2.973806000	2.528880000	7.101174000
6	-2.850805000	7.972538000	9.627983000	1	1.370494000	3.847348000	8.013236000
6	-2.839499000	9.078724000	10.671899000	1	8.564595000	2.360925000	6.406421000
6	-2.769076000	8.488400000	12.079709000	1	10.045870000	4.067082000	5.382106000
6	2.338312000	4.331800000	7.902395000	1	9.160111000	6.260418000	4.709181000
1	-5.900929000	10.177788000	10.943490000	1	6.765946000	6.819110000	5.050791000
1	-4.140490000	10.317800000	9.524449000	1	3.131767000	5.829785000	5.912714000
1	-3.827782000	11.278872000	12.365705000	1	4.407652000	6.923950000	6.524228000
1	-3.873157000	12.235633000	10.890903000	1	4.437232000	6.377854000	4.831599000
1	-1.502967000	11.091390000	12.245084000	1	6.162670000	3.317864000	8.959904000
1	-1.496210000	12.154761000	10.846560000	1	6.818910000	1.772411000	8.378526000
1	-1.833234000	10.359813000	9.320674000	1	5.090015000	1.920524000	8.745477000
1	-0.168947000	8.903487000	11.383734000	1	6.242312000	0.950104000	6.035424000
1	0.797382000	11.155591000	10.752223000	1	5.152555000	1.895073000	5.003303000
1	0.759235000	10.636105000	9.071176000	1	4.526226000	1.074131000	6.451417000
1	2.319654000	9.419210000	11.389117000	1	5.505161000	6.296693000	9.038032000
1	2.994057000	10.140589000	9.946766000	8	6.847865000	7.048536000	9.153500000
1	-0.513857000	8.719300000	8.381066000	8	6.225854000	8.369410000	7.441085000
1	-1.331494000	6.675286000	10.470381000	6	7.021607000	7.901211000	8.250149000
1	-1.545034000	6.530611000	8.739319000	6	8.485355000	8.435710000	8.195922000
1	-3.646488000	7.249587000	9.834607000	9	9.383168000	7.439976000	8.187979000
1	-3.068932000	8.419034000	8.649753000	9	8.726053000	9.185816000	7.117853000
1	-2.733816000	9.262838000	12.847943000	9	8.752149000	9.194391000	9.273899000
1	-3.654723000	7.877931000	12.266109000				

Table S28. Optimized geometry of the protonated form **TCCH*TFA** of spiropyran (**3**) with TFA anion. The bond lengths and Cartesian coordinates of the atoms are given in Angstroms, the torsion angles - in degrees.

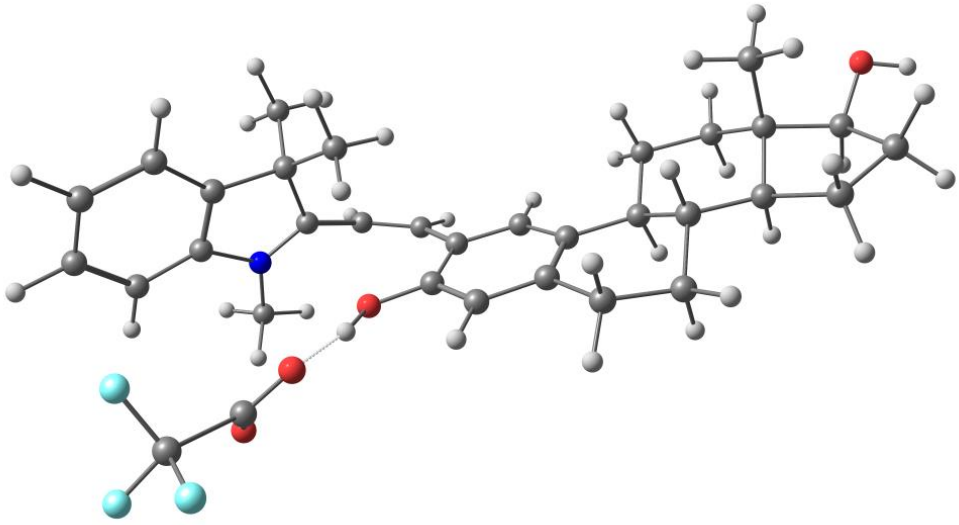

Atom	X	Y	Z	Atom	X	Y	Z
8	-4.205770000	8.337644000	13.016238000	1	-0.530195000	7.432875000	12.215372000
8	3.742646000	6.692544000	5.745912000	1	3.489669000	8.934553000	7.109912000
6	-3.557831000	9.099369000	12.013298000	1	-0.242643000	5.778469000	7.546769000
6	-3.007355000	10.452145000	12.501769000	6	2.609059000	4.025959000	5.561494000
6	-1.729285000	10.709412000	11.675151000	6	4.044809000	4.142603000	5.741000000
6	-1.702753000	9.552013000	10.678088000	6	4.790398000	3.885729000	7.033048000
6	-0.408926000	9.171762000	9.991211000	7	4.886291000	4.219974000	4.750162000
6	0.266936000	10.324749000	9.272679000	6	6.215670000	4.016556000	6.577735000
6	1.625834000	9.888223000	8.758734000	6	6.226043000	4.185578000	5.202070000
6	1.594434000	8.567072000	8.041295000	6	4.487965000	2.417370000	7.407247000
6	2.672353000	8.229891000	7.229140000	6	4.424655000	4.790796000	8.203113000
6	2.733733000	7.017424000	6.556176000	6	4.550495000	4.429439000	3.358657000
6	1.675408000	6.102527000	6.700807000	6	7.410566000	3.968859000	7.266987000
6	0.581889000	6.479988000	7.483802000	6	7.380872000	4.305073000	4.456835000
6	0.514543000	7.680412000	8.176122000	6	8.593711000	4.088612000	6.542354000
6	-0.709158000	8.049188000	8.990257000	6	8.578635000	4.252717000	5.160629000
6	-1.384775000	6.856768000	9.671245000	1	2.316121000	3.138155000	5.004559000
6	-2.631506000	7.261846000	10.457064000	1	0.623810000	4.436456000	5.873837000
6	-2.335095000	8.382094000	11.443616000	1	7.431085000	3.841261000	8.343221000
6	-1.456680000	7.872012000	12.585108000	1	9.543417000	4.057082000	7.063312000
6	1.630863000	4.813039000	6.036183000	1	9.514020000	4.349134000	4.622712000
1	-5.002450000	8.807992000	13.279079000	1	7.362043000	4.444620000	3.383680000
1	-4.257479000	9.268992000	11.182988000	1	3.470465000	4.413247000	3.245397000
1	-2.785301000	10.377005000	13.569062000	1	4.943284000	5.402194000	3.057333000
1	-3.747435000	11.245772000	12.381105000	1	5.006927000	3.643177000	2.757323000
1	-0.840808000	10.691567000	12.312769000	1	4.676024000	5.829287000	7.998573000
1	-1.744628000	11.679811000	11.176544000	1	4.987221000	4.458558000	9.077348000
1	-2.415729000	9.803614000	9.877258000	1	3.361439000	4.714995000	8.431095000
1	0.297203000	8.791009000	10.742263000	1	5.092973000	2.152620000	8.275291000
1	0.384921000	11.180878000	9.942001000	1	4.732273000	1.739012000	6.587830000
1	-0.368638000	10.653611000	8.441093000	1	3.432476000	2.307761000	7.661071000
1	2.313879000	9.795248000	9.608891000	1	4.543071000	7.312206000	5.799629000
1	2.062841000	10.645348000	8.101664000	6	7.996042000	8.453950000	4.830758000
1	-1.447375000	8.469781000	8.288127000	9	8.720912000	7.664682000	5.641181000
1	-0.660604000	6.364761000	10.329417000	9	8.610611000	8.470099000	3.645551000
1	-1.675084000	6.112594000	8.926463000	9	8.068054000	9.697785000	5.329554000
1	-3.044399000	6.388332000	10.971969000	8	6.133399000	7.551525000	3.650726000
1	-3.397570000	7.610349000	9.753062000	8	5.920548000	8.028427000	5.840346000
1	-1.190964000	8.666368000	13.284899000	6	6.522939000	7.955728000	4.742751000
1	-1.990201000	7.101689000	13.145135000				

Table S29. Optimized geometry of the protonated form **TTCH*TFA** of spiropyran (**3**) with TFA anion. The bond lengths and Cartesian coordinates of the atoms are given in Angstroms, the torsion angles - in degrees.

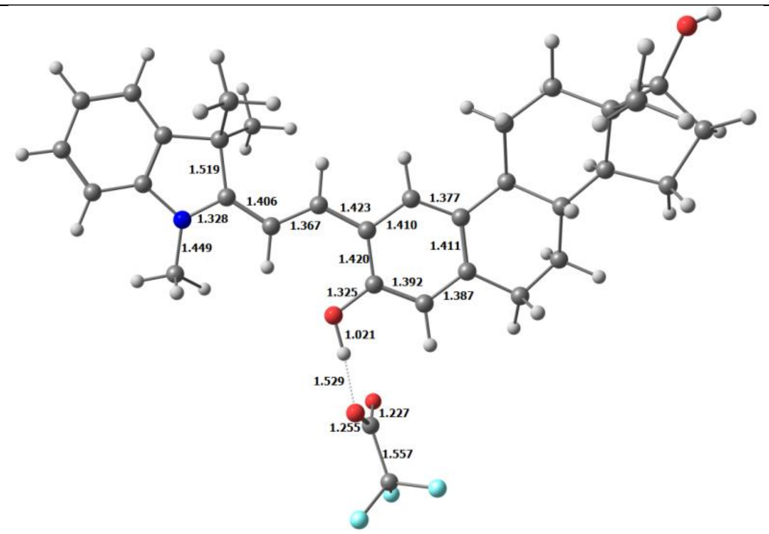

Atom	X	Y	Z	Atom	X	Y	Z
8	-7.840603000	1.754218000	7.719844000	1	-4.960446000	4.181782000	8.615076000
8	2.158319000	5.716061000	6.106991000	1	-0.005478000	6.855888000	5.182403000
6	-7.151946000	2.365735000	6.643259000	1	-0.682173000	2.348316000	7.000589000
6	-7.862700000	3.598583000	6.055812000	6	3.113765000	3.281069000	7.246025000
6	-6.738667000	4.544716000	5.581931000	6	4.030046000	2.369616000	7.800568000
6	-5.461325000	3.740905000	5.820880000	6	3.780070000	1.008514000	8.427603000
6	-4.129330000	4.451350000	5.924630000	7	5.328870000	2.643906000	7.839381000
6	-3.809556000	5.335790000	4.733523000	6	5.175543000	0.612166000	8.829541000
6	-2.549305000	6.134142000	5.007964000	6	6.060562000	1.613974000	8.459074000
6	-1.427507000	5.310557000	5.572008000	6	3.228460000	0.009759000	7.400705000
6	-0.154111000	5.859105000	5.582761000	6	2.880346000	1.103840000	9.665966000
6	0.947231000	5.178478000	6.093624000	6	5.933203000	3.849684000	7.309401000
6	0.750553000	3.871723000	6.612029000	6	5.641284000	-0.521417000	9.461937000
6	-0.558033000	3.346904000	6.597548000	6	7.419871000	1.535542000	8.700557000
6	-1.651295000	4.021436000	6.100289000	6	7.006443000	-0.625605000	9.716917000
6	-3.029415000	3.393397000	6.078319000	6	7.879264000	0.389429000	9.340344000
6	-3.298957000	2.463231000	7.263378000	1	3.501746000	4.219146000	6.880019000
6	-4.691186000	1.835205000	7.209323000	1	1.399429000	2.087270000	7.510030000
6	-5.777642000	2.889648000	7.057771000	1	4.962101000	-1.313651000	9.756456000
6	-5.906171000	3.717063000	8.336397000	1	7.393401000	-1.506679000	10.214974000
6	1.772368000	3.038501000	7.148480000	1	8.938036000	0.290772000	9.548409000
1	-8.664427000	1.394779000	7.377292000	1	8.107707000	2.320491000	8.414238000
1	-6.995823000	1.621923000	5.849277000	1	5.673084000	3.961740000	6.256880000
1	-8.469611000	4.060580000	6.838250000	1	5.582875000	4.719790000	7.865971000
1	-8.539323000	3.316223000	5.246716000	1	7.011979000	3.776294000	7.401493000
1	-6.730757000	5.466807000	6.169902000	1	3.276456000	1.828506000	10.379386000
1	-6.851048000	4.834142000	4.535887000	1	2.844907000	0.125808000	10.148521000
1	-5.369128000	3.029117000	4.985445000	1	1.863420000	1.393448000	9.403650000
1	-4.131907000	5.089718000	6.819331000	1	3.199455000	-0.980597000	7.857955000
1	-4.636163000	6.019045000	4.523418000	1	3.870715000	-0.034258000	6.519534000
1	-3.678137000	4.708350000	3.843537000	1	2.218787000	0.270214000	7.084560000
1	-2.777786000	6.924891000	5.734220000	1	2.161468000	6.664094000	5.728054000
1	-2.197345000	6.648280000	4.109213000	8	2.247866000	8.114941000	5.254593000
1	-3.087732000	2.773614000	5.169345000	8	2.217072000	7.419869000	3.115547000
1	-3.169546000	3.026406000	8.193598000	6	2.210854000	8.260737000	4.008817000
1	-2.560075000	1.659195000	7.286602000	6	2.086745000	9.751672000	3.577209000
1	-4.858188000	1.229925000	8.106069000	9	2.881321000	10.558327000	4.292335000
1	-4.741266000	1.155226000	6.349675000	9	2.381697000	9.947709000	2.290052000
1	-6.648527000	4.511045000	8.237870000	9	0.822787000	10.176838000	3.761802000
1	-6.217419000	3.072680000	9.160705000				

Table S30. Optimized geometry of the protonated form **TTTH*TFA** of spiropyran (**3**) with TFA anion. The bond lengths and Cartesian coordinates of the atoms are given in Angstroms, the torsion angles - in degrees.

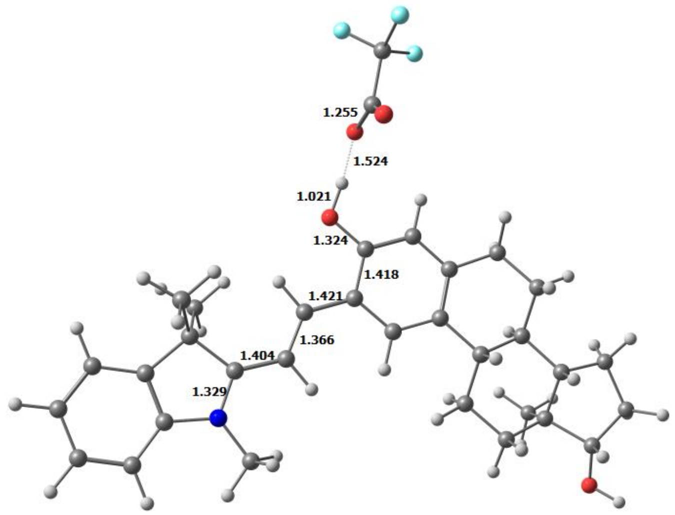

Atom	X	Y	Z	Atom	X	Y	Z
8	-7.840603000	1.754218000	7.719844000	1	-4.960446000	4.181782000	8.615076000
8	2.158319000	5.716061000	6.106991000	1	-0.005478000	6.855888000	5.182403000
6	-7.151946000	2.365735000	6.643259000	1	-0.682173000	2.348316000	7.000589000
6	-7.862700000	3.598583000	6.055812000	6	3.113765000	3.281069000	7.246025000
6	-6.738667000	4.544716000	5.581931000	6	4.030046000	2.369616000	7.800568000
6	-5.461325000	3.740905000	5.820880000	6	3.780070000	1.008514000	8.427603000
6	-4.129330000	4.451350000	5.924630000	7	5.328870000	2.643906000	7.839381000
6	-3.809556000	5.335790000	4.733523000	6	5.175543000	0.612166000	8.829541000
6	-2.549305000	6.134142000	5.007964000	6	6.060562000	1.613974000	8.459074000
6	-1.427507000	5.310557000	5.572008000	6	3.228460000	0.009759000	7.400705000
6	-0.154111000	5.859105000	5.582761000	6	2.880346000	1.103840000	9.665966000
6	0.947231000	5.178478000	6.093624000	6	5.933203000	3.849684000	7.309401000
6	0.750553000	3.871723000	6.612029000	6	5.641284000	-0.521417000	9.461937000
6	-0.558033000	3.346904000	6.597548000	6	7.419871000	1.535542000	8.700557000
6	-1.651295000	4.021436000	6.100289000	6	7.006443000	-0.625605000	9.716917000
6	-3.029415000	3.393397000	6.078319000	6	7.879264000	0.389429000	9.340344000
6	-3.298957000	2.463231000	7.263378000	1	3.501746000	4.219146000	6.880019000
6	-4.691186000	1.835205000	7.209323000	1	1.399429000	2.087270000	7.510030000
6	-5.777642000	2.889648000	7.057771000	1	4.962101000	-1.313651000	9.756456000
6	-5.906171000	3.717063000	8.336397000	1	7.393401000	-1.506679000	10.214974000
6	1.772368000	3.038501000	7.148480000	1	8.938036000	0.290772000	9.548409000
1	-8.664427000	1.394779000	7.377292000	1	8.107707000	2.320491000	8.414238000
1	-6.995823000	1.621923000	5.849277000	1	5.673084000	3.961740000	6.256880000
1	-8.469611000	4.060580000	6.838250000	1	5.582875000	4.719790000	7.865971000
1	-8.539323000	3.316223000	5.246716000	1	7.011979000	3.776294000	7.401493000
1	-6.730757000	5.466807000	6.169902000	1	3.276456000	1.828506000	10.379386000
1	-6.851048000	4.834142000	4.535887000	1	2.844907000	0.125808000	10.148521000
1	-5.369128000	3.029117000	4.985445000	1	1.863420000	1.393448000	9.403650000
1	-4.131907000	5.089718000	6.819331000	1	3.199455000	-0.980597000	7.857955000
1	-4.636163000	6.019045000	4.523418000	1	3.870715000	-0.034258000	6.519534000
1	-3.678137000	4.708350000	3.843537000	1	2.218787000	0.270214000	7.084560000
1	-2.777786000	6.924891000	5.734220000	1	2.161468000	6.664094000	5.728054000
1	-2.197345000	6.648280000	4.109213000	8	2.247866000	8.114941000	5.254593000
1	-3.087732000	2.773614000	5.169345000	8	2.217072000	7.419869000	3.115547000
1	-3.169546000	3.026406000	8.193598000	6	2.210854000	8.260737000	4.008817000
1	-2.560075000	1.659195000	7.286602000	6	2.086745000	9.751672000	3.577209000
1	-4.858188000	1.229925000	8.106069000	9	2.881321000	10.558327000	4.292335000
1	-4.741266000	1.155226000	6.349675000	9	2.381697000	9.947709000	2.290052000
1	-6.648527000	4.511045000	8.237870000	9	0.822787000	10.176838000	3.761802000
1	-6.217419000	3.072680000	9.160705000				

Table S31. Gibbs free energies (**G, a.u.**) of cyclic isomer **Sp**, trifluoroacetic acid (**TFA**) and some protonated **McH** forms of compound (**3**) with TFA anion, and calculated **ΔG** (kcal/mol) of protonation reaction according to the PBE0-D3/def2-TZVP calculations in acetonitrile solutions.

Isomer	G, a.u.	ΔG, kcal/mol
**Sp**	-1407.19203162	-
**TFA**	-526.51557402	-
**CCCH*TFA**	-1933.71079770	-2.0
**TCCH*TFA**	-1933.70440392	2.0
**TTCH*TFA**	-1933.71742607	-6.2
**TTTH*TFA**	-1933.71830021	-6.7
